# LNL-Carbazole Pincer Ligand: More than the Sum of Its Parts

**DOI:** 10.1021/acs.chemrev.3c00202

**Published:** 2023-06-23

**Authors:** George Kleinhans, Aino J. Karhu, Hugo Boddaert, Sadia Tanweer, David Wunderlin, Daniela I. Bezuidenhout

**Affiliations:** Laboratory of Inorganic Chemistry, Environmental and Chemical Engineering, Faculty of Technology, University of Oulu, P.O. Box 3000, FI-90014 Oulu, Finland

## Abstract

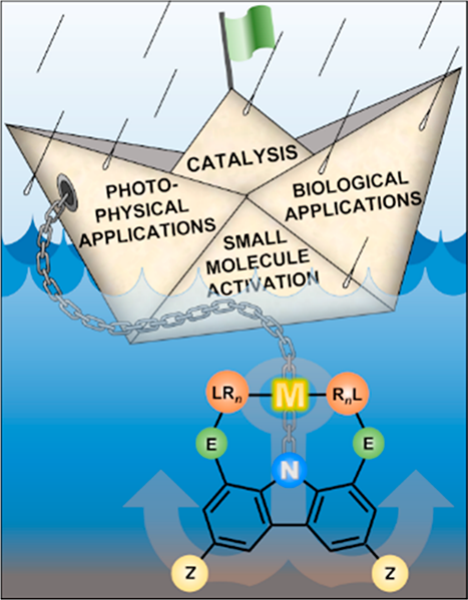

The utility of carbazole in photo-, electro-, and medicinal applications has ensured its widespread use also as the backbone in tridentate pincer ligands. In this review, the aim is to identify and illustrate the key features of the **LNL**-carbazolide binding to transition metal centers (with **L** = flanking donor moieties, e.g., C, N, P, and O-groups) in a systematic bottom-up progression to illustrate the marked benefits attainable from (i) the rigid aromatic carbazole scaffold (modulable in both the 1,8- and 3,6-positions), (ii) the significant electronic effect of central carbazole-amido binding to a metal, and the tunable sterics and electronics of both the (iii) flanking donor **L**-moieties and (iv) the wingtip **R**-groups on the **L**-donors, with their corresponding influence on metal coordination geometry, *d*-electron configuration, and resultant reactivity. Systematic implementation of the ligand design strategies not in isolation, but in a combinatorial approach, is showcased to demonstrate the potential for functional molecules that are not only modulable but also adaptable for wide-ranging applications (e.g., stereoselective (photo)catalysis, challenging small molecule activation, SET and redox applications, and even applications in chemotherapeutics) as an indication of future research efforts anticipated to stem from this versatile pincer assembly, not only for the transition metals but also for *s*-, *p*-, and *f*-block elements.

## 1 Introduction

### 1.1 Carbazole: the Core of the Privileged Pincer

The unique properties of the nitrogen-containing tricyclic 9*H*-carbazole prompted its rapid development in various disciplines such as photo-,^1−5^ electro-,^6,7^ and medicinal chemistry.^1,3,8^ The synthesis of the carbazole backbone itself has been extensively documented,^3^ and the low cost of the precursor carbazole renders elaborate backbone modification economically viable.^3,9−22^

The rigid and stable planar heterocycle boasts with proficient electron donating ability, charge transfer functionality, and excellent biocompatibility.^1,7,23,24^ Its efficient hole transporting capability^7,25,26^ has equated to carbazoles’ success in the fields of photo-^1−5,11,27,28^ and electrochemistry,^6,10,29−31^ also in donor–acceptor systems crucial toward preparation of organic light emitting diodes (OLEDs)^23,31−35^ with photoswitching ability.^36^ The smart electro- and photoactive application of carbazole extends further, with successful utilization in polymers and semiconducting polymers,^5,15,24,37−43^ electrochemiluminescence,^44^ and tailor-made photo(redox) catalysis.^45−47^ Carbazole-based organic compounds have also featured prominently in the field of medicinal chemistry spanning application as anticancer,^8,48−54^ antifungal,^50,55,56^ antioxidant,^57,58^ antiviral,^59^ anti-inflammatory,^58,60^ and antibacterial agents.^50,55,61,62^

This review is based on the distinctive use of the 9*H*-carbazole as the central moiety in the motif of a pincer ligand design, that can exploit the versatility of carbazole applications as listed above, while introducing the inherent advantages of pincer ligands coordinated to a relevant metal center.

### 1.2 Inherent Benefits of the Carbazole-Based Pincer Ligand

**Figure 1:**
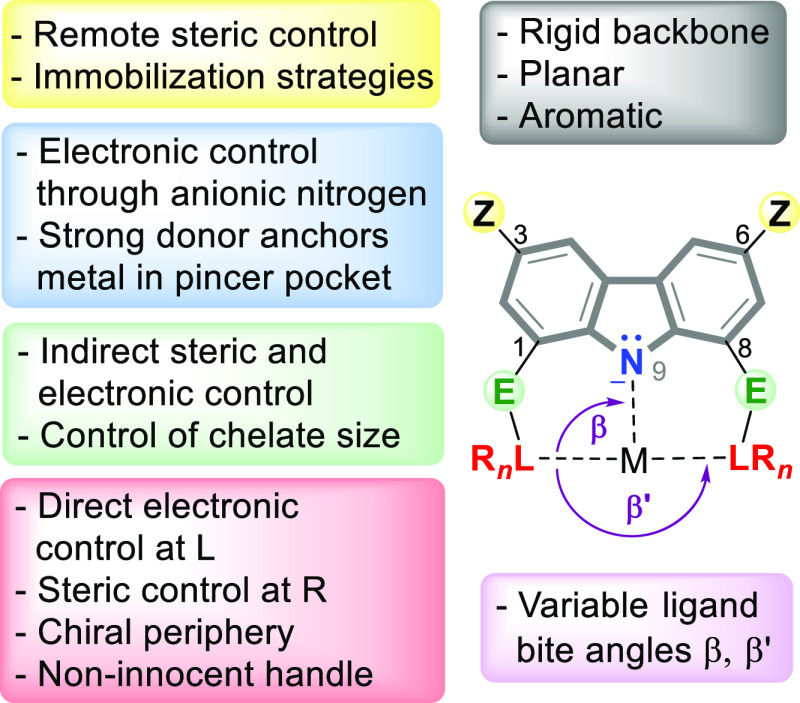
General representation of **LNL**-carbazolide ligand under review.

The privileged pincer ligand platform has indulged a plethora of elements, reactivities, and applications.^63−72^ It has been widely celebrated for the stabilization it imparts to the chelated entity, and more recently, ligand noninnocence accessible through tailored ligand design.^73−82^ This includes redox noninnocence^83−88^ and ligand-metal-mediated processes,^89−98^ which have witnessed an exponential surge in interest due to the reactivity accessible through this multipronged approach to bond activation. Control over 5- or 6-membered chelation at position **E** (as exemplified for the carbazole-based pincer ligand shown in Figure 1) extends the range of reactivity available with a pincer ligand in hand, while introduction of chirality at **R** could lead to stereoselective processes.^99−107^ Immobilization strategies are another possibility, usually by modifying the backbone of the ligand at position **Z** (see Figure 1 per illustration), leading to an immobilized catalyst retaining its selectivity and reactivity while being recycled several times.^108−112^ These attributes, among others, have rendered pincer ligands an attractive platform from which to prepare tailored complexes.

Assembly of carbazole in a pincer allows for a tailored ligand that harnesses the unique properties of carbazole constituting the backbone of the tridentate ligand. Subsequent coordination to a metal or main group element endows unique reactivity to the coordinated species, capitalizing on a pincer environment complemented by the use of a carbazole scaffold. Ligand fine-tuning is further realized through facile modification of control points **Z**, **E**, **L**, and **R** (Figure 1). These attributes have led the way during the design and synthesis of **LNL**-carbazolide coordinated complexes, with the isolated species finding use in a myriad of applications, including small molecule activation, stoichiometric transformation, catalysis, and photoluminescence, which are detailed throughout this review. One of the cornerstones of the tridentate **LNL**-carbazolide is its inherent stabilizing properties as illustrated throughout this review, conferring adequate stabilization to reactive and even elusive species leading to its subsequent isolation. In this respect, the rigid, aromatic carbazole scaffold (modulable in both the 1,8- and 3,6-positions) provides enhanced stability as a result of the connection of the flanking (**E**)**L**-donor groups to aromatic sp^2^-carbon atoms on the 1,8-positions of carbazole, yet wide variation in the bite angles of (**E**)**L**–M–**N** and **L**–M–**L** (β and β′, respectively, Figure 1) can be achieved as shown throughout this text.

**Scheme 1:**
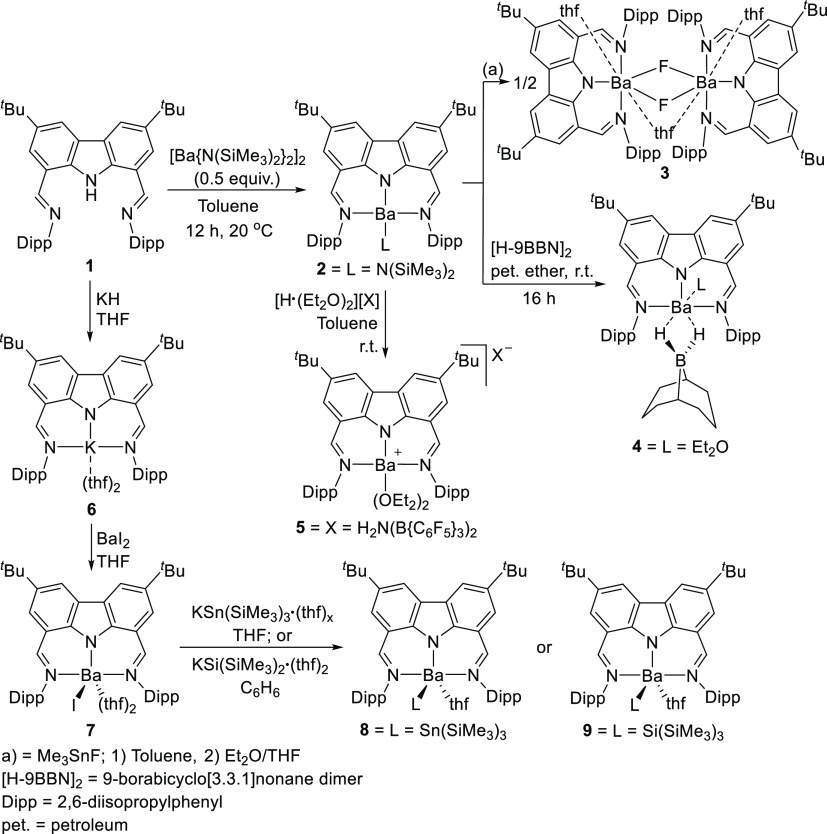
Bis(imine)carbazolide Sufficiently Stabilizing Reactive Barium Complexes

An example of the stabilizing properties of the carbazolide pincer was recently expressed through the synthesis and isolation of molecular barium fluoride and barium stannylide complexes (**3** and **8**, respectively, Scheme 1); a class of compounds previously inaccessible due to inefficient stabilization.^113^ Both thermally stable group 1 metal complexes (Li, Na, and K)^114^ and 3*d*-transition metal complexes (e.g., Co as a hydrophosphination catalyst precursor)^115^ containing the bis(imine)carbazole pincer ligand **1** have been previously reported. When employed to tame the group 2 metals, the resulting barium complex **2** displays catalytic performance comparable to the best catalyst in the benchmark catalytic hydrophosphination of styrene with HPPh_2_.^113^ The use of bis(imine)carbazole **1** inhibits ligand redistribution via the Schlenk equilibrium, a decomposition pathway plaguing the oxophilic and ionic alkaline earth metal complexes, allowing for the isolation of molecular barium complexes **3**–**5** and **7**–**9** (Scheme 1).^116−119^ Ligand scrambling of **2** was inhibited, even at 80 °C. In fact, only in the presence of excess ligand and at a reaction temperature of 80 °C did the homoleptic complex form. A range of Ba^113,120,121^ and analogous group 2 (Mg, Ca, and Sr)^120^ complexes were reported, with further reactivity studies on the Ca^120^ and Ba ( Scheme 1 and Scheme 38, *vide infra*)^113,120,121^ complexes providing insight into this scarcely reported class of compounds (see section 3.3.1 below).^116−119^

**Scheme 2:**
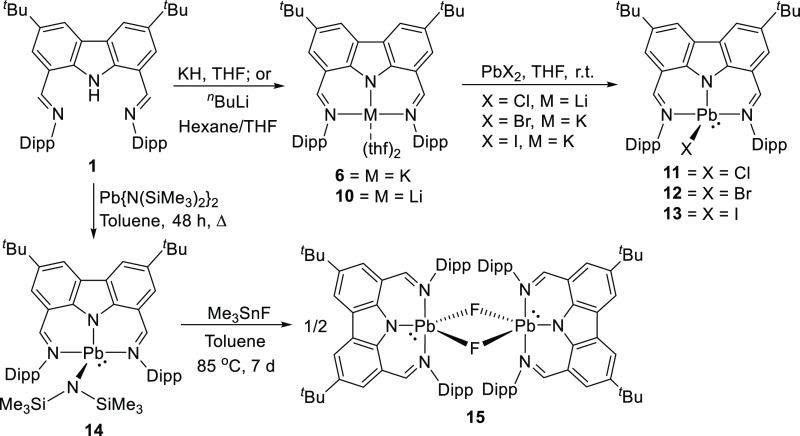
Toward Stable and Soluble Molecular Lead(II) Halides and a Dimeric Lead(II) Fluoride

Yet another hallmark of pincer ligands, including the carbazolide pincer, is its ability to solubilize complexes otherwise insoluble in most solvents, as was the case for a recently reported class of lead(II) complexes.^122^ The chemistry of molecular lead is overshadowed by reports of metallic lead formation due to the decomposition of its organometallic complexes, in addition to its poor solubility generally forming insoluble precipitates.^123−132^ However, the carbazolide sufficiently stabilizes various lead(II) halide complexes (**11**–**13**, Scheme 2), while it was reported that these complexes are well soluble in solvents of low polarity, such as aromatic hydrocarbon and ether solvents.^122^ A rare example of a molecular lead(II) fluoride **15** was isolated by reacting **14** with the fluorinating reagent Me_3_SnF for 7 days at 85 °C (Scheme 2).

These examples of the inherent benefits to be gained from the use of the carbazolide scaffold, and indeed the majority of the reports reviewed here, demonstrate the coordination of the **LNL**-carbazolide pincer ligand in the expected meridional geometry as per the definition of pincer ligands,^133^ as a result of the planar, rigid carbazole backbone. However, the scaffold is sufficiently flexible to allow for facial coordination if enforced by the coordination environment, as demonstrated by singular examples with hindered rotation of the donor **L** and wingtip **R** groups of the **LNL**-carbazolide pincer, with the use of coligands such as pentamethylcyclopentadienyl (see section 4.3.2),^134^ or in trigonal bipyramidal molecular geometries (section 2.1),^135^ leading inevitably to significant changes in the bite angles of the donating ligand sites.

### 1.3 Scope of the Review

This contribution aims to highlight the modality and functionality of the **LNL**-pincer ligand featuring carbazole as the backbone motif, with pincer complex formation through coordination of the carbazole’s anionic nitrogen and the two flanking donor groups. Using relevant examples of complex formation with the tridentate ligand, we will use a “bottom-up” perspective to delineate the key features of the coordinated **LNL**-carbazolide in the following order: (1) the amido nitrogen and its effects on the metal; (2) variations to the flanking donor groups **EL** (with **L** = C, N, P, or O-donor ligands) and their influence on the metal, in addition to the size of the chelate controlled by **E**; and (3) the facile modification of the **R** wingtip groups to incorporate steric bulk, chirality, or even a noninnocent moiety. The constructed carbazole-based scaffold is evaluated in the broader context of related pincer metal complexes where relevant and showcased in selected examples portraying the summative effects of the different building blocks toward various processes. Only **LNL**-pincer ligands featuring a carbazole backbone as the *central* N-donor ligand will be considered. Furthermore, only complexes in which the **LNL**-carbazolide ligand coordinates to the metal at all three coordinating sites will be scrutinized (i.e., carbazolide complexes featuring mono- or bidentate coordination of the carbazole scaffold will not be discussed in this review).

## 2 Electronic Consequences of the Carbazole Backbone

### 2.1 Electronic Effects of the Carbazolide-Nitrogen

**Scheme 3:**
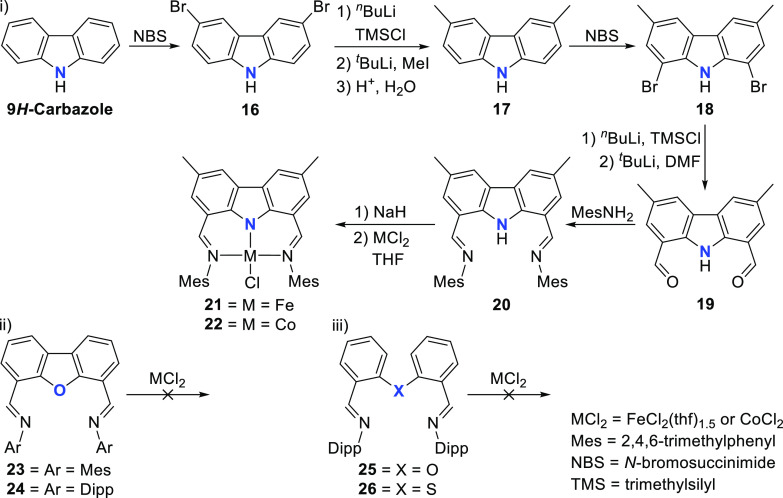
Synthesis of (i) Bis(imine)carbazole Ligand and Reactions of Pincer Ligands (i–iii) with Fe or Co

The report of Gibson et al. in 2003 already demonstrated the requirement for the donor amide of the carbazolide tridentate ligand toward pincer complex formation.^136^ The authors prepared the bis(imino)carbazole pincer ligand precursor **20**, in addition to the analogue ligand featuring an oxygen (**23** and **24**) instead of the amido donor moiety (i and ii, Scheme 3). The dibrominated carbazole **18**, accessed through bromination of the 3,6-dimethylcarbazole **17** with *N*-bromosuccinimide (NBS), was subjected to formylation via quenching of the 1,8-dilithiated intermediate with dimethylformamide (DMF), leading to **19**. A Schiff-base condensation between the dialdehyde **19** and 2,4,6-trimethylaniline (MesNH_2_) yielded the precursor **20**. Deprotonation of **20** with NaH followed by *in situ* coordination of the carbazolide to either FeCl_2_(thf)_1.5_ or CoCl_2_ yielded the complexes **21** and **22**, respectively (i, Scheme 3). Contrasting this result, the attempted coordination of ligands **23**–**26** with either FeCl_2_(thf)_1.5_ or CoCl_2_ did not result in the formation of the targeted complexes, and only the starting material could be isolated (ii and iii, Scheme 3). For **20**, the stronger donor amide in the five-membered pyrrolic heterocycle can be credited as one of the major contributing factors toward pincer complex formation compared to the softer neutral sulfur and oxygen (**23**–**26**) containing analogues, by securing the metal in the pincer pocket with strong amide coordination.^136^ It is worth mentioning that dibenzofuran-based pincer ligands featuring oxazoline donor groups instead of imines as in the analogues **23** and **24**,^137^ or phosphines,^138^ did lead to successful pincer complexes with other metals such as nickel(II) prior to this report.

**Scheme 4:**
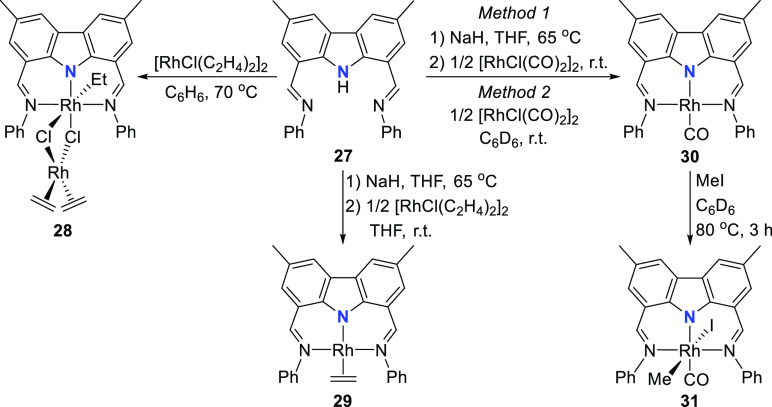
Nucleophilic NNN-Carbazolide Accelerating MeI Oxidative Addition

The anionic nitrogen donor of the carbazole-based NNN-pincer ligand was implicated in the fast oxidative addition of MeI across a rhodium(I) pincer coordinated metal center.^139^ Gibson, Haynes, and co-workers reported complex **30** to oxidatively add MeI across the metal 50 000 times faster compared to the carbonylation catalyst [RhI_2_(CO)_2_]^−^ (Scheme 4). The authors disclosed the deprotonation of **27** with sodium hydride followed by *in situ* coordination of [RhCl(C_2_H_4_)_2_]_2_ and [RhCl(CO)_2_]_2_, leading to the isolation of the NNN-pincer coordinated rhodium(I) complexes **29** and **30**, respectively. The rhodium carbonyl complex **30** could also be prepared through the direct metalation of ligand **27** with the rhodium precursor [RhCl(CO)_2_]_2_. Interestingly, reacting **27** with one equivalent of the dimeric precursor [RhCl(C_2_H_4_)_2_]_2_ yielded a dinuclear mixed valent complex **28** (Scheme 4). The dinuclear complex **28** features an NNN-pincer coordinated octahedral Rh^III^ metal, bridged to a square planar Rh^I^ metal via two chlorido coligands. The carbonyl stretching frequency of **30** was measured to be 1980 cm^–1^,^139^ at a significantly higher energy than the rhodium(I) BIMCA (3,6-di-*tert*-butyl-1,8-bis(imidazol-2-ylidene-1-yl)carbazolide) complex **194** with a ν(CO) band at 1916 cm^–1^ (*vide infra*, Figure 5).^140^ Reacting **30** with excess MeI in C_6_D_6_ at room temperature resulted in slow formation of the octahedral rhodium complex **31**, but complete conversion of **30** to **31** was noted after 3 h at 80 °C.^139^ The carbonyl stretching frequency shifted considerably from 1980 cm^–1^ for **30** to 2083 cm^–1^ for **31**, an observation consistent with weaker back-donation going from square-planar Rh^I^ to octahedral Rh^III^ complexes. Not only was the rate of oxidative addition faster than observed for the commercial carbonylation catalyst,^141^ but also it was reported to be faster than rhodium complexes coordinated by neutral donor ligands such as 2,2′-bipyridine,^142^ 1,2-bis(diphenylphosphino)ethane,^143,144^ or PEt_3_.^145^

**Scheme 5:**
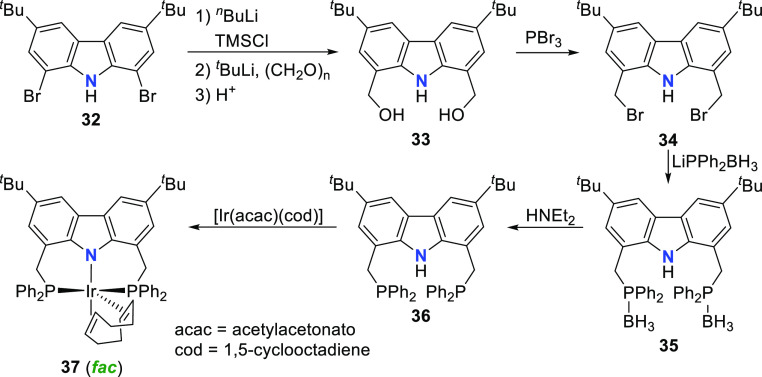
Synthesis of a PNP-Carbazole Ligand and Coordination to Ir^I^

Activity in the benchmark catalytic transfer dehydrogenation reaction between cyclooctane (COA) and *tert*-butylethylene (TBE) at high temperatures (200 °C) to form cyclooctene (COE) and *tert*-butylethane (TBA) was communicated in the seminal report of a dihydrido iridium complex with a PCP-ligand.^146^ Subsequent DFT calculations showed that the thermodynamic favorability of oxidative addition of nonpolar substrates like H_2_ or RH to the fragment XML_2_ (M = Ir, Rh) increases as the σ-donating ability of coordinating group X decreases.^147,148^ These results prompted the interest of both the groups of Gade^135^ and Goldman and Brookhart^149^ to prepare PNP-pincer ligands featuring the more rigid carbazole backbone for complexation to iridium, compared to the diphenylamide pincer ligand studied by Ozerov et al.^150−153^ The use of hybrid ligands containing both soft phosphorus and hard amido donor atoms, a 6-membered chelate ring-size and flexibility introduced by using methylene spacers between the carbazole and the phosphines, were additionally rationalized for coordination to transition metals with large atomic radii by Gade et al.^135^ The ligand **36** was prepared from the starting material 1,8-dibromo-3,6-di-*tert*-butyl-9*H*-carbazole **32** (Scheme 5).^154^*N*-protection by a TMS group was followed by bromine substitution with hydroxymethylene and subsequent reaction with *p*-formaldehyde to yield **33**. The carbazole-diol was then treated with PBr_3_ to yield the key intermediate **34**. A borane-protected ligand **35** was prepared by reaction of **34** with the lithium-diphenylphosphine-BH_3_ adduct, which delivered the protioligand **36** after deprotection. The ligand precursor **36** was treated with [Ir(acac)(cod)] (acac = acetylacetonato, cod = 1,5-cyclooctadiene) (Scheme 5) to give the corresponding complex **37**.^135^ Unlike the previously reported *d*^8^-metal complexes bearing this ligand in a meridional coordination mode,^154^ the molecular structure of **37** revealed a facial coordination of the distorted carbazole backbone for the trigonal bipyramidal coordination geometry.^135^ The strongest σ-donors occupy the axial positions, as demonstrated by the shorter axial diene bond (C=C bond length 1.408(7) Å) compared to the equatorial diene bond (C=C bond length 1.443(7) Å) of the cod ligand.^155^

**Scheme 6:**
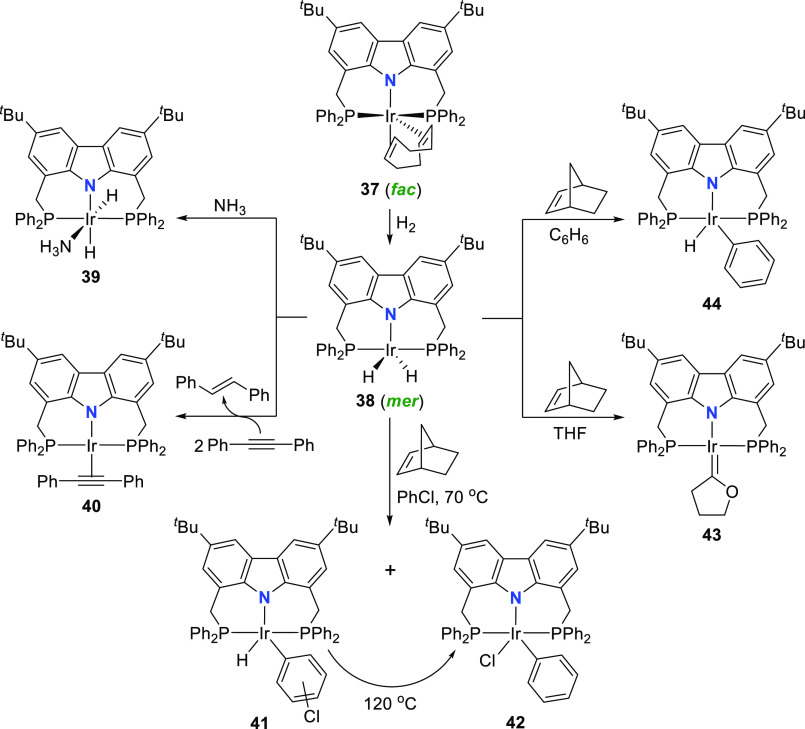
Bond Activation Reactivity of a Dihydrido PNP-Ir^III^ Complex

The iridium dihydrido complex **38** was accessed by reacting **37** with hydrogen at 10 bar (Scheme 6),^135^ while avoiding salt formation by using triethylborohydride sources.^146,156,157^ The trigonal bipyramidal geometry of the dihydrido iridium(III) complex **38** displays the PNP-pincer ligand coordinated in the more typical “pincer”-like *mer*-fashion.^135^ Although reaction of **38** with a solution of ammonia in THF resulted in the formation of the ammine complex **39** with no sign of any N–H activation, bond cleavage of both C–H and C–Cl bonds proceeded facilely (Scheme 6). Initial reactivity studies revealed that stoichiometric reaction of **38** with diphenylacetylene hydrogenates the alkyne selectively to *trans*-stilbene, with isolation of the corresponding alkyne complex **40** (Scheme 6). If the reaction is carried out under catalytic conditions (excess H_2_ and alkyne), complete hydrogenation of diphenylacetylene is observed. Similarly, reaction of norbornene with **38** led to the hydrogenation of norbornene and the formation of a reactive species that subsequently reacts with the reaction solvent employed. In the case of benzene as solvent, the C–H activated phenyl hydrido complex **44** is obtained, while the use of chlorobenzene as solvent indicates the formation of three different C–H activated products **41** and the C–Cl oxidative addition product **42**. Complex **42** is thermodynamically favored, as shown by its selective formation following heating at 120 °C (Scheme 6). Finally, double C–H activation is achieved with the use of tetrahydrofuran as solvent, and Fischer carbene complex **43** is isolated.^135^

**Scheme 7:**
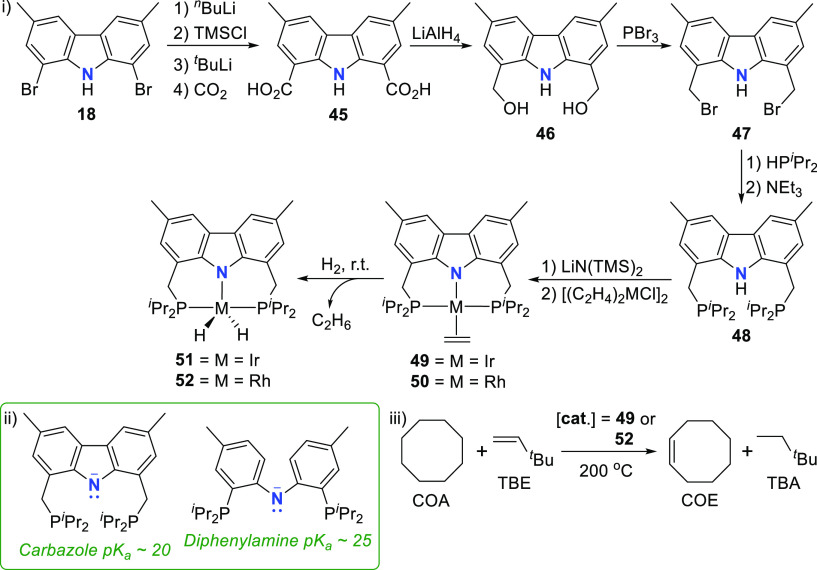
Synthesis of (i) Iridium(I) and Rhodium(I) Olefin Complexes with a PNP-Carbazolide Pincer Ligand and (ii) Comparative p*K*_a_ Values for the Bis(diisopropylphosphino)carbazole and Corresponding Bis(diisopropylphosphino) Diphenylamine Ligands Employed in (iii) the Transfer Hydrogenation of COA with TBE to Form COE and TBA

The closely related protioligand **48** with the carbazole backbone methylated in the 3,6-positions, and the phosphine donor moieties containing isopropyl substituents instead of the phenyl groups reported by Gade et al.,^135^ was prepared in a modified sequence, whereafter stepwise lithiation and coordination with the ethylene metal dimers (iridium(I), rhodium(I)) resulted in the formation of the corresponding olefin complexes **49** and **50** of the group 9 metals (i, Scheme 7).^149,158^ Treatment of the olefin complexes with hydrogen atmosphere at room temperature readily displaces the hydrogenated ethane, and the corresponding dihydrido metal complexes **51** (iridium(III))^149^ and **52** (rhodium(III))^158^ are formed. On the basis of the p*K*_a_ values of the neutral ligands, shown in Scheme 7, the central nitrogen in **48** was expected to be a weaker σ-donor than the corresponding nitrogen in the diphenylamine-PNP ligand previously employed by Ozerov et al.^150−153^ in the benchmark transfer dehydrogenation reaction of COA with TBE. The more weakly σ-donating group at the central position of the pincer ligand was anticipated to favor the thermodynamics of C–H and/or H–H addition to 14-electron iridium-pincer fragments implicated in the well-known mechanism of the reaction (iii, Scheme 7).^159^ The use of **49** as the catalyst in this transformation, however, proved ineffective, with experimental and computational investigations indicating that hydrogenation of TBE is the rate-limiting step. Although TBE does insert into an Ir–H bond of **51**, reductive elimination from the resulting Ir^III^ alkyl hydride is thermodynamically very unfavorable and the +3 oxidation state is maintained.^149^ This result contrasts with prior studies of alkane transfer hydrogenation employing catalysts with PCP-pincer ligands where the iridium(III) alkyl hydride or dihydride is not thermodynamically favored over the 14-electron iridium(I).^159^ On the other hand, it was found that the Rh^III^ state was not sufficiently accessible to allow an effective catalytic cycle based on the Rh^I^/Rh^III^ couple for the PCP-ligand.^160^ On the basis of these reports as well as their results with PNP-iridium,^149^ the groups of Goldman and Brookhart investigated the use of the rhodium analogue **50** as catalyst for alkane transfer dehydrogenation.^158^ As the thermodynamics are biased more toward the +1 oxidation state for rhodium than iridium,^161^ it was anticipated that the relatively high stability of the Rh^III^ analogue would not preclude transfer hydrogenation. In support of the hypothesis, complex **52** was found to be an active catalyst for the dehydrogenation of COA with TBE, achieving TOFs (TOF = turnover frequency) of up to 10 min^–1^, as the first example of a rhodium-based catalyst that does not require light or hydrogen atmosphere for this transformation.^158^

### 2.2 Metalloradical Reactivity Enabled by the Carbazolide

**Scheme 8:**
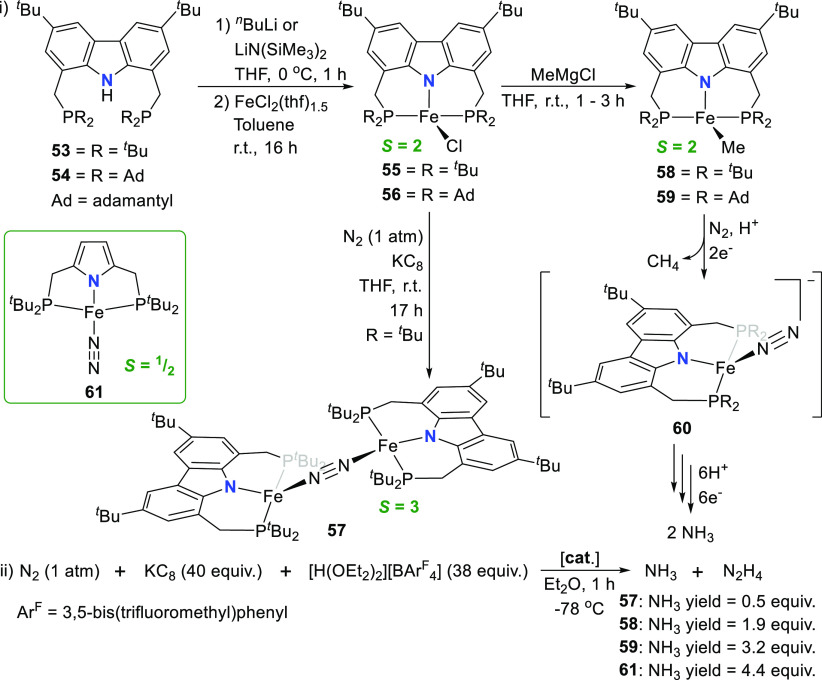
Synthesis of (i) High-Spin PNP-Pincer Complexes of Iron for (ii) Catalytic Reduction of Dinitrogen

The group of Nishibayashi reported the synthesis of iron complexes coordinated by a monoanionic PNP-carbazolide pincer ligand, as catalysts for the fixation of nitrogen.^162^ The protioligands **53** and **54** with bulky *tert*-butyl or adamantyl (Ad) substituents on the phosphines, respectively (i, Scheme 8), were prepared following a modified procedure,^154^ followed by salt metathesis reaction of the *in situ* generated lithium complexes to yield the Fe^II^–Cl complexes **55** and **56**. The complexes exhibit distorted tetrahedral geometries around the iron atoms (τ_4_ = 0.79 for R = ^*t*^Bu and τ_4_ = 0.80 for R = Ad, where τ_4_ = 0.00 for perfect square planar geometry and τ_4_ = 1.00 for tetrahedral geometry),^163^ and the solution magnetic moments determined were consistent with a high spin *S* = 2 electronic configuration.^162^ Reduction of **56** with KC_8_ under nitrogen atmosphere failed to deliver identifiable products for the adamantyl-substituted complex, while a dinitrogen-bridged diiron complex **57** (*S* = 3) was isolated from the corresponding reduction of the ^*t*^Bu-substituted complex **55** (i, Scheme 8). Alternatively, reaction of **55** or **56** with MeMgCl afforded the corresponding methyl-complexes **58** and **59** in both cases. As for **55** and **56**, a high spin tetrahedral geometry around the iron(II) ion was found, in sharp contrast to the low-spin pyrrole-based pincer Fe^I^ complex analogues with geometry indices τ_4_ of 0.11–0.13, e.g., **61** (Scheme 8) previously reported by the same group.^164^ The size of the chelate ring (*vide infra*, section 3.1) rather than the nature of the N-donor, was attributed as the cause of the geometry distortion. In the case of the rigid pyrrole-based PNP-ligand, square planar complex geometry is favored to prevent the formation of a dinuclear structure as found for **57**. The activity of the iron complexes **57**–**59** and **61** in the catalytic reduction of dinitrogen to ammonia and hydrazine was probed using KC_8_ as reductant and [H(OEt_2_)_2_]BAr^F^_4_ (Ar^F^ = 3,5-bis(trifluoromethyl)phenyl) as proton source under atmospheric nitrogen pressure (ii, Scheme 8). The highest yield of 4.4 equiv of ammonia and 0.2 equiv of hydrazine, based on the iron atom of the catalyst, was obtained for **61**. However, a catalyst deactivation pathway for **61** was confirmed to occur via pyrrole-backbone protonation,^164^ rationalizing the use of the central carbazole moiety as a replacement central donor in the pincer scaffold.^162^ The strategy showed limited success as the pronounced influence of the molecular structure on the catalytic activity led to significantly lower yields obtained in the nitrogen reduction (ammonia yield < 0.5 equiv for **57**, 1.9 equiv for **58**, and 3.2 equiv for **59** with R = Ad) compared to the ammonia yield (4.4 equiv) for **61**,^164^ although the possible formation of the corresponding anionic mononuclear iron(0) dinitrogen complexes **60** under catalytic reaction conditions (Scheme 8) was proposed.^162^

**Scheme 9:**
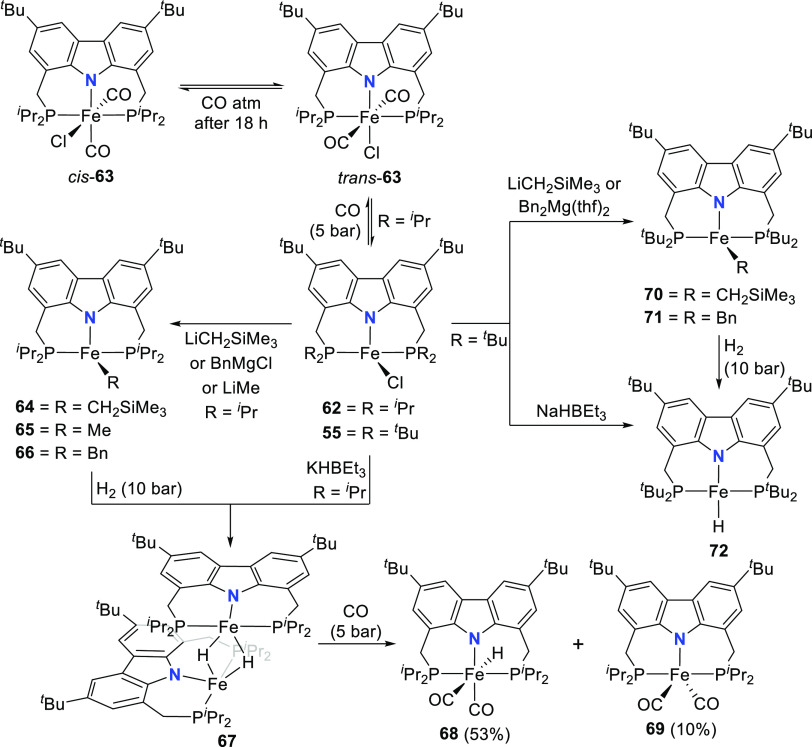
Synthesis and Reactivity of High-Spin PNP-Pincer Hydrido Complexes of Iron Containing a Carbazole-Based Ligand

The related work of Gade et al. demonstrates that the reactivity of the iron complexes is governed also by the steric effects of the wingtip groups (phosphine substituents),^165−167^ (*vide infra*, section 4.1) as well as very prominently by the N(carbazolide)–Fe interaction, which leads directly to metalloradical reactivity.^168,169^ Their interest centered on the isolation and characterization of a paramagnetic, high-spin iron hydrido complex as the key intermediate in the catalytic cycle of iron-catalyzed olefin hydrogenation,^170−172^ in contrast to the known intermediate spin Fe^II^-hydrido complex congener of **61** reported by Nishibayashi et al.^164^ Protioligands **312** (*vide infra*, Scheme 46)^173^ and **53**,^162^ containing diisopropyl- or *tert*-butyl-substituted phosphine donor moieties, respectively, were employed as ligand precursors for the synthesis of the corresponding Fe^II^–Cl complexes **62**(167) and **55**(166) (Scheme 9). The complexes **62** and **55** feature distorted tetrahedral geometry (e.g., for **62** a geometry index of τ_4_ = 0.77^163^ is observed where the acute P–N–P bite angle of 84.03(6)° is enforced by the rigidity of the carbazole backbone),^167^ similar to **56**(162) with high spin state of *S* = 2 determined also in this case. Reaction of **62** with a strong field ligand (CO) results in a change of spin, and the diamagnetic dicarbonyl complexes *trans***-63** and *cis*-**63** are in equilibrium (Scheme 9).^167^ Alkylation of **62** yields again distorted tetrahedral complexes **64**–**66** (Scheme 9) with solution magnetic moments measured that are consistent with a high-spin state. If chloride substitution is effected by reaction of diisopropyl-substituted **62** with KHBEt_3_, a complex displaying reduced magnetic susceptibility with significant antiferromagnetic coupling is observed to form, suggesting that the dimeric structure of the isolated complex **67** is maintained in solution. **67** is a dinuclear hydrido complex, also accessible from the hydrogenation of the alkylated complexes **64**–**66** (Scheme 9), with a highly distorted square-pyramidal geometry around each of the iron centers bridged by two H-atoms. The steric demand of the isopropyl-phosphino substituents results in a twist of the two molecular fragments to give a torsion angle of N(carbazolide)–Fe–Fe–N(carbazolide) of 88.81(12)° with respect to each other. Conversely, a similar reaction sequence for the bulkier *tert*-butyl-substituted **55** yielded a monomeric, square planar Fe^II^-hydrido complex **72** with intermediate spin (Scheme 9),^166^ confirmed by the geometry index τ_4_ = 0.15.^163^ Treatment of **67** with excess CO (g) yielded a mixture of diamagnetic complex **68** and the iron(I) complex **69** as a minor byproduct.^167^ Attempts to prepare **69** by an independent route via reduction of **62** proved unsuccessful.

The alkyl-analogues of **64**–**66**, high-spin complexes **70** and **71**, were obtained using similar alkylating agents and analogously to **64**–**66** feature distorted tetrahedral geometry (Scheme 9).^166^ Hydrogenation of **70**–**71** also leads to the formation of Fe^II^-hydrido **72** as a first example of a metal hydride with a paramagnetic ground state for which the hydrido ligand is directly detectable via solution ^1^H NMR spectroscopy. Extensive DFT calculations were employed for full assignment of the paramagnetic complexes, with unprecedented shifts of the recorded hydride resonances.^166−169,174^

**Scheme 10:**
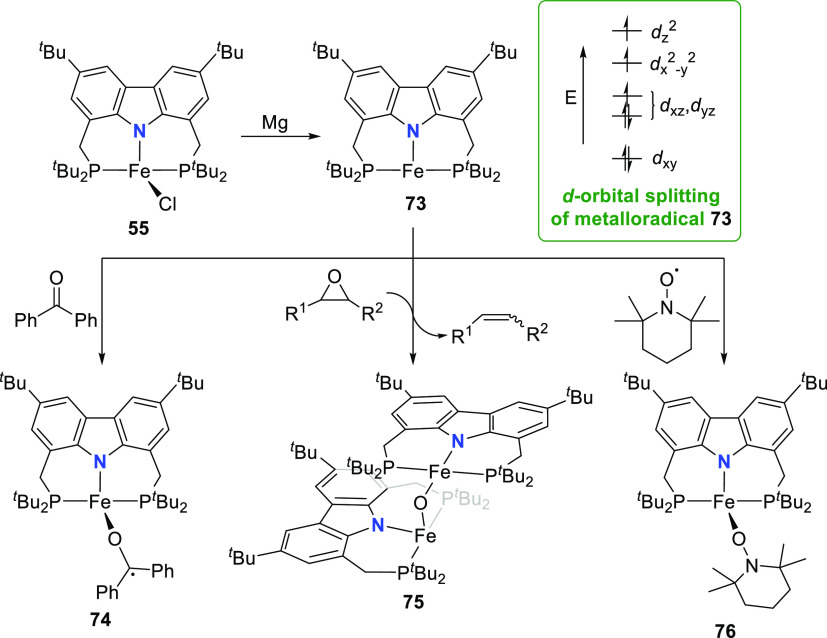
Synthesis and Metalloradical Reactivity of a T-Shaped Fe^I^ Complex

Remarkably, **55** could be reduced to a “naked” T-shaped Fe^I^ complex **73** stabilized by the PNP-pincer ligand with ^*t*^Bu-substituted phosphine donors (Scheme 10).^168^ Treatment of the precursor Fe^II^-chlorido **55** with excess magnesium powder in the absence of a nitrogen atmosphere results in the formation of paramagnetic **73**. The complex is revealed to have a high-spin electronic structure, confirmed by computed spin densities. The majority of unpaired spin is localized around the vacant coordination site to inform a metalloradical character of the iron center with assigned oxidation state of +1. This corroborates the observed chemical inertness of **73** with σ-donors such as THF and NEt_3_ and resembles the electronic “remote basicity” effected by the antibonding nature of the metal-carbazolide nitrogen in the Kohn-Sham HOMO calculated for T-shaped Au-CNC-carbazolide complexes (*vide infra*, section 2.3). The HOMO has *d*_z^2^_ character and is singly occupied (see Scheme 10),^169^ and its Fe–N antibonding character is further enhanced by the unpaired electron spins in the antibonding *d*_x^2^–y^2^_ and nonbonding *d*_*xz*_/*d*_*yz*_ orbitals so that the half-filled orbital is effectively blocked for ligand binding.^175^ In addition, the calculated LUMO of **73** is oriented orthogonally to the FeNP_2_-plane and effectively shielded by the *tert*-butyl groups to further explain the observed reluctance toward adduct formation at the vacant coordination site.^168^

The metalloradical character of Fe^I^ complex **73** in conjunction with its resistance to Lewis acidic reactivity prompted the investigation of its single electron redox chemistry (Scheme 10). Reaction of benzophenone with **73** gives the thermodynamically stable iron benzophenone ketyl radical complex **74** with an expected (distorted) tetrahedral arrangement around the high-spin iron(II) center.^168^ However, end-on coordination of the ketyl ligand is unprecedented in iron chemistry.^176^ DFT analysis, EPR spectroscopy, and solution magnetic moments measured were consistent with a high-spin iron(II) *d*^6^ metal with an antiferromagnetically coupled ketyl radical.^168^ The stability of the alkoxide-Fe^II^ bond led to the assumption that ring-opening reaction with a strained cyclic ether would overcome the inertness of **73** observed with other oxygen-donor ligands. Testing of this hypothesis by addition of various epoxides to **73** generated the rare example of an oxido-bridged diferrous complex **75** and the secondary reaction product mixtures of *trans*- and *cis*-alkenes (Scheme 10). Such single-electron transfer (SET) to a ligand from T-shaped **73** was further demonstrated by the reaction with the stable radical 2,2,6,6-tetramethylpiperidinyloxyl (TEMPO) to give the high-spin Fe^II^ alkoxide complex **76** (Scheme 10)^169^ with elongated O–N bond (1.423(3) Å), compared to free TEMPO (1.296(5) Å).^177^

**Scheme 11:**
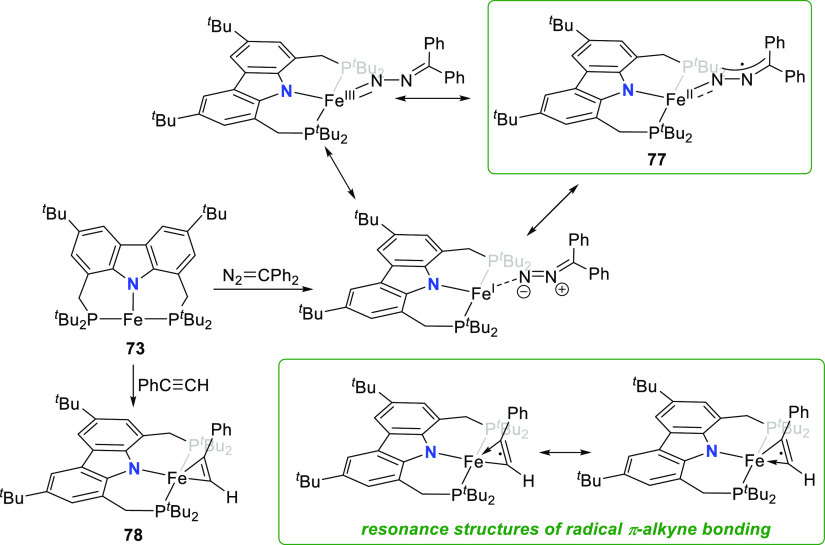
Charge Transfer Reactivity Mediated by a T-Shaped Fe^I^–PNP Complex

Extension of the SET reactivity was done by reaction of **73** with phenylacetylene (Scheme 11). Side-on coordination of the alkyne in the resulting complex **78** was confirmed in the molecular structure, with the steric demand of the phenyl substituent leading to an elongation of the Fe–P bond length on the side of the molecule to which the phenyl substituent is oriented (2.5662(6) Å) compared to the Fe–P bond distance of 2.3593(5) Å on the opposite side.^169^ A quartet ground state with three unpaired electrons was determined from the effective magnetic moment measured. This could indicate either a high-spin Fe^I^ or an intermediate-spin Fe^II^ with the unpaired spin density primarily located on the iron, if the π-alkyne was coordinated as a closed shell unit (either neutral or dianionic, respectively). Computed spin density distribution however showed significant localization of the spin density on the π-alkyne of **78** to indicate delocalization from the metal into the C≡C-π- and π*-orbitals, as shown in the representative resonance structures drawn in Scheme 11.

The charge transfer reactivity of **73** was further illustrated by using diphenyl diazomethane as an electron acceptor.^169^ The complex **77** that forms contains a diazoalkane ligand coordinated end-on, with an Fe–diazomethane-N bond length indicative of a bond order exceeding one (1.765(2) Å), in an amido/imido-type coordination. Moreover, the bent angle of the coordinated diazoalkane CNN unit contrasts with the expectation of a linear CNN unit for a neutral bound ligand. Unlike the decoupled organic radical species found to bind in **74**,^168^ the DFT modeled spin density of **77** is in accordance with an *S* = 2 iron(II) antiferromagnetically coupled with the diazoalkanyl radical.^169^ Based on the computed spin density and the tetrahedral coordination sphere of **77**, the resonance form of a diazomethane ligand bonded in an amido-fashion to a high-spin iron(II), rather than the resonance structures depicting a neutral donor bound to Fe^I^ or a formal iron(III) center, is believed to be a more appropriate description of the bonding in **77** (Scheme 11).

**Scheme 12:**
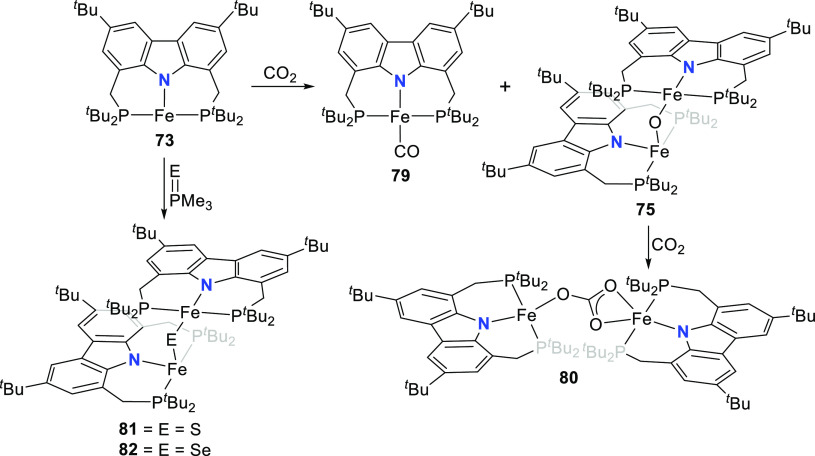
(i) Deoxygenation of Carbon Dioxide and (ii) Chalcogen Abstraction Mediated by a T-Shaped Fe^I^–PNP Complex

The ability of **73** to act as an oxygen atom abstractor was demonstrated by its reaction with epoxides to release the corresponding alkene and oxygen-bridged diiron(II) complex **75** (Scheme 10).^168^ The same oxophilicity resulted in formation of a reaction mixture containing both square planar monocarbonyl complex **79** and dinuclear **75**, following reaction of **73** with carbon dioxide (Scheme 12).^169^ Continued reactivity of **75** with CO_2_ leads to a further CO_2_-to-CO transformation resulting in complex **80**, a dinuclear μ-carbonato iron complex. The μ–κ:^2^κ^1^ coordination mode of the carbonato ligand yields a complex with one molecular fragment with a distorted tetrahedral geometry (τ_4_ = 0.79) and the other with distorted square pyramidal geometry (τ_5_ = 0.25) around the iron centers. Given the propensity of **73** to act as a chalcogen abstractor, the viability of similar reactivity with the heavier chalcogens (S and Se) was investigated by reaction of half an equivalent of trimethylphosphine sulfide or selenide, respectively, with **73** (Scheme 12).^169^ The corresponding dinuclear bridged sulfido **81** and selenido complexes **82** were isolated, with the pincer complex units oriented at approximate right angles to each other. Strong antiferromagnetic coupling between the iron centers was confirmed by solid-state magnetometry to result in linearly increasing molar susceptibility above 50 K.

**Scheme 13:**
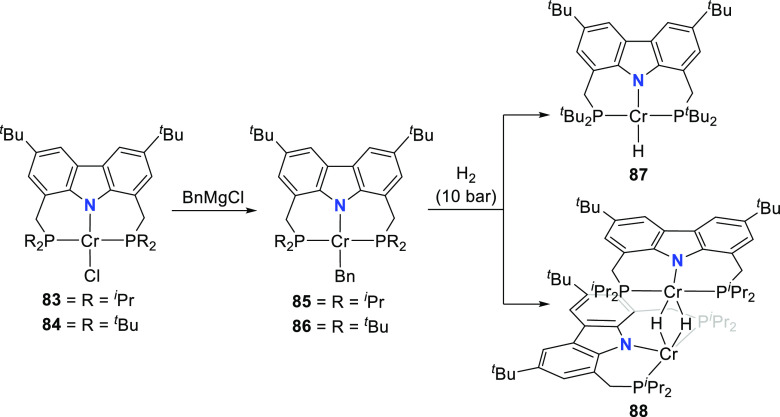
Synthesis of Di- and Mononuclear PNP-Pincer Complexes of Chromium(II) Hydride

The versatile reactivity of T-shape complex **73** obtained from reduction of square planar, mononuclear iron(II)-hydrido **72** (Scheme 9–10) preceded investigation of the mid-first row transition metal chromium.^178^ As for its group 8 analogue, very few examples of low-valent Cr^II^-hydrido complexes have been structurally characterized,^179−184^ due to the challenge of stabilizing the open-shell hydride complexes. A similar synthetic methodology was followed to prepare chromium(II) chlorido complexes **83** and **84** (Scheme 13), from the isopropyl- and *tert*-butyl-substituted protioligands **312** and **53**, respectively.^178^ Both complexes were found to adopt high-spin ground states with four unpaired electrons in solution and displayed distorted square planar geometries, with the greater steric demand of the ^*t*^Bu-wingtips resulting in a geometry index τ_4_ = 0.27 for **84** compared to τ_4_ = 0.13 for **83**. Treatment of **83** and **84** with benzyl magnesium chloride yields the corresponding alkylated complexes **85** and **86** (Scheme 13), again displaying square planar geometry in contrast to the analogous iron(II) complexes **64**–**66** and **70**–**71** with tetrahedral coordination spheres.^167^ In this instance, however, the bulkier pincer scaffold of **86** yields a more planar molecular arrangement than observed for **85**.^178^ The wingtip-effect (*vide infra*, section 4) is prominent in the follow-up hydrogenation of the alkyl complexes **85** and **86**, where the discrimination between a dinuclear hydrido complex **88** with two hydrido ligands bridging the two Cr^II^ ions and a mononuclear, square planar PNP-chromium(II) hydrido complex **87** (Scheme 13) is possible as a steric consequence of the wingtip groups. Both the di- and mononuclear chromium(II) hydrido complexes demonstrated insertion reactivity toward unsaturated C=O and C=N bonds, as anticipated for hydrido intermediates in catalytic reductions of unsaturated substrates.

### 2.3 Access to Nucleophilic T-Shaped *d*^10^ Transition Metals

Carbazolide coordination enforcing a T-shaped geometry has also been reported for the group 10^185^ and 11^186^*d*^10^ transition metals, with the amido moiety fostering a nucleophilic metal that exhibits alternative reactivity profiles. Kunz and co-workers demonstrated this for the group 10 metals,^185^ accessing nucleophilic complexes with the introduction of their BIMCA ligand consisting of a carbazole backbone substituted by two NHC (*N*-heterocyclic carbene) moieties on the 1,8-positions.^140^ The strong σ-donor properties of NHCs, stabilizing metal centers in both low and high oxidation states, are known to provide for complexes with unique reactivity and/or high catalytic activity.^71,187−189^ Modulation of the electronic consequence at the metal center by manipulation of the **L**-donor groups (Figure 1) of the **LNL**-carbazole pincer is discussed in more detail in section 3.

**Scheme 14:**
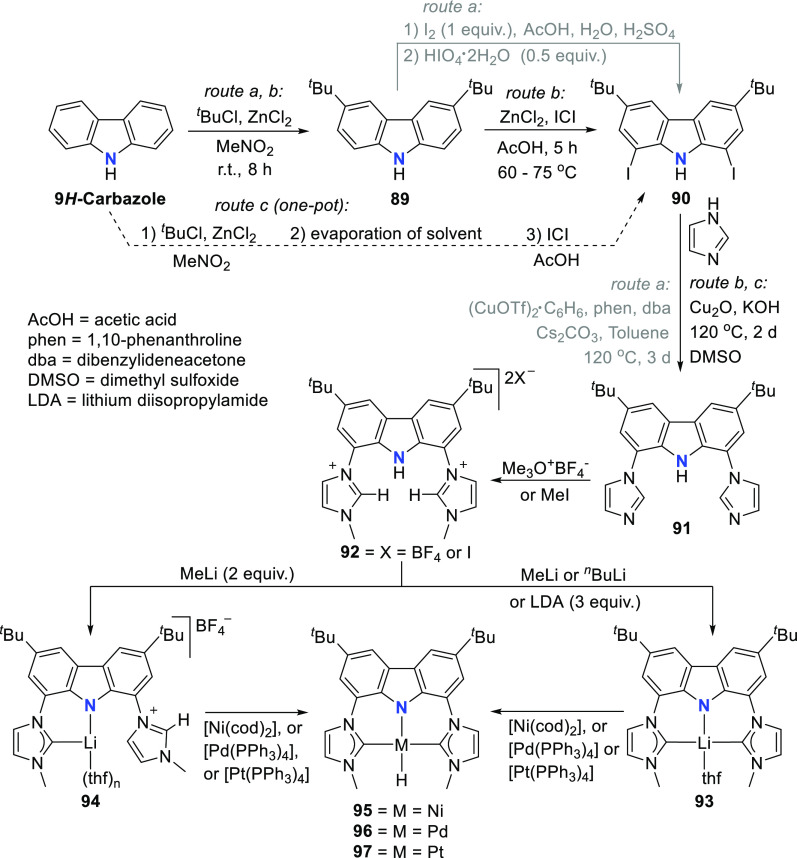
Synthesis of the BIMCA (3,6-di-*tert*-butyl-1,8-bis(imidazol-2-ylidene-1-yl)carbazolide) Ligand and Coordination to Group 10 Transition Metals

For the preparation of BIMCA ligand precursor, bis(imidazolium) salt **92**, a 4-step synthesis was first reported (*route a*, Scheme 14) starting from 9*H*-carbazole, the 3,6-positions of which was first alkylated followed by iodination of the 1,8-positions.^140^ The resulting 3,6-di-*tert*-butyl-1,8-diiodocarbazole **90** was coupled with imidazole in a copper-catalyzed Ullman reaction to give a bis(imidazolyl)-substituted carbazole **91**, which was further treated with methyl iodide or Meerwein’s salt (Me_3_O^+^BF_4_^–^) to give the protioligand, imidazolium salt **92**. Optimization of the synthesis (*route b* and *c*, Scheme 14) afforded shorter reaction times and better yields, while employing more affordable, readily available and air-stable starting materials.^190^

It was envisioned that the anionic BIMCA ligand coordinated with zerovalent group 10 metals (Ni, Pd, Pt) could provide access to very electron-rich and reactive anionic group 10 metal complexes.^185^ Transmetalation of *in situ* generated **93** with a Ni^0^, Pd^0^, or Pt^0^ precursor was attempted (Scheme 14), but the targeted anionic M^0^ complexes were not formed in the reaction. Surprisingly the M^II^-hydrido complexes **95**–**97** formed instead (Scheme 14). NMR and IR spectroscopic studies suggested that the complexes are monomeric. Further confirmation of this was provided by a single crystal X-ray structure determination of the Pd and Pt complexes **96** and **97**, respectively. The complexes have isomorphic structures in which the BIMCA ligand is meridionally coordinated to the metal center, the square planar coordination of which is completed by the hydrido ligand *trans* to the carbazolide-nitrogen. The origin of the hydrido ligand in **95**–**97** was not clear, but several options were considered as the proton source, including the BIMCA ligand itself,^185^ in contrast to the analogous group 10 metal (Ni, Pt, Pd) hydrido complexes formed unambiguously from the N–H oxidative addition of the PNP-carbazole precursor **312** (*vide infra*, section 4.1) to M^0^ precursors (M = Ni, Pt, Pd).^191^ The formation of the hydrido complexes was explained by the partial deprotonation of the bis(imidazolium) salt **92**, leading to a monodeprotonated lithiated species **94** (Scheme 14).^185^ As this intermediate reacts further with the M^0^ precursor, an anionic M^0^ complex is formed, where the BIMCA ligand is κ^2^-coordinated to the metal via the C atom of the NHC moiety and the N atom of anionic carbazole, resulting in a very basic metal complex. This facilitates the deprotonation of the uncoordinated imidazolium unit in the next step, resulting in simultaneous formation of the M^II^-hydrido complex with the formation of the carbene moiety. With this conclusion, the synthesis of the M^II^-hydrido complexes **95**–**97** was optimized, reducing the amount of base used to deprotonate **92**, to two molar equivalents, to yield a mixture of fully deprotonated and partially deprotonated **93** and **94**, respectively. Upon further reaction with the M^0^ precursors, the M^II^-hydrido complexes were isolated with improved yields (Scheme 14).

**Scheme 15:**
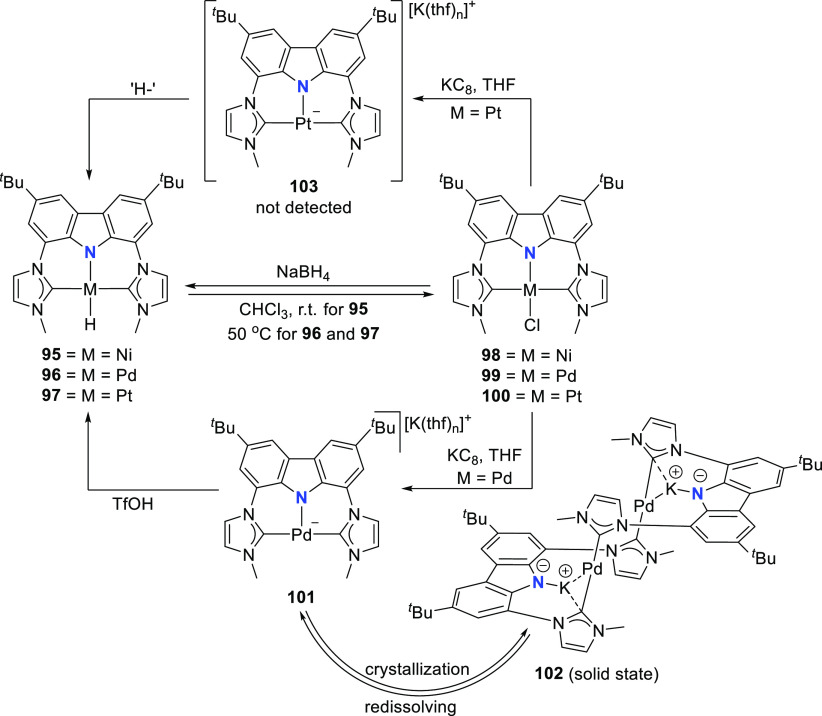
Syntheses of Group 10 Metal(II) Hydrido and Chlorido Complexes of BIMCA Ligand and Anionic M(0) Complexes Formed As a Result of the Reduction of Chlorido Complexes

Interconversion of the M^II^ hydrido complexes **95**–**97** and the corresponding stable chlorido complexes **98**–**100** was demonstrated (Scheme 15).^185^ Reduction of **100** with NaBH_4_ led to the formation of the hydrido complex **97**, whereas reduction of **99** with KC_8_ results in the formation of a dimeric Pd^0^ biscarbene complex **102**. The pincer-type coordination of the ligand collapses, and both BIMCA ligands are coordinated through their NHC moieties to one palladium(0) center while the anionic carbazolide is coordinated to potassium. In solution, the dimer **102** was observed to dissociate into the monomer **101**. On the basis of NMR DOSY experiments, it was concluded that dimer dissociation and subsequent rearrangement results in the formation of a monomeric anionic pincer palladium(0) complex **101** (Scheme 15). DFT calculations suggested formation of a solvent-separated contact-ion pair in solution. When the complex was protonated with trifluoromethanesulfonic acid (TfOH), the formation of the Pd^II^-hydrido complex **96** was observed (Scheme 15). The authors concluded that the anionic BIMCA M^0^ complexes are intermediates in the oxidative addition of **93** to the respective M^II^-hydrido complexes **95**–**97**. In contrast to palladium, for which the reactive Pd^0^-intermediate was observed both in the solid state (**102**) and in solution (**101**), the reactive platinum(0) intermediate **103** (Scheme 15) was not observed. It is probably formed *in situ* and is very basic, which allows the abstraction of a proton from the ligand of another molecule. This metal-basicity was inferred from theoretical considerations, where the antibonding HOMO of all the calculated zerovalent [M(BIMCA)]^−^ fragments are aligned along the N–M bonding axis, and the occupation of this orbital (*trans* to M–N) leads to elongated M–N bonds for the hydrido complexes **95**–**97**, compared to their calculated metal-chlorido analogues.

**Scheme 16:**
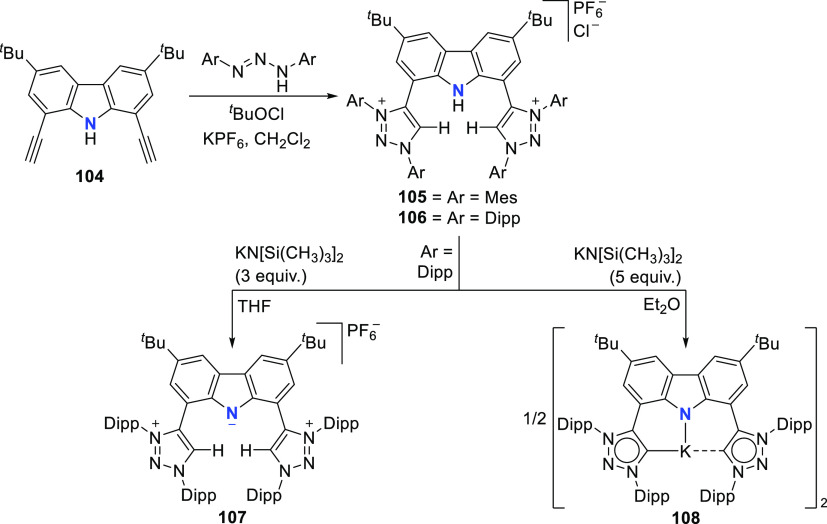
Synthesis of Dicationic Bis(triazolium)carbazole Ligand Salts

The BIMCA ligand was also coordinated to the coinage metals Cu and Au.^192^ In the case of copper, depending on the copper source used, either a paramagnetic neutral copper(II) chlorido complex or a dinuclear cationic copper(II) complex is formed. The corresponding Cu^I^ complex was not reported. However, if the identity of the flanking carbon-donor carbenes is changed, in addition to the wingtip steric bulk, access to a stable T-shaped Cu^I^ complex is granted.^193^ The monoanionic bis(triazolylidene)carbazolide analogue to the BIMCA ligand introduces mesoionic carbenes (MICs) as the C-donor ligands, with their proven strong σ-donating ability.^194,195^ The dicationic bis(triazolium)carbazole precursors **105**(193) and **106**(196) are obtained from the 1,3-dipolar cycloaddition reaction between the 1,3-diaryl-2-azoniaallene salt and 1,8-diethynylcarbazole **104** (Scheme 16). Treatment of the protioligand **106** with 3 equiv of potassium hexamethyldisilazane (KHMDS) results in a monodeprotonated cationic salt **107**.^193^ However, increasing the amount of base to 5 equiv circumvents the equilibrium between the base and its conjugated acid amine to yield a fully deprotonated compound that can be isolated as the potassium salt **108** (Scheme 16), which is stable both in solution and in the solid state under anhydrous conditions.

**Scheme 17:**
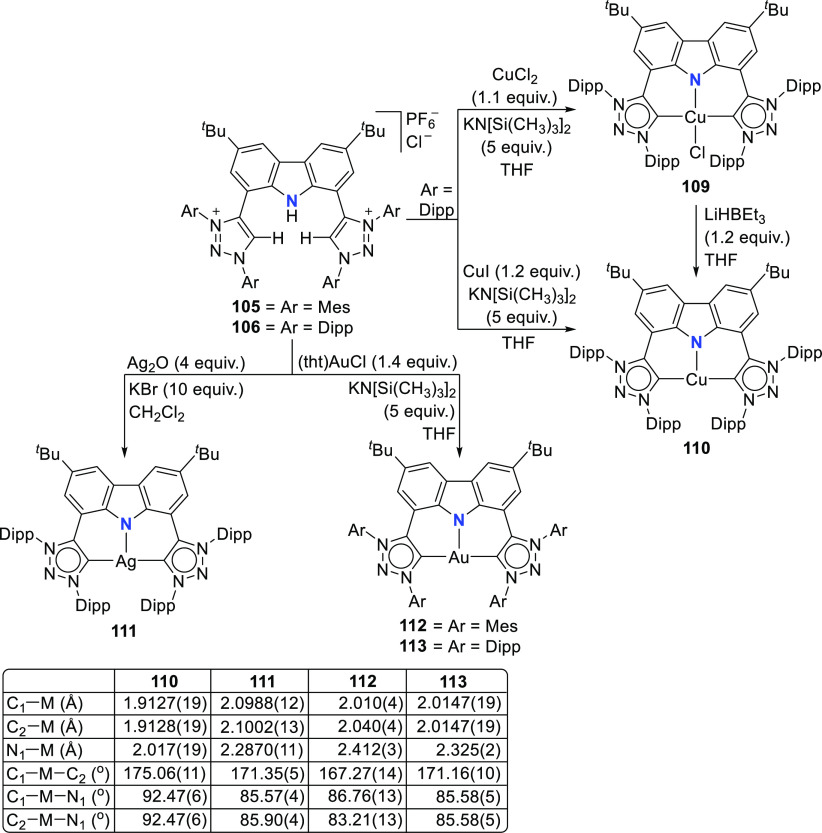
Syntheses of Coinage Metal Complexes Coordinated by the Bis(triazolylidene)carbazolide Ligand

Reaction of precursor **106** with an excess of KHMDS and copper(II) chloride produces the paramagnetic copper(II) complex **109** (Scheme 17).^193^ Treatment of **109** with superhydride (LiHBEt_3_) results in the reduction of Cu^II^ to Cu^I^ to yield the copper(I) complex **110**, which is also obtained from the reaction of **106** with CuI in the presence of KHMDS (Scheme 17). The range of uncommon T-shaped *d*^10^ coinage metal complexes could be expanded to also include Ag^I^ (**111**)^197^ and Au^I^ (**112** and **113**)^186^ as a result of the fine-tuned ligand anchoring the metal in the pincer pocket. More importantly, in the case of gold, “remote” basicity similar to the calculated [M(BIMCA)]^−^ fragments^185^ was demonstrated, rendering the gold(I) site nucleophilic, a feature arising from both the unusual, strained T-shaped geometry combined with the strong electron-donating nature of the ligand.^186^ T-shaped Au^I^ complexes **112** and **113**(186) were prepared similarly to the Cu^I^ complex **110**(193) by a reaction of *in situ* triply deprotonated protioligands **105** or **106** and the metal precursor (Scheme 17). On the other hand, the Ag^I^ complex **111**(197) was obtained by the direct metalation of the ligand precursor with an excess of Ag_2_O in the presence of KBr and in the absence of light (Scheme 17). The T-shaped geometry of the metal center in **110**–**113** was confirmed by single crystal structure determinations.^186,193,197^ In the structural analysis of the complexes, deviation from the ideal T-shape geometry is a result of the metal atom displacement above the plane defined by the ligand. This distortion is most pronounced for **112**, with 2,4,6-trimethylphenyl (mesityl = Mes) wingtips.^186^

**Scheme 18:**
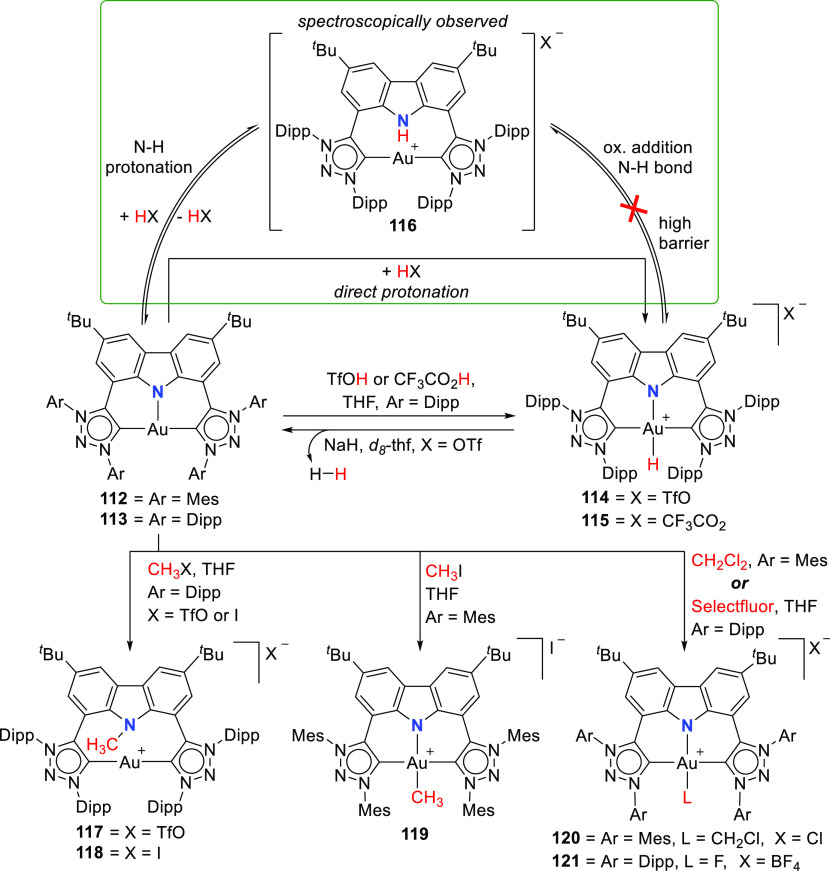
Oxidation of Au^I^ Complexes to Square Planar Cationic Au^III^ Complexes

In the treatment of the gold(I) complexes **112** and **113** with acids, e.g., TfOH, trifluoroacetic, or acetic acid, the unprecedented formation of cationic gold(III) hydride complexes **114** and **115** (Scheme 18), with near ideal square planar geometry, was observed.^186^**114** has a protic rather than hydridic character and does not react with acids. The converse reaction with NaH base leads to the reformation of **113** with simultaneous release of H_2_ gas after 3 days at room temperature (Scheme 18). Mechanistic considerations for the formation of **114** involved two possible pathways, either by direct protonation of the gold(I) center in **113** or alternatively by two steps involving the protonation of the amido nitrogen of the carbazolide, followed by oxidative addition of the N–H bond across the gold(I) center (Scheme 18). At first, the latter option seemed more promising and was supported by the NMR spectroscopic observation of the cationic gold(I) complex **116** with protonated amido N–H which was obtained from the stoichiometric reaction between **113** and TfOH. **116** was found to convert completely into Au^III^ complex **114** over an extended period of time. However, DFT calculations suggested that the oxidative addition of the N–H bond is energetically highly unfavorable, leaving the possibility of protonation occurring at both the nitrogen and the gold center as the more likely scenario. Further support for this was provided by the visualization of the HOMO of **113** showing the antibonding interaction between carbazolide-nitrogen and gold(I), resulting in a significant polarization of the corresponding occupied *d*-orbital at gold. This results in a remote metal-basicity which renders the gold(I) center nucleophilic and thus reactive toward electrophiles. This also explained the equilibrium between the cationic Au^I^ complex **116** and the neutral Au^I^ complex **113** leading to the formation of the observed gold(III) hydride complex **114**.^186^ Subsequent quantum theoretical studies suggested that the high proton affinity at the gold(I) center of the T-shaped complexes is due to relativistic effects.^198^ As a result, the electron density at the gold center increases to the extent that it is similar to, and competes with, the electron-rich amido nitrogen, thus supporting the experimental observations.

Further electrophilic oxidation of the gold(I) complexes **112** and **113** was observed when the investigations were extended to electrophilic alkylation reagents.^186^ In addition, it was found that the steric factors of the ligand affect whether the electrophilic attack targets the nucleophilic gold(I) center or the amido nitrogen of the ligand. When **113** with sterically demanding 2,6-diisopropylphenyl (Dipp) wingtip groups was treated with methyl triflate or methyl iodide, alkylation of the amido nitrogen was observed resulting in the formation of cationic linear Au^I^ complexes **117** or **118**, respectively. In contrast, the reaction of methyl iodide with **112** bearing mesityl wingtips led to the alkylation of the gold(I) followed by oxidation of the metal to give the cationic Au^III^-Me complex **119**. The formation of the cationic Au^III^-CH_2_Cl complex **120** was observed in the reaction of **112** with dichloromethane, whereas no reaction was observed with **113**. The reaction of **113** with Selectfluor afforded **121**, representing the first stable well-defined cationic gold(III) fluoride complex.^197^ The different reactivity presented by the Mes **112** and the Dipp analogue **113** may also partly be due to the different geometries of the gold(I) centers of the complexes, of which the reactive metal center in the mesityl analogue **112** is more distorted from the T-shape. This observation is in accordance with facile oxidative addition to Au^I^ made possible by chelate-assisted ligand reactivity reported by Bourissou,^199,200^ or the use of reactive partners such as a strained biphenylene,^201,202^ where increased distortion raises the energy of the complex and preorganizes it for oxidative addition.

### 2.4 Extending the Reactivity of the Carbazolide-Nitrogen Lone Pair

**Figure 2:**
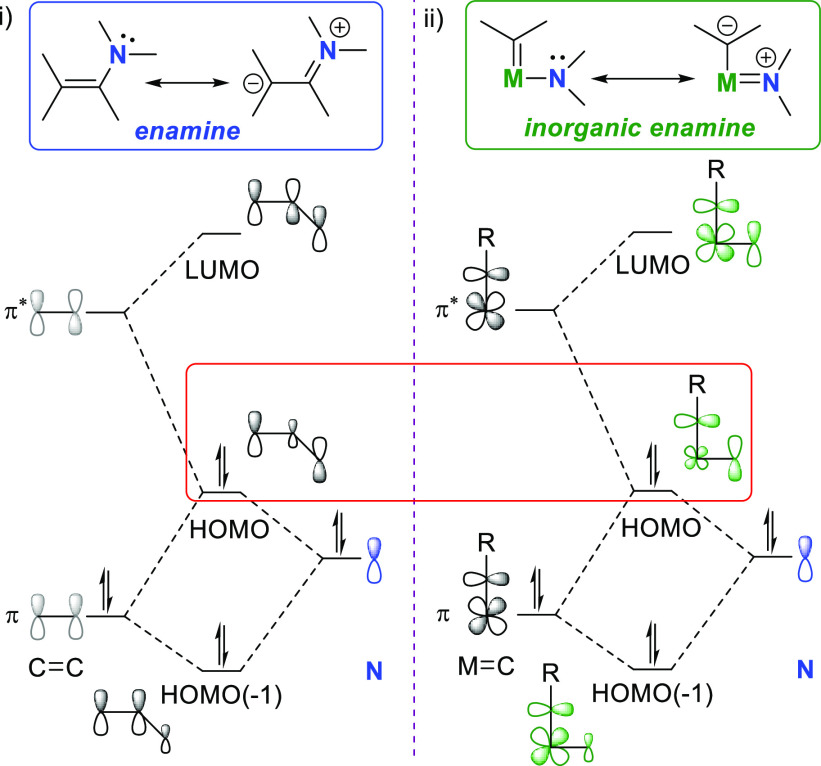
Resonance contributions and truncated qualitative orbital diagram of the bonding analogy between (i) enamines and (ii) amidoalkylidenes as analogous inorganic enamines.^203,204^

A pronounced electronic consequence of the carbazole-amido as central coordinating moiety of carbazole-based pincer ligands, is the so-called inorganic enamine effect^203,204^ observed in the bonding analysis of a trianionic ONO^3–^ pincer alkylidyne complex of tungsten.^205^ The inorganic enamine effect is the term used to describe the elevated nucleophilicity of metal–carbon multiple bonds by constraining a nitrogen atom lone pair to be collinear with a metal-carbon multiple bond to allow for orbital overlap of the bonding and antibonding combination of the N lone pair with the M–C π-bond. Figure 2 depicts the relationship between organic enamines (i, Figure 2)^206^ and inorganic enamines. The inorganic enamine interaction has as consequence the destabilization of the HOMO with π* character, while the electron density from the amido lone pair is delocalized onto the α-carbon of the metal-carbon multiple bond (ii, Figure 2) to form highly nucleophilic metal alkylidenes/alkylidynes.^205^

**Figure 3:**
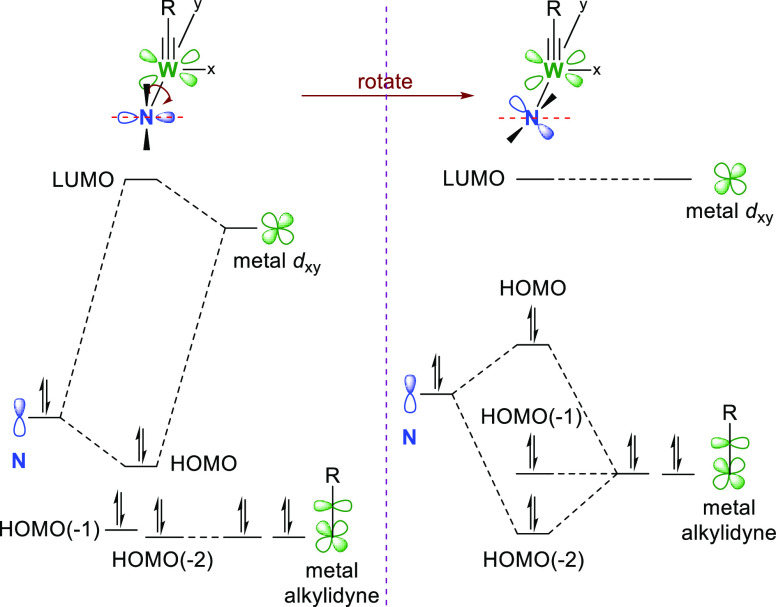
Orbital overlap for amido *p*-orbital aligned with *d*_*xy*_ (left) and amido *p*-orbital rotated out of alignment, with corresponding truncated molecular orbital diagrams of preferential overlap of freely rotating N atom lone pair with unoccupied *d*_*xy*_ orbital (left), and constrained N atom forced to overlap with one of the W≡C π-bonds (right).^203,204^

**Scheme 19:**
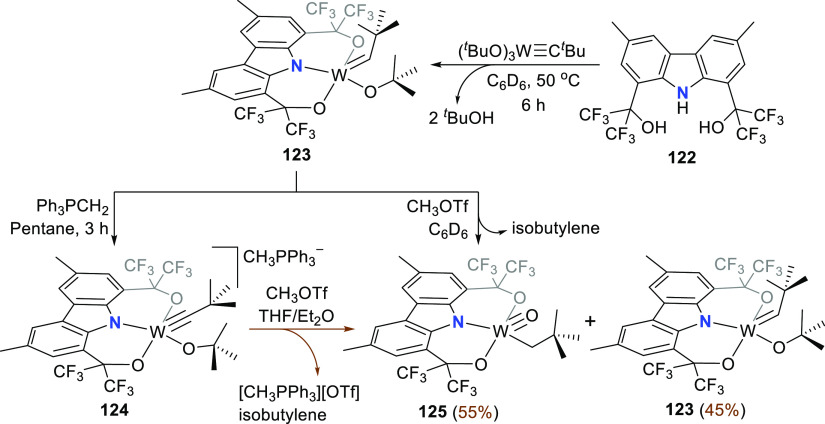
Synthesis of Alkylidene, Alkylidyne, and Oxo-alkyl Complexes of Tungsten Coordinated to a Trianionic ONO-Carbazolide Pincer Ligand

In their previous work, Veige et al. described alkylidyne tungsten complexes coordinated to a tridentate ONO-pincer ligand featuring a central amido flanked by two alkoxo-functionalized phenylene moieties.^203,204^ It was shown that the flanking biaryl moieties adjacent to the central amido rotate to create an inherent twist across the pincer backbone. Because the angle of the nitrogen atom *p* orbital approach and its energy match with the M–C π-bond naturally influences the magnitude of the orbital overlap, their target was to constrain the amido lone pair to be perfectly collinear with the metal-carbon multiple bonds to maximize orbital alignment for inorganic enamine orbital interaction (Figure 3). To accomplish this goal, a ligand precursor **122** representing a scaffold with the two aryl moieties of the previously reported ONO-pincer ligand was prepared from dibromo-precursor **18** (Scheme 19).^205^

Metalation of **122** with (^*t*^BuO)_3_W≡C^*t*^Bu yielded the alkylidene complex **123** with near-perfect square-pyramidal geometry (τ_5_^207^ = 0.059) (Scheme 19). The ONO^3–^ trianionic pincer and the *tert*-butoxide ligand reside on the basal plane, with the planar carbazole forcing the N-aryl rings in a coplanar arrangement, while the alkylidene fragment occupies the axial position as determined from the molecular structure.^205^ The trigonal plane of the nitrogen atom is oriented perpendicular to the W=C bond axis, as designed. Treatment of **123** with Ph_3_PCH_2_ deprotonates the alkylidene and precipitates the anionic alkylidyne complex **124**. Treatment of **124** with MeOTf did not result in the expected formation of a neutral metal alkylidyne derivative. Instead, complex **123** is regenerated alongside the oxo-alkyl complex **125** (Scheme 19). Formation of **123** was ascribed to the presence of adventitious protons, while formation of **125** from **123** was confirmed in an independent reaction with MeOTf and expulsion of isobutylene.

**Figure 4:**
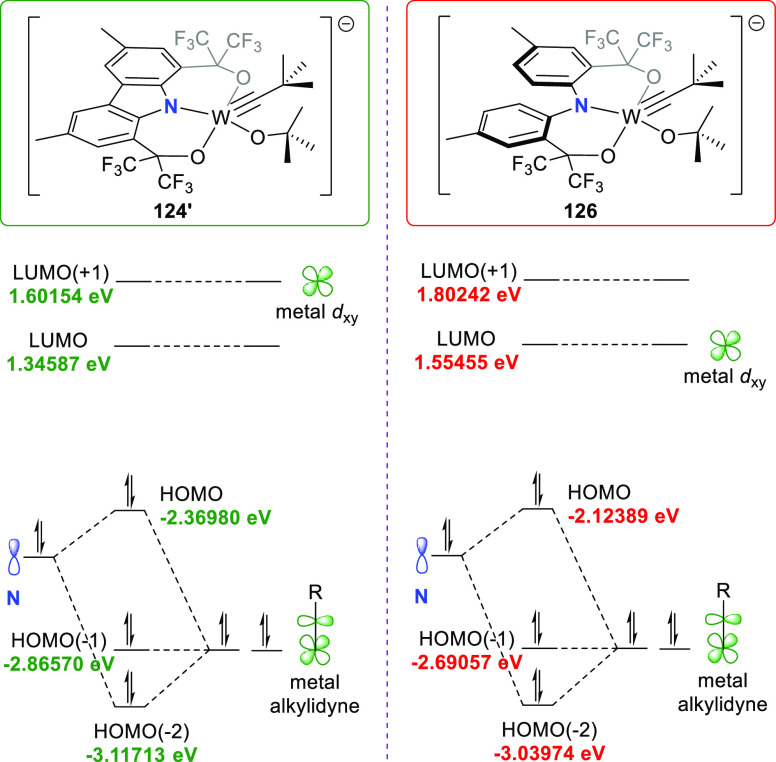
Truncated molecular orbital diagrams exhibiting inorganic enamine bonding combinations for the anions **124′** and **126**.^205,208^

DFT calculations were performed to examine the electronic structure of the rigid anion of **124** (Figure 4, left) and to compare its orbital overlap with the previously reported, flexible analogue **126** (Figure 4, right).^208^ From the computed structures **124′** and **126**, it was evident that the nitrogen atom lone pair is nearly collinear with the alkylidyne π-orbitals in both complexes to generate an inorganic enamine between the HOMO(−2) and the HOMO with an overlap of similar magnitude (Figure 3 and Figure 4, right).^205^ The rigid ligand was expected to have a significantly stronger overlap; however, several factors other than the *p* orbital orientation influences the magnitude of the overlap. As an example, a distinct difference was noted in the magnitude of the electron density on the nitrogen atom within the molecular orbital that overlap with the π-W–C bond for **124′** and **126**. In **124′**, the electron density is delocalized over the carbazole backbone, while it is largely N-centered in the twisted ligand of **126** with a more basic amido moiety. This can be further supported with the p*K*_a_ values (Scheme 7). Thus, despite the prominent structural differences between **124** and **126** and achieving the restriction of the *p* orbital on the nitrogen atom for collinearity with the W–C π-bond and inorganic enamine reactivity, these factors alone are not sufficient to accomplish increased orbital overlap and influencing their relative energies. More generally, though, is the observation throughout this review that increased delocalization into the carbazole backbone ensures increased aromaticity across the backbone over the metal carbon bond, which certainly becomes important during redox and photocatalyzed processes, *vide infra* section 5.

**Scheme 20:**
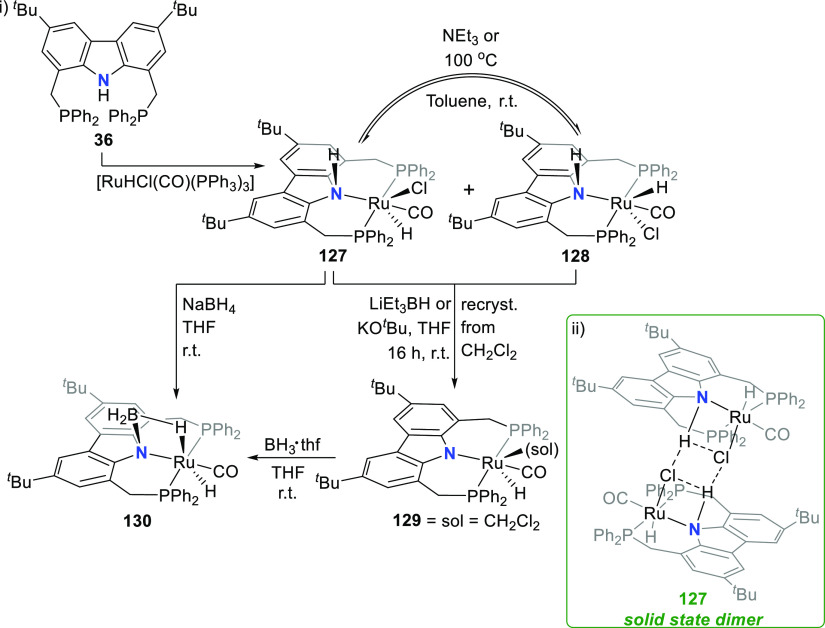
Synthesis of Hydrido Ruthenium(II) PNP-Pincer Complexes and Cooperative Reactivity with Borane

Another example implicating the carbazolide-nitrogen’s lone pair in directing complex reactivity is found in the observed metal-ligand cooperativity^209^ of a PNP-pincer complex of ruthenium.^210^ B–H activation was reported from the reaction of the borane-thf adduct with either a neutral protonated or an anionic PNP-pincer complex of ruthenium. The protonated complex **127** was prepared from the reaction of protioligand **36** with [RuHCl(CO)(PPh_3_)_3_] to form the two stereoisomeric hydrido complexes **127** and **128** (i, Scheme 20). The 1,2-dehydrochlorination reaction products contain both a metal-bound hydrido ligand as well as a protic NH moiety at the carbazole backbone of the pincer. The molecular structure of the isolated stereoisomer **127** displays an intermolecular hydrogen-bonding interaction between the chloride ligand and the carbazole-NH to form a dimeric structural arrangement around the central H_2_Cl_2_ cycle (ii, Scheme 20). If **127** is heated at 100 °C or treated with triethylamine, conversion to the stereoisomer **128** is observed, but **128** reverts to the steady-state equilibrium favoring **127** at room temperature (i, Scheme 20). Both **127** and **128** react with a strong base (KO^*t*^Bu or LiEt_3_BH) to yield deprotonated **129** with an anionic carbazolide scaffold with significantly shorter Ru–N bond length (2.301 Å for **127** compared to 2.173 Å for **129**), with weak solvent coordination completing the octahedral coordination sphere around the ruthenium metal. Reaction of BH_3_·thf with **129** at room temperature leads to the 1,2-addition of the BH_3_ moiety to the Ru–N functionality to form a RuNBH cycle in **130** (i, Scheme 20).^210^ The borane-bridged **130** can also be formed by reaction of **127** with sodium borohydride in solvent THF. Structural analysis of the X-ray diffraction data shows the ruthenium-bound H-atoms in the axial positions of the trigonal bipyramidal coordination geometry. The Ru–H bond that forms part of the RuNBH cycle is elongated (1.80 Å) compared to the other Ru–H (1.58 Å), and while the Ru···B distance (2.458(2) Å) is longer than the sum of the covalent radii (2.09 Å), it is shorter than those of ^1^η-B–H σ-type complexes.^211^ The authors surmised that the Ru···B distance reflects the geometric demands of the coordination mode of the BH_3_ unit to the Ru–N function in the complex rather than additional attractive interactions between the ruthenium and boron centers,^210^ with a B–N single bond (1.58(2) Å) instead of a B=N bond with π-character as expected for interaction with a quaternary nitrogen.^212^

## 3 Flanking Donor Effects

The effects of the donor moieties flanking the central carbazole moiety can be differentiated into effects governed primarily by the tethering **E**-group (connecting donor **L** and 1,8-carbazole positions, Figure 1) in section 3.1, the identity of the ligating **L**-group (in this review, only neutral C-, N-, and P-donors are reported, with singular examples of anionic O-donors) in section 3.2, or a combined effect of the **EL**-donors in section 3.3 leading to metal-ligand cooperativity.

### 3.1 Controlling the Size of the Lanthanide Chelate

When considering the effect of **E** in the **L(E)N(E)L**-carbazolide scaffold (Figure 1), the presence or absence of an **E**-moiety would dictate the formation of 5- or 6-membered metallacycles of the three “pincing” moieties. In turn, 5- versus 6-membered chelation could prevent or allow for intramolecular C–H activation of the **E** aliphatic tethers linking the 1,8-positions of carbazole with donor ligand moieties **L**, respectively. The 5-membered chelation leads to “open” chelate (less acute ligand bite angle), decreasing the propensity to undergo cyclometalation. On the other hand, a 6-membered chelate positions wingtip substituents in close proximity and correct geometry to undergo cyclometalative reactions (noted for carbazole and pyrrole backbones; see section 3.3 below).^213^ In this context, carbazole-based pincers provide an excellent alternative to the cyclopentadienyl ligands and their derivatives that are often employed in lanthanide organometallic chemistry and catalysis. Despite the frequent application of cyclopentadienyl ligands in this area, their steric and electronic modulation are limited, especially when likened against tridentate pincer ligands. Furthermore, multidentate coordination can impart additional stabilization to lanthanides against decomposition and ligand redistribution reactions, a drawback that has hindered the progress made with regard to lanthanide organometallics, especially when compared against the transition metals. Carbazole pincers address both concerns, with tridentate coordination increasing the stability of the complex in addition to facile electronic and steric fine-tuning leading to refined complex/catalyst.

#### 3.1.1 5-Membered Chelation

**Scheme 21:**
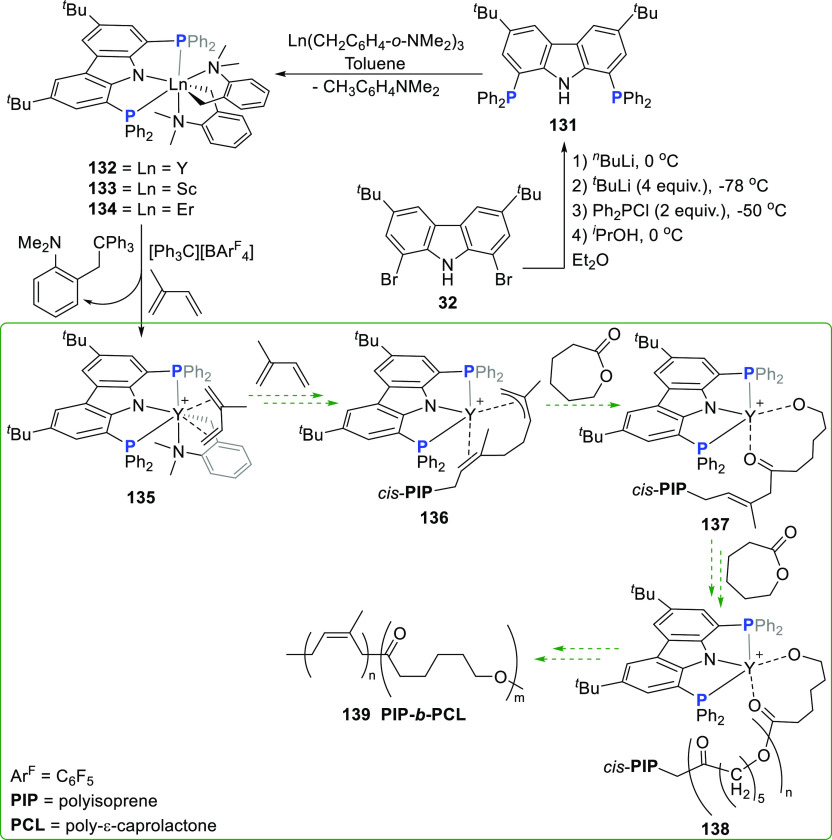
Synthesis of 5-Membered Chelating PNP-Pincer Lanthanide Complexes for Polymerization of Dienes

A carbazole pincer ligand containing phosphino-donor groups directly bonded to the carbazole 1,8-positions was employed by the group of Cui in the synthesis of well-defined rare earth metal pincer complexes containing 5-membered chelated metallacycles.^214^ The dibrominated carbazole precursor **32** was sequentially lithiated, whereafter metathesis with PPh_2_Cl yielded the protioligand precursor **131** (Scheme 21). The combination of the large, soft phosphorus donor and hard, anchoring carbazole-amido donor was anticipated to stabilize the dialkyl rare earth metal complexes, while the 5-membered chelation was specifically employed to preclude unpredictable C–H activation of the ligand backbone. Simultaneously, the tridentate coordination would circumvent the usual challenges of dimerization and ligand scrambling for the rare earth metals, even in a complex with relatively low steric crowding. Deprotonation and complexation of **131** were achieved by reaction with the rare earth metal tris(*o*-aminobenzyl) derivatives to yield the yttrium(III) (**132**), scandium(III) (**133**), and erbium(III) (**134**) complexes (Scheme 21). A striking structural feature observed in the crystal structure of seven-coordinate **132** is the large ligand bite angle [bond angle P–Y–P = 118.06(3)°] compared to the bite angles of 6-membered chelating PNP-carbazolide metal complexes (**259**, **263**, and **266**, *vide infra*, Scheme 40) with coordination number seven, where P–M–P bond angles can range between 89.9–101.6°, albeit that these contrasting examples feature an early transition metal with PN(C)P coordination mode of the carbazole-based pincer.^173^ NMR spectroscopic studies, as well as the solid state structure obtained for **132**, confirmed the absence of solvent coordination, while both aminobenzyl ligands were found to coordinate as chelating bidentate ligands to yield the solvent-free seven coordinate monomer with the Y^III^ deviating slightly from the planar pincer geometry as a result of the steric congestion of the diphenylphoshpine groups.^214^ In contrast, a related PNP-pincer dialkyl yttrium complex derived from flexible bis(diphenylphosphino) amido ligand displays thf coordination, with ligand P–M–P bite angles of 102.7–106.6°.^215^

**Scheme 22:**
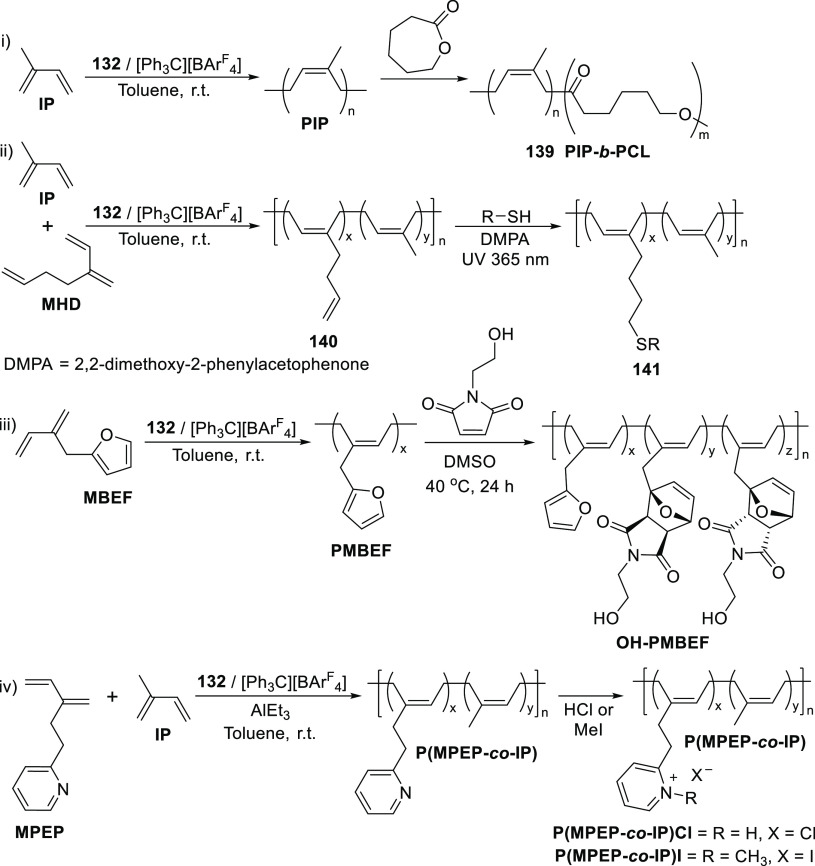
*Cis*-1,4-Selective Living Diene Polymerization and Subsequent Post-Modification

Addressing the need for new catalyst systems beyond that of the Ziegler–Natta type for the polymerization of 1,3-conjugated dienes requires catalysts that exhibit a living mode as well as high *cis*-1,4-selectivity to control the rheological and mechanical properties of these important rubbers.^216,217^ Cui et al. demonstrated that complexes **132** and **133** are highly active (complete conversion within 10 min) toward isoprene (IP) polymerization with borate activation instead of activation with a trialkylaluminum activator, whereas the erbium catalyst **134** was slightly less active (30 min conversion time).^214^ Not only was excellent *cis*-1,4-regularity obtained (>99%), but regularity was maintained at polymerization temperatures up to 80 °C. More remarkably, a living mode was exhibited for the **132** or **134** and [Ph_3_C][BAr^F^_4_] (Ar^F^ = C_6_F_5_) catalyst systems, with molecular weights of the polyisoprene (PIP) increasing linearly with monomer to initiator ratio increase. Encouraged by these results, block copolymerization of IP and ε-caprolactone (ε-CL) was carried out to yield a 100% conversion to the PIP*-b*-PCL block copolymer **139** with designable molecular weight (i, Scheme 22). Abstraction of the alkyl moiety *o*-CH_2_C_6_H_4_NMe_2_ with a trityl cation [Ph_3_C][BAr^F^_4_] is required to yield the cationic monoalkyl complex **135** with release of the coupling product Ph_3_CCH_2_C_6_H_4_NMe_2_-*o* (Scheme 21). NMR studies confirmed *cis*-η^4^ coordination of the IP monomer to the cationic **135**, inserted into the Y–CH_2_ bond, and propagated to cationic yttrium polyisoprene (PIP) **136** as the active species. **136** initiates the polymerization of CL on the carbonyl carbon via cleavage of an acyl-oxygen bond (**137**), leading to the formation of copolymer **139** via **138**. Notably, although the yttrium precursor Y(CH_2_C_6_H_4_-*o*-NMe_2_)_3_ is active in the polymerization, it is nonselective and not living, and the selectivity and activity in the dual catalysis of diene polymerization and ring-opening of polar ε-CL are therefore attributed to the introduction of the PNP-carbazolide ligand.

Expanding the catalytic polymerization repertoire of **132** to include (co)polymerization of 3-methylenehepta-1,6-diene (MHD) demonstrated that the high stereotacticity (*cis*-1,4-selectivity up to 98.5%) is maintained in the homopolymer PMHD and the copolymer **140** obtained with pendant vinyl groups ranging from 10–90% (ii, Scheme 22).^218^ Moreover, postmodification of the vinyl groups in every chain unit could be quantitatively effected. Conversion of the vinyl groups into a variety of thiol functionalities via a light-mediated thiol-ene reaction with photoinitiator DMPA (2,2-dimethoxy-2-phenylacetophenone) yields functionalized polybutadiene materials **141** with enhanced hydrophilicity. The copolymerization of polar and nonpolar monomers with subsequent functionalization of the polymers allows for modification or improvement of material properties. In this regard, living, *cis*-1,4-selective polymerization of 2-(2-methylidenebut-3-enyl)furan (MBEF) could be similarly achieved upon borate activation (iii, Scheme 22), without the need to mask the polar furan groups in the formation of PMBEF.^219^ Functional rubber materials are accessible after Diels–Alder addition of the furan groups in PMBEF with 1-(2-hydroxyethyl)-1*H*-pyrrole-2,5-dione to yield hydroxyl-functionalized polymers OH-PMBEF with 75% conversion containing a mixture of both *endo* and *exo* diastereomers. In contrast, copolymerization of IP with polar 2-(3-methylidenepent-4-en-1-yl)pyridine (MPEP) does require equimolar addition of triethylaluminum to form an adduct with the pyridine prior to polymerization (iv, Scheme 22).^220^ Nevertheless, copolymerization of MPEP and IP proceeds to yield a copolymer P(MPEP-*co*-IP) containing up to 17 mol % of the polar MPEP, compared to the commonly reported 10 mol % incorporation for other catalyst systems.^221^ Complete quaternization of the pendant pyridine nitrogen groups can be effected by treatment with HCl at room temperature to yield P(MPEP-*co*-IP)Cl while alkylation with methyl iodide yielded P(MPEP-*co*-IP)I.^220^ A self-standing and elastic film could be prepared from P(MPEP-*co*-IP)I as a result of cationic pyridine aggregation to form pseudo cross-link joints.

#### 3.1.2 6-Membered Chelation

**Scheme 23:**
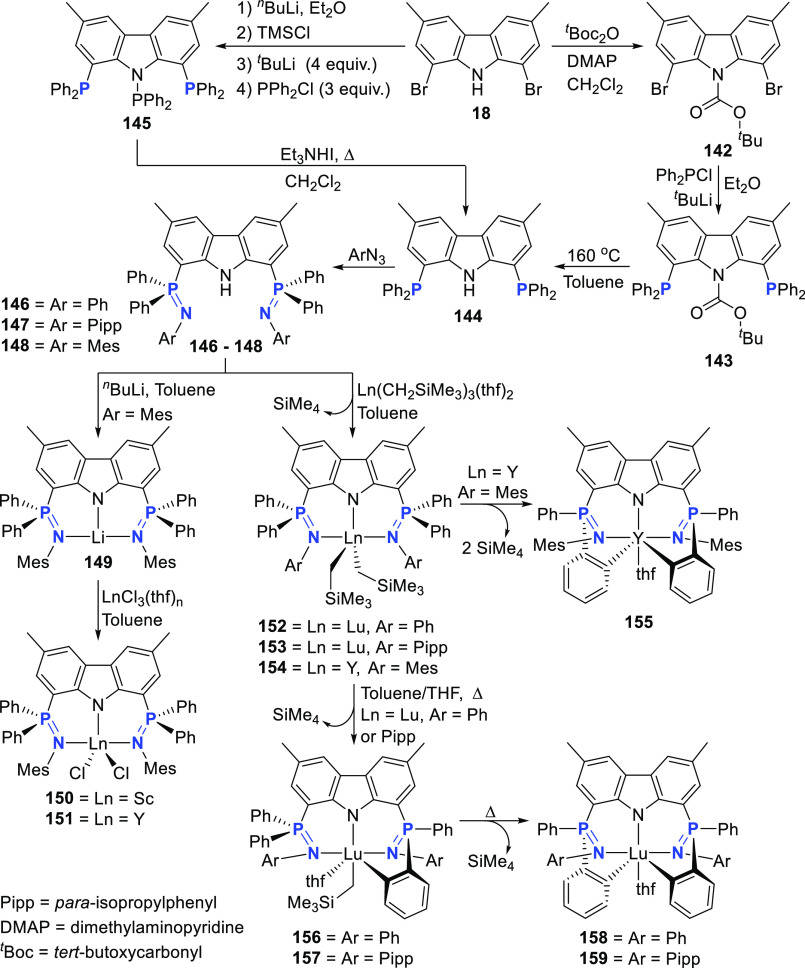
Synthesis and Reactivity of Pincer Coordinated Lanthanide Complexes

Cyclometalation reactivity of the **E**-linkers between carbazole and **L**-donors in the pincer ligand is not always well-controlled, but the reactivity can be harnessed for lanthanides or excluded by changing the nature of the **E**-linkers. The group of Hayes explored the chemistry of 6-membered chelated organolanthanides coordinated by a tridentate carbazolide.^222^ The authors reported the synthesis of a novel bis(phosphinimine)carbazole ligand, attributed to have appreciable electron-donating character, with easy substitution of the groups at both the phosphorus and nitrogen atoms, further increasing the handle on catalyst fine-tuning. Accordingly, the 1,8-diphosphine **144** was accessed from the dibromo **142** through phosphine substitution followed by deprotection of the carbazole-nitrogen (Scheme 23). The bis(phosphinimine) ligands **146** and **147** could be isolated from **144** and the corresponding azide under standard Staudinger reaction conditions. Alternatively, protioligand **148** was accessed from **144**, in turn obtained from the deprotection of **145**.^223^ An alkane elimination reaction between Lu(CH_2_SiMe_3_)_3_(THF)_2_ and protioligands **146** and **147** yielded the bis(phosphinimine)carbazolide lutetium complexes **152** and **153**, respectively (Scheme 23).^222^ The complexes could only be characterized by solution NMR spectroscopic techniques below 0 °C, as they were determined to be thermally unstable preventing their isolation. Above 0 °C, metal-ligand reactivity results in thermal decomposition with two consecutive intramolecular phenyl C–H activation and metalation reactions, yielding the cyclometalated Lu complexes **158** and **159**. A σ-bond metathesis was reported as the reaction pathway through which the phenyl ortho C–H bond reacts. Similarly, yttrium complexes could also be prepared from the bis(phosphinimine)carbazolide featuring increased steric bulk at the donor wingtip sites.^223^ Yttrium complex **155** was isolated after cyclometalation of **154** coordinated by ligand **148**. Yttrium and scandium dichloro-complexes **150** and **151**, respectively, could also be synthesized, determined to be stable even when heated up to 140 °C. The stability was attributed to the increased steric bulk (Scheme 23).

**Scheme 24:**
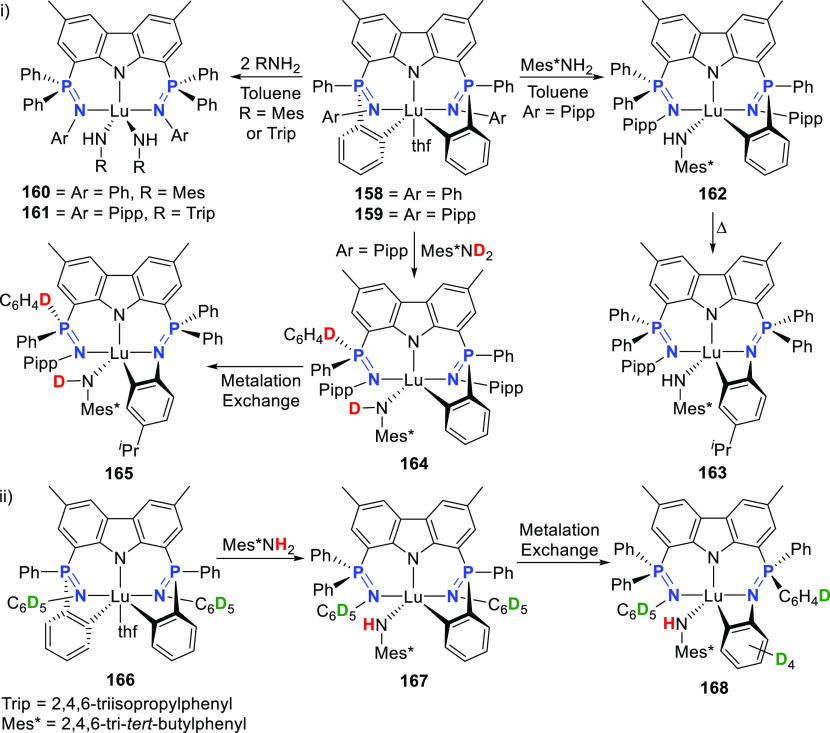
Metallacycle Ring Opening and *ortho*-Metalation Leading to Lutetium Anilide Formation

The reactivity of **158** and **159** (Scheme 23) was further investigated, exploiting the basic character of the phenyl carbon–metal bond.^224^ Specifically, **158** was reacted with two equivalents of 2,4,6-trimethylaniline (MesNH_2_), which yielded the corresponding bis(anilide) **160** (i, Scheme 24). Ligand-assisted reactivity facilitates N–H bond activation across the metal-phenyl bond, followed by metallacycle ring-opening and metal-anilide bond formation. Bis(anilide) formation was reported even when reacting **158** with only one equivalent of MesNH_2_. Similar reactivity was also noted when reacting the more sterically encumbered **159** with 2,4,6-triisopropylaniline (TripNH_2_), isolating the corresponding bis(anilide) **161** (i, Scheme 24). In contrast to the repeated double N–H bond activations observed for **158** and **159** (i, Scheme 24), mono(anilide) formation could be enforced when reacting the six coordinated lutetium with an aniline that has even greater steric bulk compared to MesNH_2_ or TripNH_2_. Thus, when reacting **159** with 2,4,6-tri-*tert*-butylaniline (Mes*NH_2_) in toluene, mono(anilide) **162** formed. Again, complex **162** could not be isolated but was characterized via NMR spectroscopy. The corresponding bis(anilide) was not formed, even in the presence of excess aniline or at a reaction temperature of 100 °C maintained for 24 h. Interestingly, the mono(anilide) **162** was found to be susceptible towards a thermally induced intramolecular rearrangement reaction, leading to **163**.

Deuteration experiments allowed insight into the reaction mechanism toward **163** (i, Scheme 24).^224^ Reacting **159** with Mes*ND_2_ yielded **165** featuring a deuterium at the anilide nitrogen. This suggested initial N–D activation with metallacycle ring-opening leading to the observed **164**, followed by direct metalation exchange between the two aryl substituents at the phosphinimine donor arm, namely the exchange of the phenyl group for the 4-isopropylphenyl substituent, resulting in **165**. A second deuterium labeled experiment corroborated the results, with metallacycle ring-opening followed by direct metalation exchange of the aryl rings. Hence, reacting the deuterium-labeled **166** with Mes*NH_2_ yielded **168** via **167**, with the proton still bound to the anilide nitrogen (ii, Scheme 24). Deuterium exchange between the two aryl substituents involved in the direct metalation exchange reaction was confirmed, further supporting the proposed reaction mechanism.

**Scheme 25:**
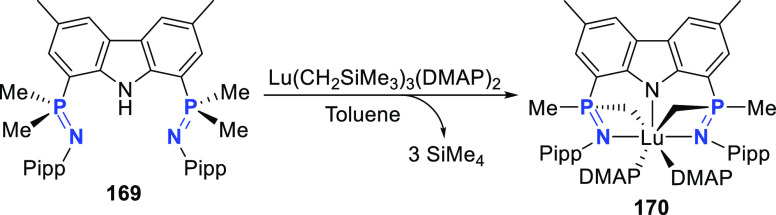
Decreasing the Steric Bulk at the P-Site of the Donor Groups

Decreasing the steric bulk at the P-site of the donor arm to methyl substituents also leads to C–H activation and cyclometalation.^225^ Ligand **169**, with decreased steric bulk at the **E**-linker groups, was reacted with Lu(CH_2_SiMe_3_)_3_(thf)_2_ similar to previous routes toward the organolanthanides. However, complex isolation was not possible and neither was spectroscopic characterization as a result of extreme thermal instability. Stabilization of the targeted complex was brought about by substituting the lutetium precursor’s thf coligands with 4-dimethylaminopyridine (DMAP). As a result, coordination of ligand **169** to Lu(CH_2_SiMe_3_)_3_(DMAP)_2_ yielded the doubly cyclometalated Lu complex **170**, with two methyl C–H activation and cyclometalation reactions leading to formation of the distorted pentagonal bipyramidal complex (Scheme 25). The complex was determined to be unreactive to anilines.

**Scheme 26:**
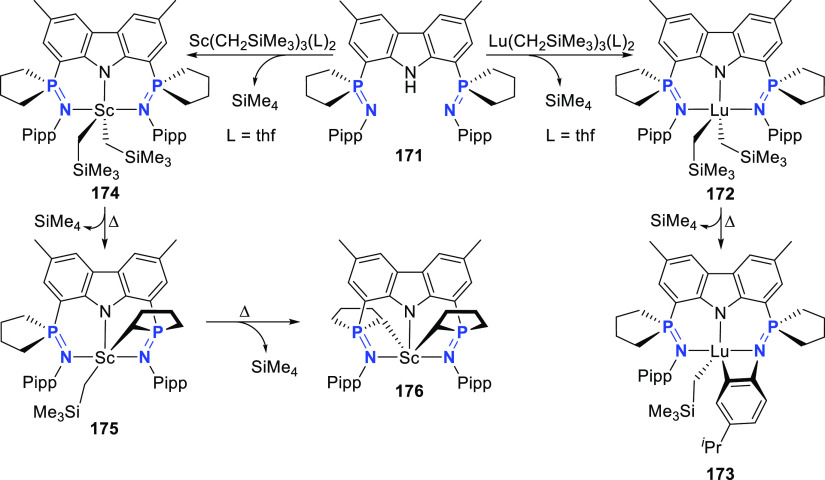
Coordination of Bis(phospholane)carbazolide to Lutetium and Scandium

At the same instance of decreasing steric bulk at the **E**-site of the P-donor group (**169**, Scheme 25), Hayes et al. also introduced a cyclic phospholane at the phosphorus moieties.^225^ The corresponding bis(phospholane)carbazole ligand **171** was prepared and coordinated to lutetium and scandium (Scheme 26). Reacting ligand **171** with Lu(CH_2_SiMe_3_)_3_(thf)_2_ yielded **172** through an alkane elimination reaction. Complex **172** could only be characterized *in situ* with NMR spectroscopic methods, due to thermal instability leading to cyclometalation and formation of **173**, followed by complex decomposition (Scheme 26). Noteworthy is the different result obtained upon treatment of ligand **171** with Sc(CH_2_SiMe_3_)_3_(thf)_2_, which yielded **176** through two consecutive cyclometalation reactions at the phospholane’s α-position, as determined by NMR analysis.

**Scheme 27:**
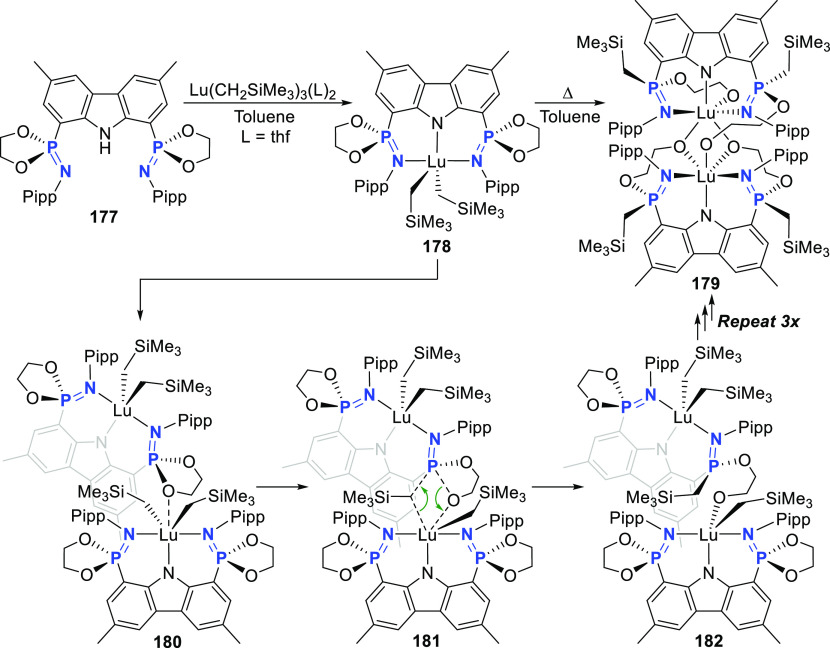
Asymmetric Dinuclear Tetraalkoxide Lutetium Complex via a Cascade Ring-Opening Insertion Reaction

A variation of the phospholane donor group was also reported by Hayes and co-workers, introducing an oxygen at the phospholane’s α-position, which markedly changed the reactivity outcome.^226^ An alkane elimination reaction led to formation of the lutetium complex **178** from ligand **177** (Scheme 27), similar to what was described above. Again, it was disclosed that the bis(alkyl) complex **178** reacted further at higher temperatures. Unlike the phospholane **172**, **178** reacts via a ring-opening insertion reaction of the dioxaphospholane donor group to yield the asymmetric dinuclear tetraalkoxide complex **179** (Scheme 27). A proposed mechanism is depicted in Scheme 27. The oxygen of a dioxaphospholane of **178** coordinates to a second lutetium metal center at **180**, with concomitant ring-opening insertion with a four-centered transition state as the intermediary species (**181**, Scheme 27). From the four-centered transition state ensues the cleavage of the oxygen–phosphorus bond and formation of a new oxygen–lutetium bond at **182**. The process repeats several times, ultimately leading to **179**.

**Scheme 28:**
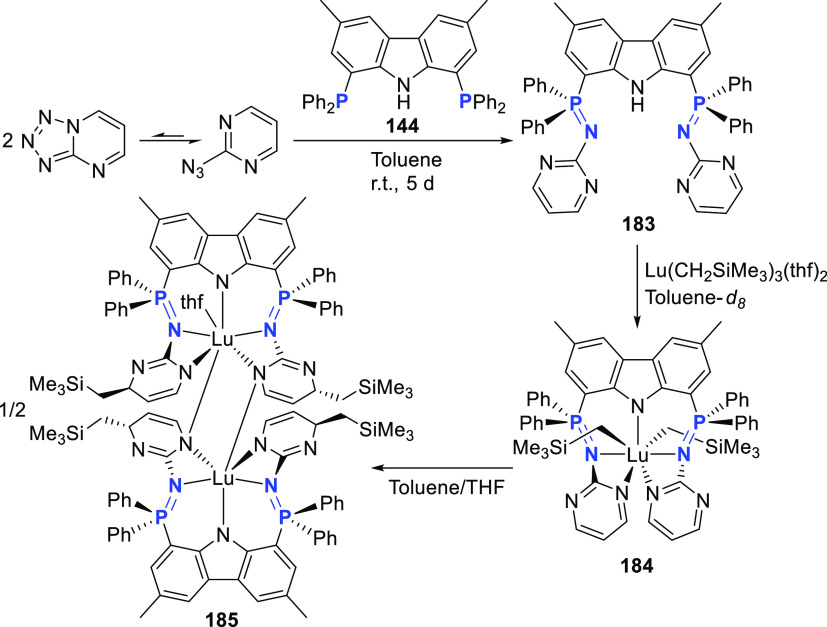
Alkyl Lutetium Leading to Dearomatization and Complex Dimerization

Varying the substituents at **L** (see Figure 1) from a bulky moiety to a pyrimidine functionality, changed the intramolecular reactivity of the formed lanthanide complex.^227^ It was reasoned that the lack of wingtip ortho C–H bonds, in addition to the potential denticity of the ligand being increased by introduction of more nitrogens in the ligand, would hamper the cyclometalative pathway encountered for previously reported bis(phosphinimine)carbazole ligands, which could lead to alternative reactivity. Ligand **183**, prepared from **144** and 2-azidopyrimidine under Staudinger reaction conditions, was coordinated to Lu(CH_2_SiMe_3_)_3_(thf)_2_ yielding seven-coordinate complex **184** which was characterized *in situ* by NMR spectroscopy (Scheme 28). Complex **184** demonstrated increased thermal stability, with a half-life of more than 5 h compared to other bis(phosphinimine)carbazolide coordinated lutetium dialkyl species with half-lives of less than one hour. The increased thermal stability was ascribed to the coordination of two additional nitrogens from the pyrimidine wingtip groups. Furthermore, **184** does not undergo intramolecular cyclometalation as described above. After several hours in solution, **184** converted to the dinuclear **185**, a reaction accelerated at elevated temperatures. Interestingly, alkyl migration gave rise to pyrimidine dearomatization, with the ensuing complex featuring an anionic nitrogen coordinating to one metal, while the same nitrogen coordinates as a neutral Lewis base to the second metal, evidenced by crystal structure analysis of **185**.^227^ The authors reasoned that a 1,3-alkyl migration in **184** with subsequent isomerization, followed by a second 1,3-alkyl migration and isomerization, and finally dimerization, leads to **185** (Scheme 28).

**Scheme 29:**
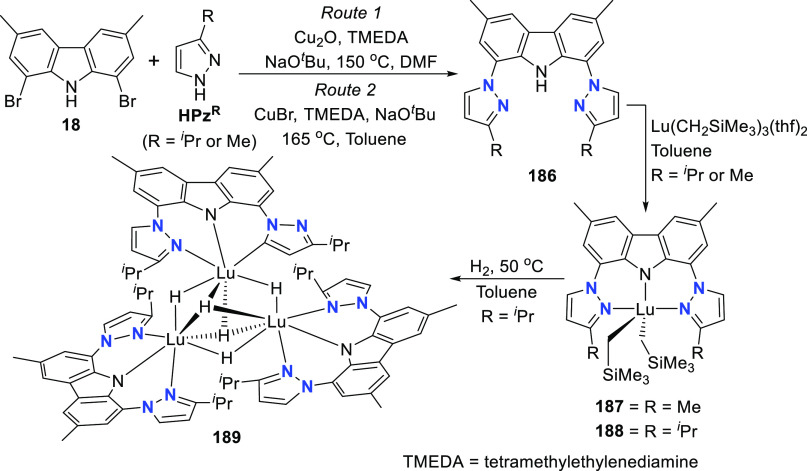
Lutetium Alkyl and Hydride Complexes Stabilized by a Bis(pyrazolyl)carbazolide

Substitution of the phosphinimine donor functionalities with pyrazole groups primed the carbazole-based NNN-pincer to stabilize lanthanide complexes against thermal degradation.^213,228^ As such, an Ullmann-type amination of dibromocarbazole **18** with the corresponding substituted pyrazole yielded ligand **186** featuring a methyl or isopropyl wingtip substituent further reacted with lutetium in an alkane elimination reaction leading to **187** and **188** (Scheme 29). It was reported that heating a solution of either **187** and **188** in C_6_D_6_ to 75 °C for 12 h did not lead to any complex decomposition. Complex **188** was further subjected to hydrogenolysis in an effort to prepare a lutetium hydride complex, which was successfully isolated and crystallized. The crystal structure evidenced the trimetallic complex **189** with five hydride ligands bridging the three metals. Of further note was the NNC-coordination mode of one of the NNN-pincer ligands to a Lu metal. Monoanionic carbazolide coordination was described for two of the Lu metals, with NNN-pincer coordination as expected. The third metal is coordinated by a dianionic carbazole ligand, a result of pyrazole C–H bond activation. It was theorized that intramolecular C–H bond activation occurred via metalation of the carbon of the pyrazolyl donor moiety with concomitant H_2_ elimination.^228^ This could further suggest hemilabile decoordination of the donor group with subsequent deprotonation of the pyrazolyl C–H by the hydride ligand. Alternatively, it was reasoned that metalation could have occurred prior to H_2_ formation by elimination of tetramethylsilane from **188**, a scenario less likely due to the complex’s high thermal stability. Hydrogenolysis of the sterically less encumbered complex **187** only resulted in decomposition, highlighting the requirement for steric shielding at the wingtip position. The presentation clearly demonstrates the donor influence on the complex, allowing for a more thermally robust complex that is not inert, therefore mediating the bond activation process.

### 3.2 Increased Metal Nucleophilicity Imparted by Electron-Donating Flanking Groups

**Figure 5:**
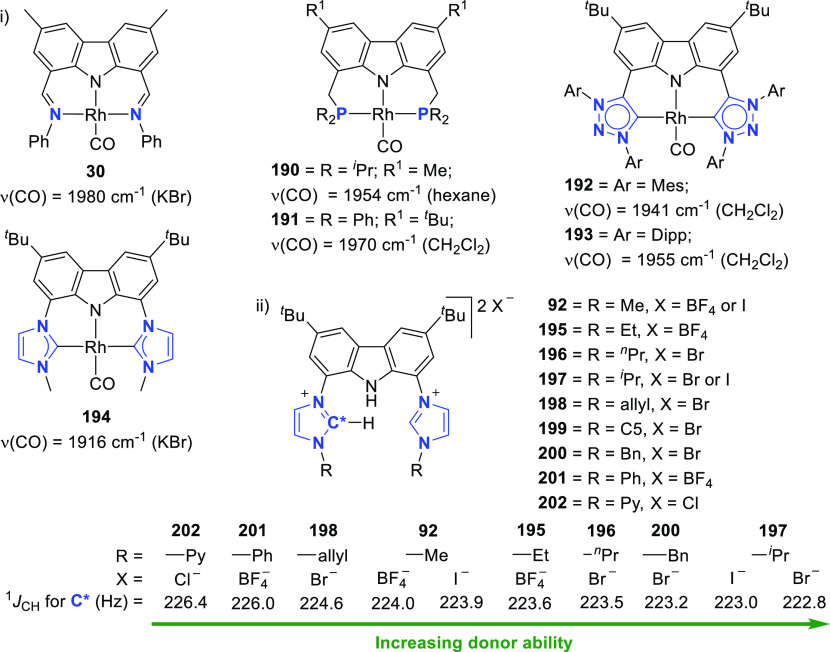
Increasing ligand donor strength though modification of the flanking donor groups.

**Scheme 30:**
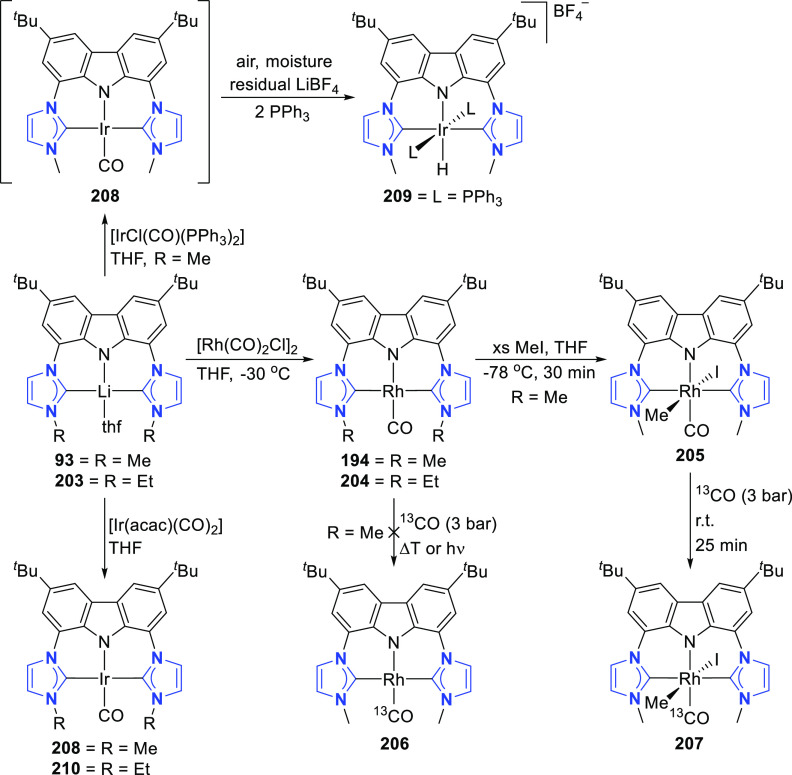
Synthesis and Reactivity of Rhodium and Iridium Complexes of BIMCA

One of the most obvious benefits of the **LNL**-carbazole scaffold is the ease with which the identity of the flanking donor groups **L** can be varied for direct modulation of the electronic consequence at the metal center. This is illustrated by the wide range of carbonyl stretching frequencies (1916–1980 cm^–1^) reported for square planar rhodium(I) carbonyl complexes **30**, **190**–**194** coordinated to the **LNL**-carbazolide (i, Figure 5) and follows the expected trend with ν_CO_ decreasing when **L** is varied from N(imine) donors (**30**, vide supra section 2.1, Scheme 4)^139^ to phosphines (**190**,^158^**191**,^154^*vide infra*, section 4.4, Scheme 63) to C(carbene) donors (**192**–**194**, *vide infra*, Schemes 30 and 48).^140,196,229^ On the one end of the spectrum (i, Figure 5), less basic donor groups can function as hemilabile ligands, rendering the ligand coordination noninnocent as described for the *s*- and *p*-block metal complexes of the bis(imine)carbazolide ligand (as coordinated in **30** in Figure 5) in section 3.3.1.^120,230^ On the other hand, the carbazole-N scaffold already provides for a strong donor platform (section 2), which can be maximized with the use of strongly basic donor groups such as NHCs, increasing the propensity of the electron rich metals to favor classic two electron processes, e.g., oxidative addition as described in sections 2.1 and below. For the rhodium carbonyl complexes reported with **L** = C(carbene), the BIMCA coordinated **194** exhibits the lowest CO wavenumber,^140^ demonstrating that it is the strongest donor ligand even compared to the triazolylidene analogues **192** and **193**.^196^ This result is surprising, as the mesoionic triazolylidenes are generally reported to be stronger donors compared to NHCs.^194,195^ One explanation for this contradiction could be the wingtip **R**-groups, as they also provide for a remote handle on the electronic properties at the metal center. This is evident comparing the difference in carbonyl ligand vibrations even within the same class of donors, i.e., **192** (1941 cm^–1^) and **193** (1955 cm^–1^) (Figure 5).^196^ Extrapolating to compare **192**/**193** with **194**, it means that the presence of four aryl groups instead of small aliphatic substituents on the flanking heterocycles can markedly alter the electronic consequence at Rh^I^.

The overall fine-tunability of the flanking donors by remote wingtip groups is illustrated clearly by inspection of the imidazolium C–H ^1^*J*_CH_ coupling constants of the library of available BIMCA ligand precursors (**92**, **195**–**202**, ii, Figure 5).^140,190^ The effects of the *N*-wingtip groups on the donor properties of the BIMCA ligand are discussed in more detail in section 4.3.3. A strong electron-withdrawing *N*-substituent increases the *p*-character of the N–C–H σ-bond in imidazolium, which in turn increases the *s*-character of the C–H bond and a larger C–H coupling constant. On the basis of the observed coupling constants, it was concluded that **197** with a bulky *N*-alkyl substituent (^*i*^Pr) is the strongest donor, while *N*-aryl-substituted **201** and *N*-heteroaryl-substituted **202** were identified as the weakest.

Kunz et al. accessed rhodium carbonyl complexes **194** and **204** of the BIMCA ligands (**92** and **195**) by transmetalation of *in situ* generated lithium complexes **93** and **203**, respectively, with [Rh(CO)_2_Cl]_2_ (Scheme 30).^140,231^ Analogous iridium complexes **208** and **210** were obtained by a similar method (Scheme 30).^232^ The most prominent structural feature in the crystal structures of the rhodium complex **194** and iridium complexes **208** and **210** is the strong distortion of the CO ligand out of the square planar geometry around the metal center.^140,232^ The possibility of increased lability of the ligand due to carbonyl distortion was ruled out in ^13^CO exchange experiments performed on rhodium complex **194** (Scheme 30). No exchange was observed despite overpressure of ^13^CO gas, irradiation, or prolonged heating, in accordance with the strong π-back-donation inferred from IR spectroscopy.^140^

The nucleophilicity of the Rh^I^ center in **194** as a result of the electron-donating nature of the BIMCA ligand was demonstrated by the formal oxidative addition of methyl iodide, whereby the observed immediate reaction led to the formation of **205** (Scheme 30).^140^ The X-ray crystal structure of octahedral **205** exhibits the iodine and methyl group at the axial positions *trans* to each other, typical of oxidative addition by the S_N_2 mechanism. The carbonyl group was shown to be more labile in **205** than in **194** based on ^13^CO exchange experiments, where carbonyl exchange was observed at room temperature within a short time (Scheme 30).

**Scheme 31:**
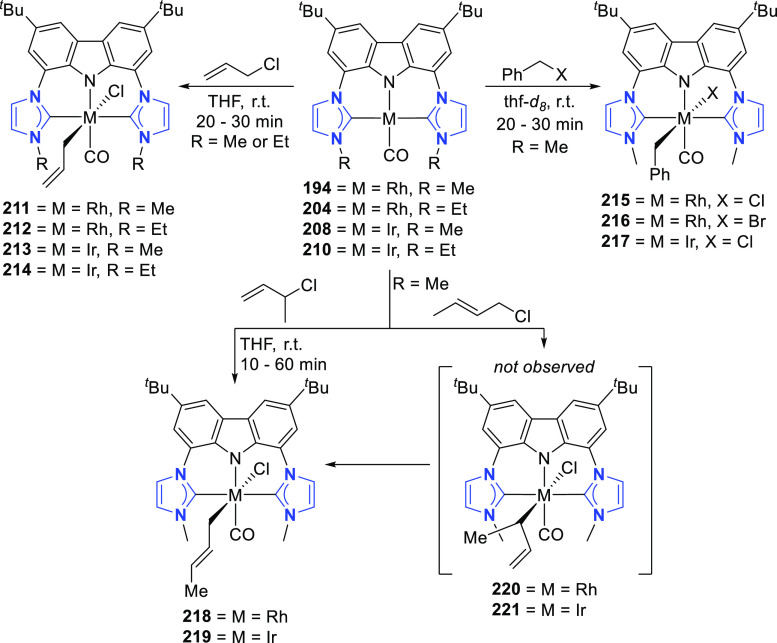
Reactivity of Group 9 Carbonyl Complexes of BIMCA with Allyl Halides

The rhodium and iridium complexes (**194**, **204**, **208**, and **210**) also showed reactivity toward allyl halides.^231,232^ The complexes reacted with allyl chloride and benzyl halides to the respective rhodium(III) and iridium(III) allyl complexes (**211** and **212**, M = Rh; **213** and **214**, M = Ir) and benzyl complexes (**215** and **216**, M = Rh; **217**, M = Ir) (Scheme 31). In the case of the rhodium complexes, it was reported that the reaction with benzyl chloride is much slower, attributed to the reaction with benzyl halides proceeding via an S_N_2 mechanism, while the reaction with allyl halides followed an S_N_2′ mechanism.^231^ Both the allyl and benzyl substituents in **211**–**214**, **216**, and **217**, respectively, are η^1^-coordinated to the metal.^231,232^ In addition, **194** and **208** were reacted with linear and branched methallyl chlorides (Scheme 31). Reaction with 3-chloro-1-butene yielded η^1^-allyl complexes **218** and **219** with an internal double bond (*E*/*Z* ratio = 5:1, reported for **218**) via an S_N_2′ reaction. The reaction with 1-chloro-2-butene also resulted in η^1^-allyl complexes **218** and **219**, instead of the expected **220** and **221** with a terminal double bond, the formation of which was not observed at all.

**Scheme 32:**
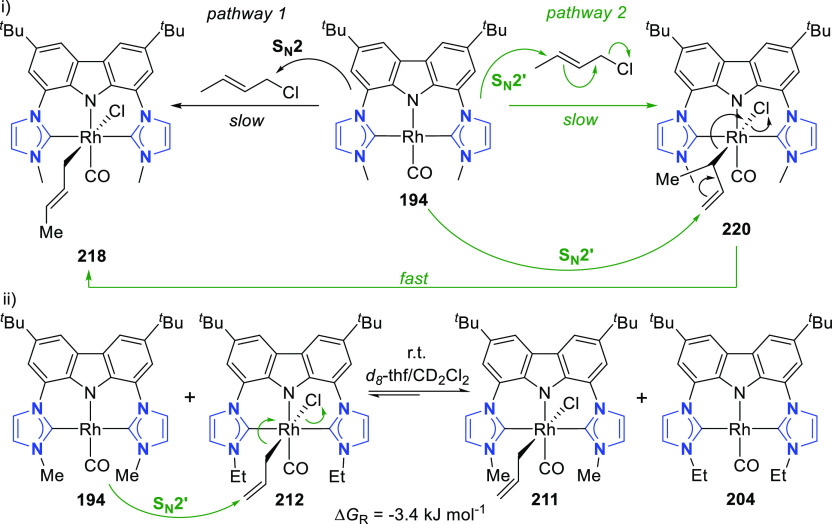
(i) Possible Pathways for the Formation of the Rhodium Complex **218** in the Reaction with 1-Chloro-2-butene and (ii) Crossover Experiment between **194** and **212**

In the case of rhodium complex **218**, one possibility considered was that the observed isomerization yielding the complex proceeds via a reactive η^3^-allyl intermediate as observed in palladium-catalyzed allylic alkylation.^233−236^ However, the possibility of this pathway was ruled out as unlikely, as the formation of the η^3^-isomer was not observed even under irradiation or at elevated temperatures.^231^ Two other pathways were found more likely, either by a direct S_N_2 mechanism or by a 2-fold S_N_2′ reaction via **220** (i, Scheme 32). The possibility of an S_N_2 mechanism was justified by the observation that the reaction proceeds more slowly with 1-chloro-2-butane than with 3-chloro-1-butene. However, as the reaction with 1-chloro-2-butene is faster than that with benzyl chloride, which typically proceeds by a S_N_2 mechanism and should be three times faster than the reaction with allyl chloride, the more likely pathway was considered to be a 2-fold S_N_2′ reaction. First an S_N_2′ reaction of **194** with 1-chloro-2-butene yields **220***in situ*, which then proceeds via an S_N_2′ mechanism with the highly nucleophilic Rh complex **194** to form the thermodynamically more favorable complex **218** with an internal double bond. A crossover experiment was performed in which **194** was reacted with **212** (ii, Scheme 32). Monitoring the experiment by ^1^H NMR spectroscopy indicated that two-thirds of the η^1^-allyl ligand was transferred from **212** to **194**, which resulted in the formation of **211** and **204**. After 5 h, the ratio of the components in the reaction mixture had not changed, indicating that **211** and **204** are thermodynamically more stable compared to **194** and **212**. This was also observed in the control experiment where **211** was reacted with **204**. DFT calculations supported the experimental observation of higher stability of **211** and **204**. On the basis of the above, it was concluded that in rhodium-catalyzed allylic alkylation the η^1^-allyl to η^1^-allyl isomerization can proceed without the formation of an η^3^-allyl intermediate.^231^ If the intermediate has a terminal allyl double bond, the reaction can proceed via the intermolecular route following the S_N_2′ metal transfer reaction.

**Scheme 33:**
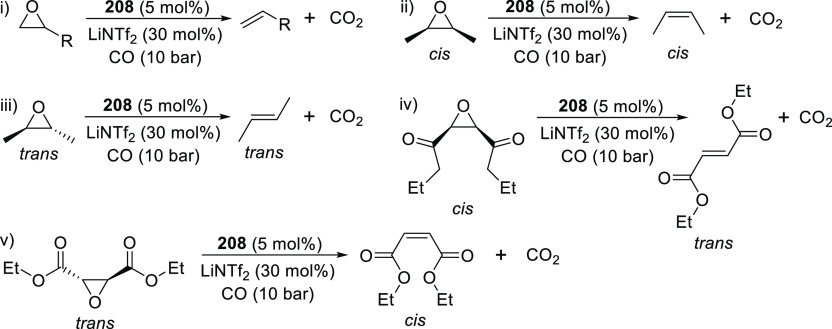
Catalytic Deoxygenation (in Solvent C_6_D_6_, 80 °C, 24 h) of (i) Terminal Epoxides, (ii) *cis*-2-Butene Oxide, (iii) *trans*-2-Butene Oxide, (iv) *cis*-Diethyl-2,3-epoxy Succinate, and (v) *trans*-Diethyl-2,3-epoxy Succinate with Carbon Monoxide

Expansion of the reactivity profile of the BIMCA complexes to catalytic epoxide deoxygenation was probed.^237^ Using carbon monoxide as a direct deoxygenation agent, **194** was found to catalyze the conversion of propylene oxide into propylene with Lewis acid LiNTf_2_ as the cocatalyst, although the complex proved to be unstable at the elevated temperature required for the reaction. However, the iridium(I) analogue **208** showed greater stability and considerably higher activity, which even exceeded the activity of commercially available rhodium, iridium, cobalt, and iron complexes under the same reaction conditions. **208** catalyzed the full conversion of propylene oxide to propylene under optimized reaction conditions (5 mol % catalyst loading along with 30 mol % of LiNTf_2_ cocatalyst under 10 bar of CO in benzene-*d*_6_ at 80 °C) (i, Scheme 33). The catalyzed deoxygenation of terminal alkyl epoxides (without significant isomerization to internal olefins) and aryl epoxides (especially electron-poor ones) proceeded efficiently, while side reactions increased with electron-rich aryl epoxides. Of particular note is the retention or inversion of configuration observed for 1,2-disubstituted epoxides upon deoxygenation. 1,2-Dialkyl epoxides retain the configuration as was exemplified by 2-butene oxide, the cis-isomer of which converts almost exclusively to *cis*-2-butene while *trans*-2-butene oxide is converted to *trans*-2-butene (ii and iii, Scheme 33). In contrast, inversion of configuration was observed with doubly ester-functionalized epoxides. This was illustrated by the deoxygenation of diethyl-2,3-epoxy succinate, the cis-isomer of which reacts to diethyl fumarate (*trans*) and the *trans*-isomer to diethyl maleate (*cis*) (iv and v, Scheme 33). The (stereo) selectivity of deoxygenation was explained by two different substrate-dependent epoxide activation mechanisms, either by oxidative addition with alkyl epoxides leading to retention of configuration or by S_N_2 mechanisms in the case of ethyl carboxylates, resulting in inversion of the configuration.

**Scheme 34:**
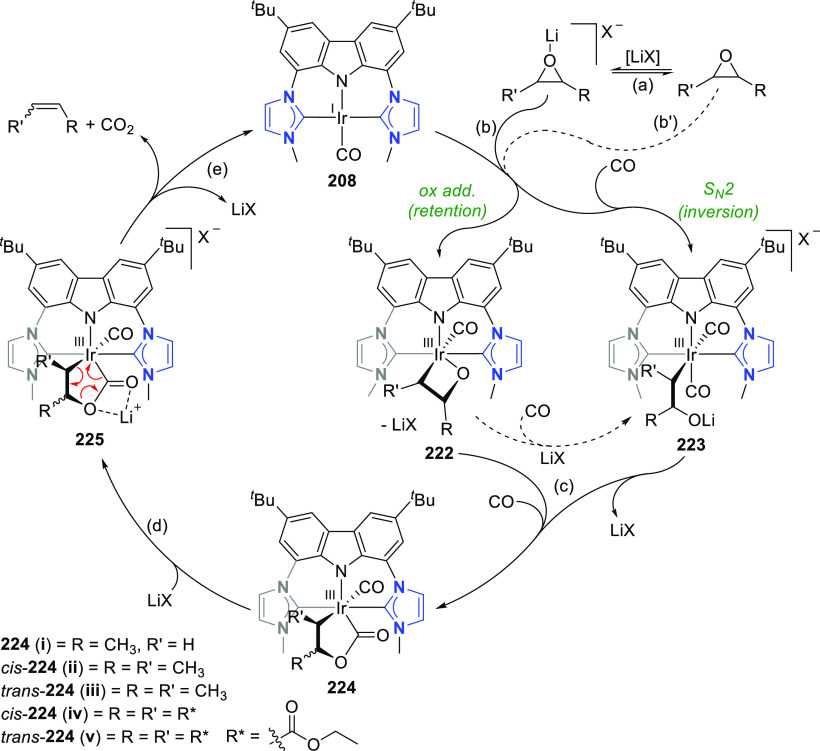
Proposed Mechanism for the Deoxygenation of Epoxides with CO Catalyzed by **208**

Further studies provided information on the mechanism of the catalytic reaction (Scheme 34).^237^ The 16-electron Ir^I^ complex **208** was identified as the catalytically active species, as it did not form an 18-electron CO-coordinated complex when exposed to 10 bar of CO. As the outcome of the catalytic reaction was found to be substrate-dependent, the intermediate steps of the catalytic cycle were therefore considered for both terminal alkyl epoxides and internal 1,2-di(alkyl or ester) substituted epoxides. In addition, the investigations were carried out both in the presence of a Lewis acid and without to clarify the role of the cocatalyst in the reaction. Activation of the epoxide requires the presence of a Lewis acid cocatalyst for coordination to epoxide oxygen.

In the case of the terminal epoxide, propylene oxide, it was observed that the activation also occurs without a Lewis acid, albeit slowly.^237^ In a reaction without Lewis acid, the formation and accumulation of intermediate **224** (**i**) (Scheme 34) with 2-irida-γ-lactone moiety was observed, the identity of which was verified by NMR and IR spectroscopy. When Lewis acid was present in the reaction, which was monitored by NMR spectroscopically, rapid formation of **224** (**i**) was observed. As the concentration of **224** decreases, product propene and active catalyst **208** is observed to form in the solution in addition to a new compound, identified as the Lewis acid adduct **225** (**i**) (Scheme 34). On the basis of these findings, it was concluded that the Lewis acid cocatalyst is not only necessary for preactivation of the epoxide but also mandatory for CO_2_ elimination (step e) (Scheme 34).

The retention of configuration observed for deoxygenation of 1,2-dialkyl substituted epoxides and the inversion observed for the ester-functionalized epoxides supported the conclusion that the activation of internal epoxides occurs by two different mechanisms (Scheme 34).^237^ With 1,2-dialkyl substituted epoxides, the opening of the epoxide ring (step b) most likely proceeds via oxidative addition under C–O bond cleavage to form the intermediate **222** where the configuration is preserved. An alternative S_N_2 mechanism would lead to inversion of configuration in the possible intermediate **223**, which is the more likely mechanism for the activation of ester-functionalized epoxides. In the case of 1,2-dialkyl substituted epoxides, the formation of intermediates **224** (**ii**) or **224** (**iii**) was not observed, indicating that the oxidative addition is the rate-determining step of the reaction. In a reaction involving ester-functionalized epoxide substrates without the presence of Lewis acid cocatalyst, the intermediates **224** (**iv**) (from *trans*-substrate) and **224** (**v**) (from *cis*-substrate) were detected by NMR spectroscopy. The single crystal X-ray structures of the intermediates **224** (**iv**) and **224** (**v**) confirmed the inversion of the configuration and the formation of the 2-irida-γ-lactone moiety. The mechanism (Scheme 34) thus derived for the **208** catalyzed deoxygenation of epoxides with CO, includes preactivation of the epoxide as a result of Lewis acid coordination (step a), followed by epoxide activation (step b) where the epoxide ring opens, either by oxidative addition (step b) allowing the retention of configuration or by the S_N_2 mechanism (step b′), leading to inversion of configuration. This is followed by CO-induced migration of the alkoxide from Ir to a CO ligand to form the intermediate **224** (step c) which forms a Lewis acid adduct **225** (step d), with subsequent decarboxylation to reconstitute the active catalyst **208** with product release (step e).

**Scheme 35:**
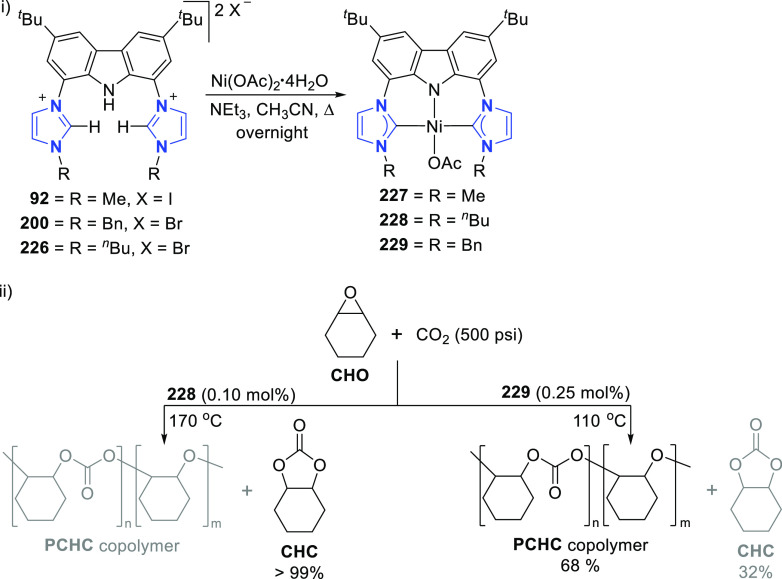
(i) Synthesis of Nickel(II) Complexes of Bis(imidazolylidene)carbazolide, and (ii) Coupling of Carbon Dioxide with Cyclohexane to Cyclohexane Carbonate Catalyzed by **228** and Poly(cyclohexane Carbonate) Catalyzed by **229**

Lee and co-workers employed modified BIMCA complexes of nickel(II) as switchable catalysts for cycloaddition or copolymerization of epoxides and carbon dioxide, demonstrating that not only the electron donating ability of the ligand but also the N-substituent wingtips in the donors adjacent to the NHC influence the catalytic performance (see section 4).^238^ The bis(imidazolium)carbazole precursors **92**, **226**, and **200** were reacted with excess triethylamine and Ni(OAc)_2_·4H_2_O (OAc = acetate) to yield the air- and moisture-stable nickel(II) acetate complexes **227**–**229** (i, Scheme 35),^238^ which are analogous to the nickel(II) complex **357** reported by Grotjahn (*vide infra*, section 4.3.1).^239^ The ^1^H NMR spectra of the complexes display resonances for the methyl protons of the acetate group at δ 1.68, 1.91, and 2.09 ppm for **229**, **228**, and **227**, respectively.^238^ The observed trend of increasingly downfield chemical shifts indicates the influence of the wingtip groups on the electronic environment.

All complexes **227**–**229** were active in the cycloaddition of cyclohexene oxide (CHO) and CO_2_ to produce cyclohexene carbonate (CHC) without the presence of a cocatalyst.^238^ However, **228** outperformed the other two in its catalytic activity, showing activity with a TOF of 41 h^–1^ and quantitative conversion of CHO (99%) with almost full *cis*-CHC selectivity (>99%) under optimized conditions of 0.1 mol % catalyst loading and CO_2_ pressure of 500 psi at 170 °C (ii, Scheme 35). The results showed that the coupling activity of the complexes decreases by the order of **228** > **227** ∼ **229**. This is presumably due to the electronic effect of the wingtip groups, whereby the more electron-donating ^*n*^Bu wingtip groups in **228** induce more nucleophilicity toward the acetate group for epoxide ring opening, which in turn leads to the observed higher catalytic activity of **228** in CHO/CO_2_ cycloaddition. However, further studies showed that as the catalyst loading is increased and the reaction temperature is decreased, complexes **229** and **227** catalyze the copolymerization of CHO and CO_2_ to poly(cyclohexene carbonate) (PCHC) instead of cycloaddition. A higher CHO conversion was achieved with **227**, while a better copolymer selectivity was associated with the presence of **229**. Under optimized conditions (0.25 mol % catalyst loading, CO_2_ pressure of 500 psi at 110 °C), **229** catalyzed the CHO/CO_2_ copolymerization into a narrowly dispersed poly(cyclohexene carbonate) with >99% carbonate-linkage content with moderate copolymerization selectivity (68% copolymer) (ii, Scheme 35).

**Scheme 36:**
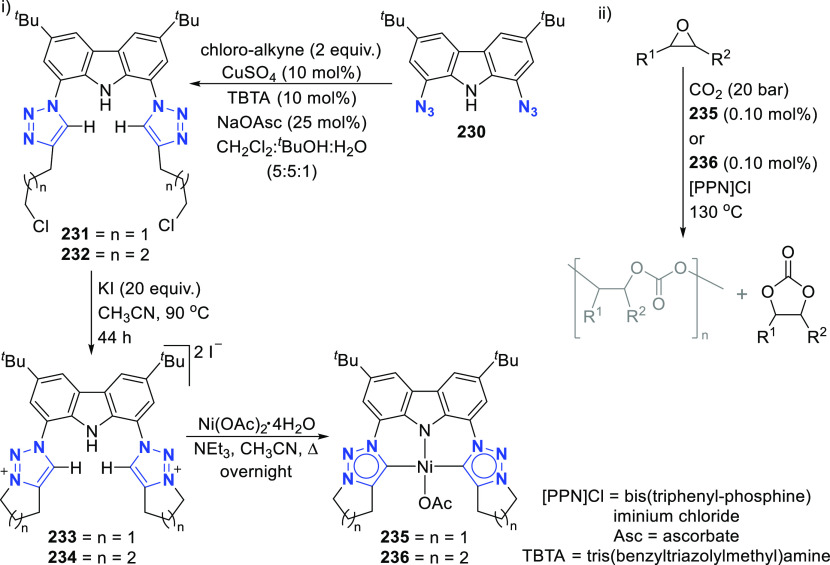
Synthesis of (i) Bis(triazolylidene)carbazolide Ligand Precursors and Their Nickel(II) Complexes and (ii) Cyclization of Epoxides and CO_2_ to Cyclic Carbonates Catalyzed by **235** and **236**

Hohloch et al. also reported the coupling of carbon dioxide and epoxides mediated by a bis(carbene)carbazolide nickel(II) complex, but their approach involved mesoionic carbenes, namely *N*-fused triazolylidenes, as carbazolide flanking groups.^240^ The change in the **L**-donor group from NHC to the stronger σ-donor MIC^194,195^ was anticipated to provide access to a more catalytically active complex. Ligand synthesis involved a copper-catalyzed alkyne azide cycloaddition (CuAAC) under standard conditions between 1,8-diazido-3,6-di-*tert*-butylcarbazole **230** and a chloro-alkyne, 5-chloro-1-pentyne, or 6-chloro-1-hexyne, yielding the corresponding chloroalkyl-triazoles **231**(240) and **232**,^241^ which were treated with an excess of potassium iodide to give the bis(*N*-fused triazolium)carbazole salts **233**(240) and **234**,^241^ respectively (i, Scheme 36). Reaction of the ligand salts **233** and **234** with Ni(OAc)_2_·4H_2_O in the presence of excess triethylamine afforded the respective nickel(II) acetate complexes **235** and **236** (i, Scheme 36).^240^ In **235** and **236**, the wingtip groups of the ligands differ only in one methylene group, but it was found to significantly affect the solubility of the complexes. Complex **236** was soluble in several coordinating, aromatic, and halogenated solvents, while **235** proved to be sparingly soluble in halogenated and coordinating solvents and insoluble in organic solvents.

The catalytic activity of **235** and **236** was tested in the copolymerization of carbon dioxide and epoxides under the same conditions as Lee had reported for the nickel(II) complexes **227**–**229**,^238^ except for a lower carbon dioxide pressure of 290 psi.^240^ However, **235** and **236** did not show significant activity in the coupling of carbon dioxide and cyclohexene oxide to polycarbonate. Instead, the replacement of NHC with MIC flanking donors led to an inversion of selectivity rather than the expected increase in catalytic potential, and the cyclization of carbon dioxides and epoxides to cyclic carbonates was observed instead of polycarbonate formation. The complexes **235** and **236** catalyzed the transformation of several epoxides into cyclic carbonates under pressurized carbon dioxide with a catalyst loading of 0.1 mol % at 130 °C (ii, Scheme 36). The general trend confirms the positive effect of the presence of cocatalyst bis(triphenylphosphine)iminium chloride ([PPN]Cl) on the activity and/or selectivity of the catalytic system. In addition, **236** showed higher catalytic activity than **235** which was concluded to be related to its better solubility.

### 3.3 Introducing a Noninnocent Handle at the EL-Group

#### 3.3.1 Ligand-Assisted Reactivity for *s*- and *p*-Block Metal Complexes

**Scheme 37:**
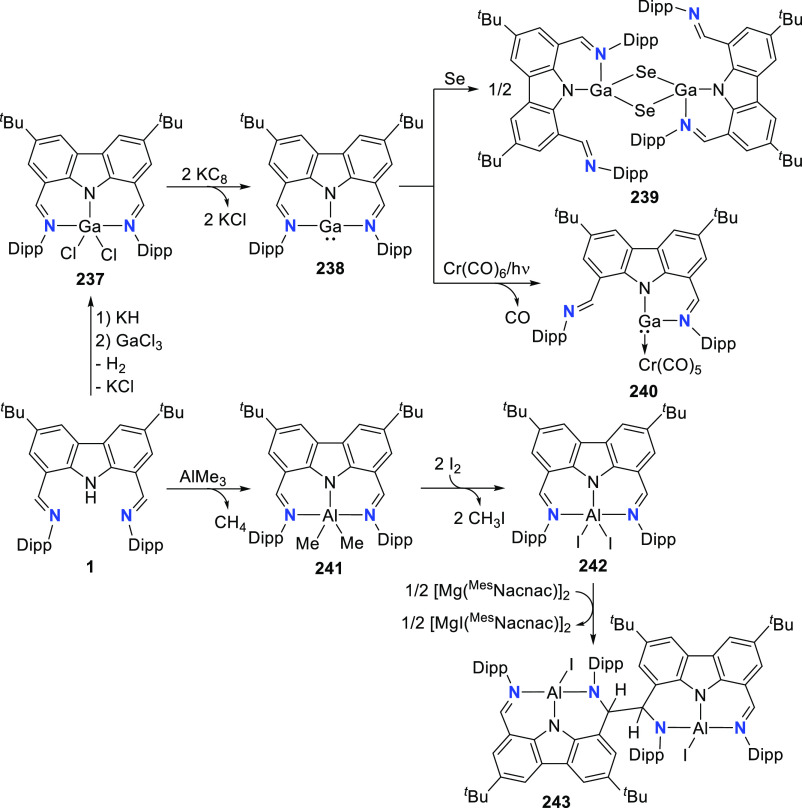
NNN-Carbazole Pincer with Imine Donor Groups Exhibiting Hemilabile Coordination

The stabilizing attributes imparted by the tridentate carbazole-based ligand were utilized toward the preparation of the first example of a pincer coordinated gallylene, with hemilabile coordination evidenced during subsequent reactivity studies on **238**.^230^ Ligand **1** was treated with KH, followed by GaCl_3_ in THF leading to the corresponding dichloride **237** (Scheme 37). Reacting **237** with two equivalents of KC_8_ yielded **238** which features a lone pair of electrons at the Ga metal, calculated to have a high s-orbital character. Reacting **238** with selenium powder led to dinuclear complex formation, with one of the imine donor moieties at each ligand decoordinating en route to **239**. No reaction occurred between **238** and Cr(CO)_6_. However, complete conversion of **238** to **240** was noted when subjecting a reaction mixture of **238** and Cr(CO)_6_ to light irradiation with a 250 W UV lamp. Again, hemilabile imine decoordination occurred toward **240**, with the lone pair of electrons on Ga coordinating the Cr(CO)_5_ metal. Ligand **1** was also coordinated to Al, leading to **241** that was further reacted with elemental iodine furnishing the aluminum diiodide **242** (Scheme 37). Reduction of **242** with [Mg(^Mes^Nacnac)]_2_ yielded the dinuclear Al^III^**243**, presumed to have formed through a one electron reduction of **242**, leading to an imino-based radical that undergoes dimerization and C–C bond formation. Crystal structure analysis confirmed the changes in bond lengths going from the imine in **242** to the amide of **243**. The reverse reaction of oxidation to yield the imine **242** was not reported and would be interesting to determine if such reactivity is feasible, which could potentially lead to a redox switchable catalyst with the redox reactivity facilitated across the imine donor wingtip.

**Scheme 38:**
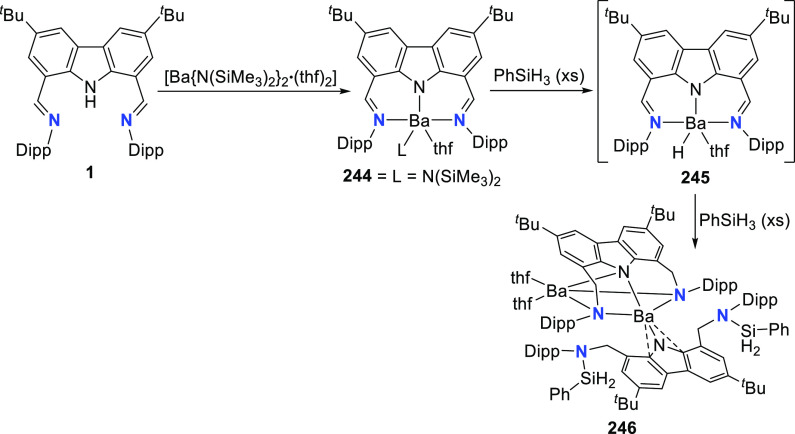
NNN-Carbazolide Barium Complexes with Imine Donor Groups

Imine reduction was also disclosed for the barium-coordinated bis(imine)carbazolide **244**.^120^ The reduction reactivity was accompanied by imine hydrosilylation during attempts toward a barium hydride complex. Accordingly, reacting **244** with PhSiH_3_ resulted in the dinuclear barium complex **246** (Scheme 38). The authors hypothesized the formation of dinuclear **246** through imine reduction by a putative barium hydride **245**, yielding the amide while imine hydrosilylation resulted in reductive decoordination of the second ligand’s imine moieties (Scheme 38). The order of reduction reactivity was not stated.

#### 3.3.2 Ligand-Assisted Reactivity for Early Transition Metal Complexes

**Scheme 39:**
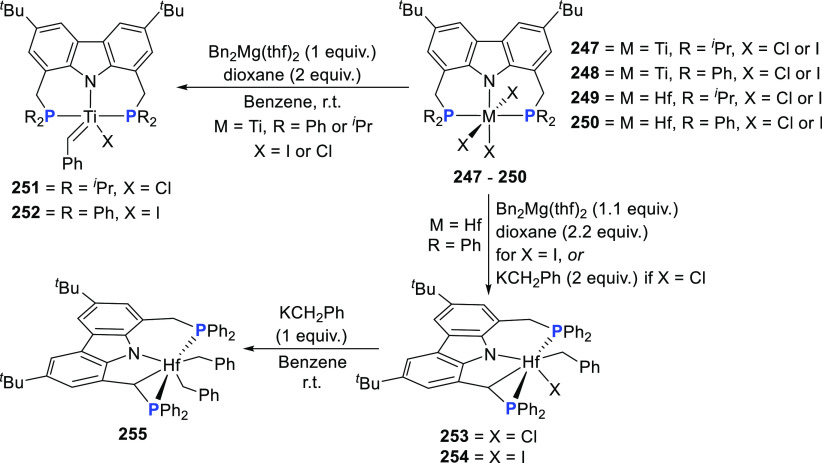
Synthesis and C–H Activation Reactivity of Cyclometalated PNP-Pincer Complexes of Group 4 Metals

**Scheme 40:**
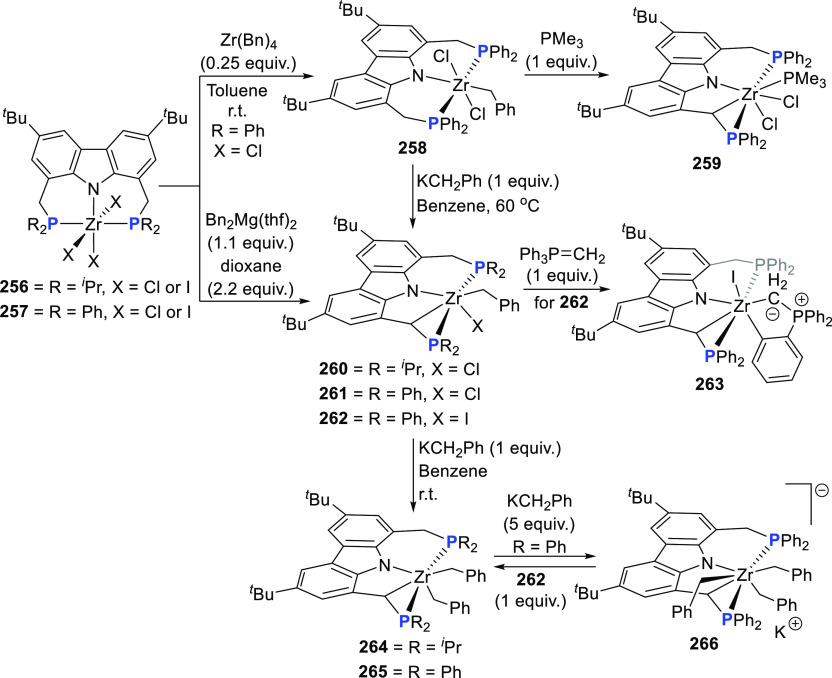
Reactivity of Cyclometalated Zr Complexes

The PNP-ligand precursor **36** (*vide supra*, Scheme 5) previously reported by Gade et al.^154^ could be modified by introducing isopropyl phosphine substituents as an alternative to the phenyl phosphorus substituents in the protioligand **312** (see Scheme 46).^173^ As for the aforementioned PNP-Ln catalysts **132**–**134** (Scheme 21), the combination of the hard amido and soft phosphorus donor functionalities served as rationale for the choice of ligands for the group 4 metals, in anticipated stabilization of both electron-rich and electron-poor metal atoms. The trihalegenido PNP-pincer complexes **247** and **248** (M = Ti^IV^), **249** and **250** (M = Hf^IV^), (Scheme 39) and **256** and **257** (M = Zr^IV^) (Scheme 40) were prepared following ligand precursor deprotonation and metalation with the appropriate tetrachloride metal precursor to yield the trichlorido complexes, whereafter ligand exchange with trimethylsilyl iodide resulted in the corresponding triiodido complexes. Titanium complexes **247** and **248** were found to be suitable precursors for the alkylidene complexes containing Ti=C bonds, **251** and **252**, respectively, obtained from the reaction with dibenzylmagnesium tetrahydrofuran with elimination of toluene. The analogous reaction with the hafnium complexes **249** and **250**, however, did not yield related alkylidene complexes. Instead, the cyclometalated monoalkyl complexes **253** and **254** were isolated (Scheme 39). Similarly, the zirconium trihalogenido complexes **256** and **257** (Scheme 40), yielded analogous complexes **260**–**262**, with the single crystal X-ray structure obtained for **262** exhibiting an η^3^-coordination mode of the benzyl ligand and confirming the tetradentate nature of the carbazolide ligand. This different reactivity of **249**, **250**, **256**, and **257** compared to the Ti complexes **247** and **248** could be ascribed to the longer metal-ligand bonds and the resulting differences in the orientation of the phosphino-methylene units within the coordination sphere. The presence of the α-CH_2_ units adjacent to the phosphine donors dominates the alkylation reactivity patterns of these group 4 metal complexes to kinetically favor cyclometalation over α-hydrogen abstraction.^173^ The intramolecular C–H activation of the phosphine methylene could not be controlled; however, it could be suppressed in a “comproportionation” reaction with tetrabenzyl zirconium(IV) to yield the thermally unstable **258** (Scheme 40). In this case, the crystal structure indicated η^2^-bonding of the benzyl, and although the P–Zr–P bond angle (157.59(4)°/154.66(4)°) of **258** deviates significantly from linearity as a structural consequence of the tilted arrangement adopted by the carbazole backbone, no C–H activation and cyclometalation were observed. **258** could be deprotonated by benzyl potassium at elevated temperatures to proceed to the cyclometalated **261** (Scheme 40). Alternatively, donor-induced hydrogen atom abstraction and cyclometalation were facilitated by the addition of 1 mol equiv of trimethyl phosphine to **258**. One of the ligand methylene groups was deprotonated to yield cyclometalated **259** with concurrent loss of toluene.

The remaining coordination site occupied by the halogenido ligands in **253**, **254**, and **260**–**262** was readily benzylated by treatment with benzyl potassium to afford the dibenzyl cyclometalated complexes **255** (M = Hf, Scheme 39) and **264** and **265** (M = Zr, Scheme 40), respectively.^173^ In this instance, one benzyl ligand was found to be coordinated in a η^3^-fashion, while the second displayed η^2^-coordination for the zirconium complexes **264** and **265** compared to the η^1^,η^3^-bonding exhibited by the dibenzyl hafnium complex **255** (Scheme 39). Further reaction with excess benzyl potassium results in the formation of the anionic tribenzyl zirconate **266**, which could be comproportioned with **262** to regenerate **265** (Scheme 40). An *ortho*-metalated methylene triphenylphosphorane complex **263** was produced by reaction of **262** with methylene triphenylphosphorane. The five-membered metallacycle containing the ylide carbon atom is slightly puckered, as shown in the crystal structure of this complex, and confirms sp^3^ hybridization at the ylide carbon.

**Scheme 41:**
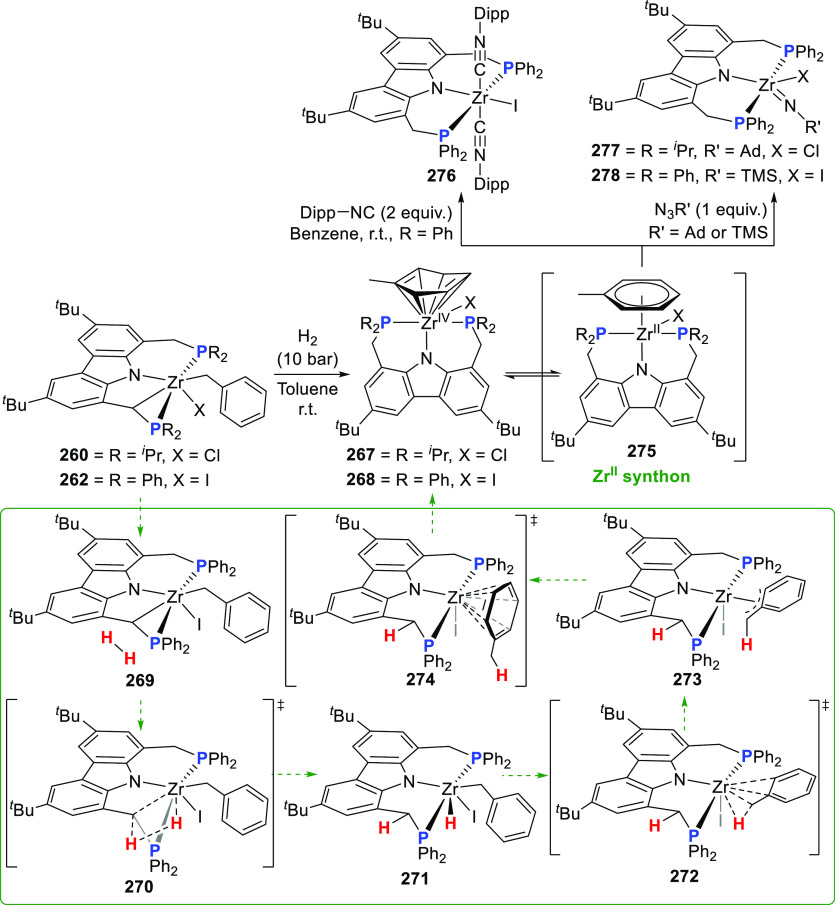
Mechanism of Hydrogenolytic Formation of η^6^-Arene Zr PNP-Pincer Complexes and Their Reactivity As Zirconium(II) Synthons

The suitability of cyclometalated zirconium complexes **260** and **262** as candidates to serve as zirconium(II) synthons was considered due to the presence of a PNP-pincer ligand that not only stabilizes a variety of coordination geometries but also potentially has redox states lower than + IV.^242^ This is essential for the development of stoichiometric and catalytic group 4 metal chemistry that does not merely involve redox-neutral transformations.^243−245^ Toward this effort, **260** and **262** were treated with molecular hydrogen, leading to the hydrogenolysis of the cyclometalated Zr–C bond to yield zirconium η^6^-arene complexes **267** and **268** (Scheme 41).^242^ Formally, the puckered arene is formulated as an arene-1,4-diido species, rendering these compounds as Zr^IV^ species. The folding of the arene ligand was attributed to the metal-to-ligand δ back-bonding of the zirconium *d*_*xy*_-orbital into the LUMO of the arene, confirmed by visualization of the HOMO of **267** with DFT calculations. For a zirconium(II) metal, the complex HOMO would have been a metal-localized nonbonding orbital. However, of the two resonance forms displayed for (**267** and **268**)/**275** in Scheme 41, the zircona-norbornadiene formulation (**267** and **268**) appears to adequately represent the ground state metal valency (+IV) and metal-ligand bonding interaction. However, it also means that the displacement of the neutral arene by other ligating moieties should provide access to Zr^II^ complexes, validating **275** as Zr^II^ synthon stabilized by the PNP-pincer.

The hydrogenolysis mechanism of **260**/**262** was postulated to proceed through a labile hydrido zirconium intermediate too short-lived to be detected directly, with consequent reductive elimination leading to the η^6^-arene complexes **267**/**268**.^242^ Deuteration studies confirmed an intramolecular reaction sequence after the initial hydrogenolysis of the Zr–C bond in the cyclometalated pincer ligand, but the individual reaction steps of the initial hydrogenolysis remained ambiguous. Either hydrogenolysis at the benzyl-C–Zr gives rise to a η^6^-toluene hydrido intermediate in which the PNP ligand remains cyclometalated, followed by reductive elimination, or hydrogenolysis by σ-bond metathesis occurs first at the cyclometalated ligand to yield a hydrido-benzyl intermediate. DFT modeling of the two alternative reaction pathways for **268** formation was performed, and the most plausible reaction pathway is presented in Scheme 41 (bottom). Approach of a dihydrogen molecule to yield intermediate **269** is followed by intramolecular heterolytic splitting of dihydrogen to protonate the methyne group of the ligand in **270** to yield the zirconium benzyl hydrido intermediate **271**, significantly more stable than the alternative η^1^-toluene hydrido intermediate computed. Hereafter, the terminal hydride in **271** attacks the methylene group of the benzyl ligand via **272** and **273** to form the zirconium(II) η^6^-toluene transition state **274** through C–H coupling with subsequent rearrangement to the ground state structure of **268**.

Vindication of the supposed role of **268**/**275** as the Zr^II^ synthon was obtained by the reaction with 2,6-diisopropylphenyl isocyanide (Scheme 41).^242^ Clean conversion to the mononuclear **276** was observed, while the computed HOMO was interpreted as consisting primarily of a Zr-centered *d*-orbital lone pair engaged in back-bonding interactions into the C≡N π* bonds to validate its description as a zirconium(II) species. The further demonstration of **267**/**268** as zirconium(II) precursors **275** was performed by reaction of **267** with 1-azidoadamantane, and **268** with trimethylsilylazide, to yield the imido complexes **277** and **278**, respectively, with the release of toluene and evolution of nitrogen (Scheme 41). The azides act as two-electron oxidants where the “masked” Zr^II^ reagents **275** are oxidized to the corresponding Zr^IV^ species **277** and **278** during the Zr=N bond formation.

**Scheme 42:**
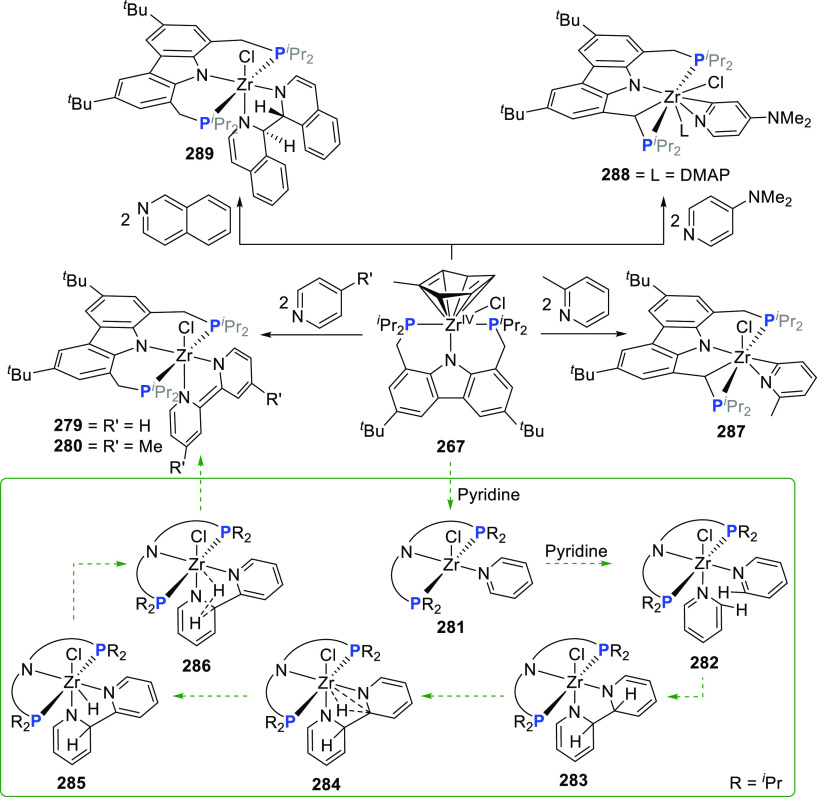
Dehydrogenative Coupling of Pyridines Mediated by PNP-Zr^II^ Synthon

Addition of pyridine to **267** (Scheme 42) also displaces the toluene ligand, resulting in complex **279** with a coordinated 2,2′-bipyridine (bpy) ligand,^242^ through a combination of C–H activation and C–C coupling and molecular hydrogen release, as a unique reactivity reported for group 4 metals.^246−248^ The structural data obtained from the X-ray structure of **279** indicated distinctly different C_py_–C_py_ and C–N bond lengths, corroborated by spectroscopic studies to identify the bonding of the bipyridine as a dianionic, diamido-type ligand (bpy^2–^), resulting from the reductive coupling of pyridine facilitated by a transient Zr^II^ species. The scope of this transformation and the cooperative reactivity of the PNP ligand in the mechanism of the transformation were investigated by including substituted pyridines as substrates.^249^ For pyridines methylated in the *meta*-position, unselective conversion to a variety of unidentifiable products was observed, while 4-picoline substrate yielded exclusively the analogous **280** complex with coordinated bpy^2–^ and concurrent loss of hydrogen (Scheme 42). Conversely, when the *ortho*-methylated pyridine was employed as a substrate, resultant complex **287** displayed a cyclometalated pincer backbone from one of the methylene bridges neighboring a phosphine donor (Scheme 42). Extending the range of 4-substituted pyridine substrates led to the emergence of a general reactivity trend: pyridine substrates with reduced electron density in the aromatic ring did not undergo reductive coupling, while the reaction with an electron-rich pyridine yielded the coupled heterocycle product along with significant amounts of side products. Only substrates with similar electronic properties to the unsubstituted pyridine, e.g., alkyl and sp^2^ substituted pyridines, gave the desired bipyridyl ligand. If, however, a substituent with a further increase in electron donating character was employed, such as *N*,*N*-dimethylamine in DMAP, selective conversion into the cyclometalated pyridyl complex **288** was observed (Scheme 42). As for **287**, *ortho* C–H activation of the pyridine leads to the formation of an η^2^-pyridyl, although an additional substrate (DMAP) molecule is coordinated to the metal in **288**. A chemically noninnocent role of the ligand backbone was deemed to be a possible pathway toward the reductive coupling of the pyridines, by way of an initial C–H activation step forming an (η^2^-pyridyl) zirconium(IV) hydride species. Repeating the reaction with pyridine-*d*_5_ did not lead to the expected incorporation of deuterium into the ligand backbone, whereby a cooperative cyclometalation step involving the PNP ligand backbone could be excluded from this reaction sequence.^249^ Thus, isolated complexes **287** and **288** are the result of a “dead-end” competitive reaction pathway rather than a reaction step en route to bpy^2–^ formation.

When isoquinoline was reacted with **267**, a C–C coupling reaction was indeed observed to occur, but without the loss of the two hydrogen atoms at the bridge carbons of product **289** (Scheme 42), with the solid state structures displaying an *anti*-disposition of the H atoms.^249^ To confirm that C–H activation of the pyridine via a Zr^IV^ and η^2^-pyridyl intermediate was not the most likely reaction pathway, DFT calculations for both the aforementioned route, as well as a route incorporating C–C bond formation as the first step, were carried out. The elevated activation barrier for the former route focused the study on an initial C–C coupling step of the two pyridines with either *syn*- or *anti*-orientation of the hydrogen atoms, relative to the formed bipyridine plane in the intermediate. The results indicated a *syn* C–C coupling sequence, as illustrated in Scheme 42 (bottom). The almost thermoneutral substitution of toluene by pyridine and concomitant formation of the low-valent Zr^II^ species **281** is followed by coordination of the second pyridine to form **282**. C–C bond formation leading to the Zr^IV^ species **283** is exergonic via **282** with singlet spin state. Subsequent isomerization to **284**, to position the C–H bond close to the zirconium, is endergonic but leads to facile C–H bond cleavage to form **285**. The second C–H bond cleavage is achieved through **286** in a strongly exergonic σ-bond metathesis to yield product **279**. The competitive dead-end pathway yielding **288** and **289**, however, were computed to show lower energy transition states (Scheme 42), compared to the analogous transition state for the unsubstituted pyridine substrate (excluded also by the deuteration experiments), demonstrating a clear preference for the pathway leading to the cyclometalated η^2^-pyridyl complexes. This result is in agreement with the experimental observations, where further reaction with another equivalent of substituted pyridine is precluded by the subsequent high lying transition states.

#### 3.3.3 Ligand Cooperativity for Late Transition Metal Complexes

**Scheme 43:**
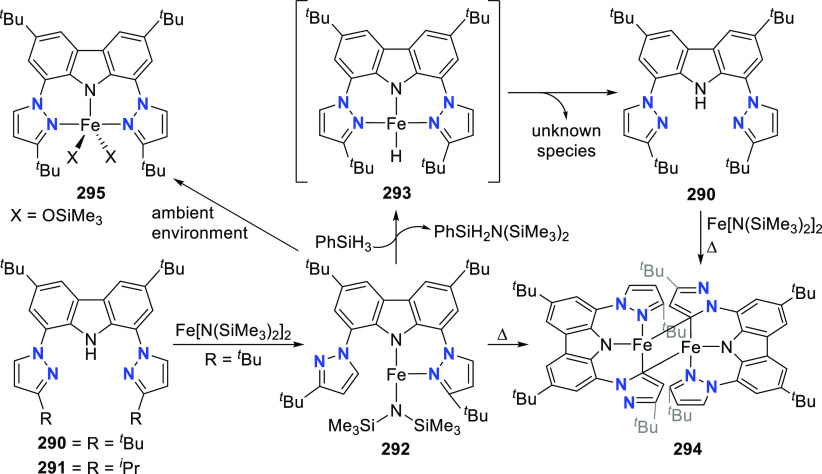
Synthesis and Reactivity of Hemilabile Bis(pyrazole)carbazolide Iron Complexes

The group of Lee focused their efforts on the bis(pyrazole)carbazole class of ligands,^250^ noting the advantage of incorporating the pyrazole donor group as a hemilabile site modulating complex reactivity.^251−253^ Accordingly, the synthesis of a carbazole coordinated iron bis(trimethylsilyl)amide complex was reported.^250^ The authors introduced steric bulk at the pincer’s donor wingtip sites **R** (Figure 1), which increased steric strain between the ligand and the bis(trimethylsilyl)amide coligand. The result is an air- and moisture-sensitive bidentate complex with an uncoordinated pyrazole donor group (**292**, Scheme 43), which leads to increased complex reactivity as was reported for the catalytic hydrosilylation reaction. In fact, 1 mol % of **292** catalyzed the hydrosilylation of various aryl and/or alkyl carbonyls with phenylsilane at ambient temperature in less than 60 min, with excellent conversion and TOFs reported. Attempted isolation of the reactive catalytic intermediate, postulated to be an iron hydride, proved nontrivial. The authors turned to calculations which validated the possible formation of three- and four-coordinate iron hydrido intermediates, with the four-coordinate iron hydride **293** lower in energy compared to the three-coordinate intermediate. Thus, “rollover” of the pyrazole donor moiety with subsequent coordination forming the pinced complex results in increased stability of the complex intermediate. Presumably then, its subsequent decoordination in the next catalytic cycle again increases the reactivity of the complex toward hydrosilylation.

In addition to the isolation of **292**, a trigonal bipyramidal complex **295** with tridentate carbazolide coordination was also reported, obtained due to exposure of **292** to an ambient environment (Scheme 43).^250^ The two OSiMe_3_ groups originate from the hydrolysis of N(SiMe_3_)_2_ and do not crowd the coordination environment around the metal, allowing for tridentate coordination. Heating a solution of **292** yielded the dinuclear complex **294** with dianionic tridentate coordination, in addition to bridging interactions between the ligand and the second metal. The dinuclear complex could also be prepared by heating a solution of ligand **290** and Fe[N(SiMe_3_)_2_]_2_.

**Scheme 44:**
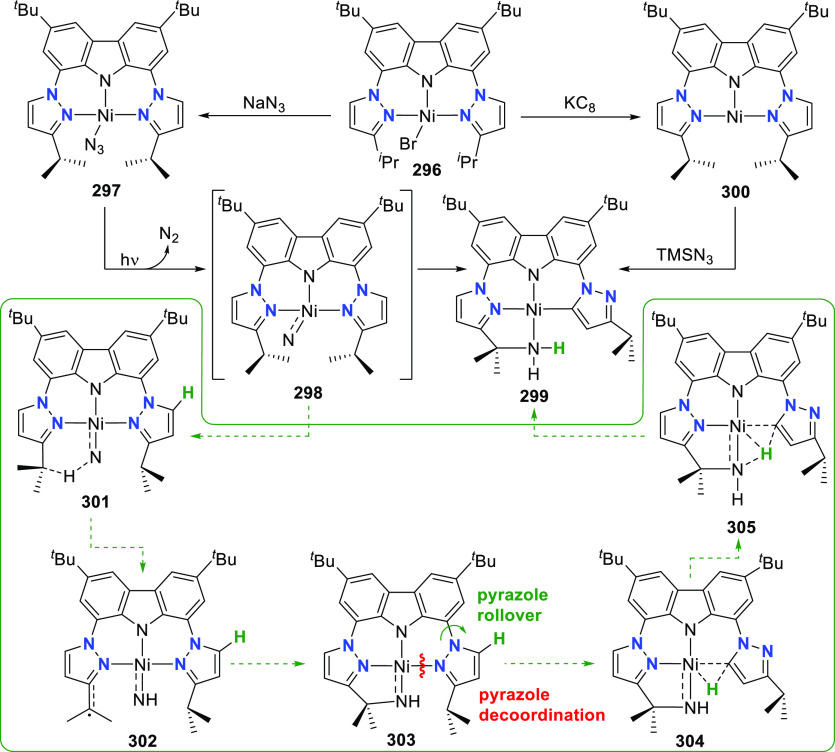
Double Intramolecular C–H Activation Leading to **299** via a Nickel-Nitridyl Intermediate

The NNN-carbazolide coordinated nickel bromide **296** could be prepared through the deprotonation of ligand **291** followed by *in situ* metalation with NiBr_2_. **296** was treated with NaN_3_, resulting in isolation of the targeted nickel azido complex **297** (Scheme 44).^254^ Irradiation of **297** yielded an orange-colored product in high yields. Evidence for unique reactivity was initially noted during an NMR spectroscopic investigation, which indicated a *C*_1_ molecular symmetry for the product, suggesting modification of the carbazole supporting ligand. This was confirmed with a crystal structure of **299**, which revealed an unprecedented double C–H activation. The first ligand-assisted reaction engages the C–H of the isopropyl wingtip moiety and the formed nickel nitridyl intermediate **298**, leading to isopropyl C–N bond formation. The second C–H activation step involves hemilabile pyrazole decoordination and rollover of the donor moiety at **303** leading to **304** (Scheme 44). Calculations suggest an agostic interaction to proceed after pyrazole rollover, followed by a [2σ + 2π] transition state with ensuing formation of the nickel amine complex **299**. A thermal route toward **299** was also probed, which was proposed to proceed via a Ni^III^ imido intermediate. Reduction of **296** with 1.5 equiv of KC_8_ leads to formation of the paramagnetic T-shape Ni^I^**300** with an *S* = 1/2 ground state. Both color change and gas evolution were noted when reacting **300** with TMSN_3_ and isolating **299** as the major product of the reaction (Scheme 44). Additional computational investigations supported the replacement of nickel for iron or cobalt, calculated to follow a similar reactivity profile as determined for the nickel, albeit with different energy profiles.^255^ Calculations further suggested that multiple photoexcitation events are required to drive the reaction forward, from photolysis of the azide to the rollover C–H activation step, especially in the case of Fe and Co.

**Scheme 45:**
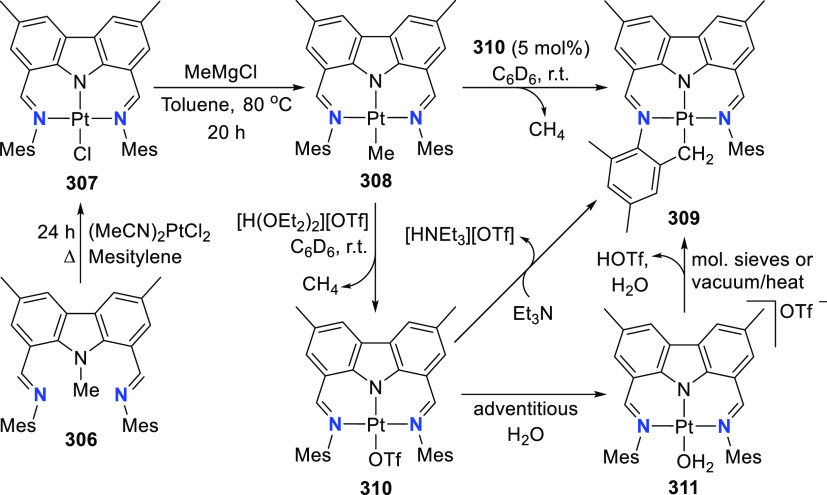
Wingtip C–H Activation Leading to Cyclometalative Reactivity

Changing the donor wingtips from the bulky Dipp groups to Mes substituents introduced another noninnocent handle on the bis(imino)carbazole NNN-pincer ligand.^256^ The handle was leveraged during the preparation of the cyclometalated platinum complex **309**, isolated after reacting **308** with 5 mol % **310** or with triethylamine which induced mesityl wingtip cyclometalation (Scheme 45). The cyclometalated **309** could also be obtained by subjecting **311** to vacuum conditions at elevated temperature or placing it over molecular sieves. Complex **311** was in turn isolated by reacting **310** with water (Scheme 45). The reactivity of the bis(imino)carbazolide showcases a hemilabile as well as redox noninnocent combinatorial effect, in addition to exhibiting cyclometalative reactivity through ligand donor wingtip group fine-tuning.

## 4 Wingtip Effects

### 4.1 Wingtip Sterics Controlling Reactivity at the Metal

**Scheme 46:**
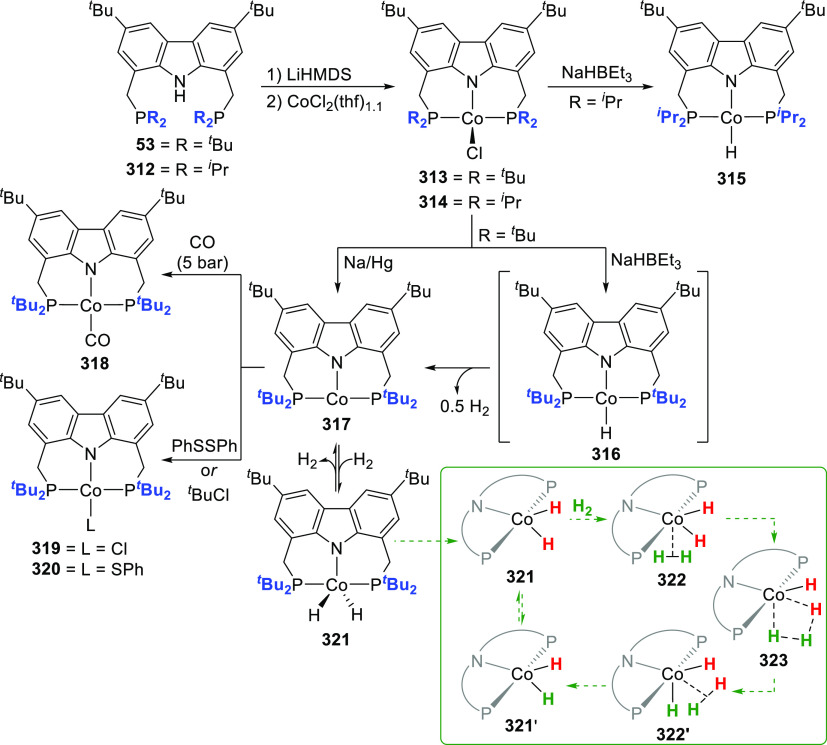
Synthesis and Reactivity of High-Spin Cobalt(II) Chlorido, Low-Spin Cobalt(II) Hydrido and High-Spin Cobalt(I) Complexes Supported by PNP-Ligands

The significant effect of the wingtip steric demand was demonstrated by the minor modifications of the PNP-carbazolide pincer wingtip groups.^257^ The achiral PNP-protioligands **312**(173) and **53**,^162^ containing isopropyl or *tert*-butyl substituents, respectively, on the donor phosphorus atoms were deprotonated and reacted with [CoCl_2_(thf)_1.1_] to yield the *d*^7^-high spin cobalt(II) chloride complexes **314** and **313** (Scheme 46).^257^ Both cobalt(II) complexes display distorted tetrahedral coordination, but with a notable disparity between the N(carbazolide)–Co–Cl bond angles: in the case of **313**, a decrease in the bond angle from 122.6° for **314** to 117.1° for **313** is observed due to the increased steric demand of the ^*t*^Bu groups compared to the ^*i*^Pr groups, resulting in the chlorido ligand seemingly “pushed” toward the carbazolide-nitrogen. This steric effect results in different reactivity of the cobalt(II) chloride complexes toward treatment with the hydride transfer reagent NaHBEt_3_. In the case of **314**, facile chloride substitution to yield the low-spin *d*^7^ cobalt(II)-hydride complex **315** takes place (Scheme 46). Few examples of isolated pincer-supported Co^II^–H complexes are known; the rarity is ascribed to the propensity of Co^II^ hydride complexes to undergo one-electron reductions.^258−260^ On the other hand, reaction of **313** with NaHBEt_3_ leads to the reduction of the cobalt(II) to yield a coordinatively unsaturated high-spin Co^I^ complex **317** with T-shape geometry conferred by the tridentate PNP pincer ligand (Scheme 46),^257^ only the second example of such a three-coordinate cobalt(I) complex.^261^ Presumably, reduction proceeds via the cobalt(II)-hydrido intermediate **316**, whereafter reductive elimination of molecular hydrogen results in the formation of **317**. Alternatively, direct reduction of **313** with reducing agent Na/Hg also yields **317** (Scheme 46).^257^

The reactivity of high-spin Co^I^ complex **317** was investigated by reaction with carbon monoxide, hydrogen, as well as *tert*-butyl chloride and phenyl disulfide (Scheme 46).^257^ Treatment of **317** with CO yielded the diamagnetic low spin *d*^8^ complex **318**, while exposure of **317** to a hydrogen atmosphere (10 bar) leads to the formation of the diamagnetic cobalt(III) dihydrido complex **321**. Under the given reaction conditions, an equilibrium between **317** and **321** was observed. Consequently, isolation of **321** was not possible, and deuteration studies were conducted with deuterium hydride gas to confirm dihydride formation and not dihydrogen coordination. Isotope scrambling was observed to occur, by way of bond scission processes of the dihydrogen isotopomers after an oxidative addition step with rapid H/D exchange. DFT calculations were employed to differentiate between the two possible reaction sequences for H/D exchange. Participation of the carbazole-amide moiety in hydrogen activation leading to a protonated amide was excluded by the highly endergonic H_2_ extrusion reaction step. Instead, a direct exchange pathway was found likely by proceeding through intermediate **322** after additional molecular H_2_ coordination (Scheme 46). H atom scrambling (**323**) and consequent σ-bond metathesis between coordinated dihydrogen and one hydride ligand leads to formation of **322′** with a dihydrogen coordinated trans to the amide donor. From **322′**, the reaction sequence proceeds with the extrusion of the coordinated H_2_ to form the “mixed” dihydride **321′**. Besides the two-electron process of oxidative addition leading to **321**, one-electron reductions were confirmed using the substrates favoring radical-type transformations, both reaction with ^*t*^BuCl and phenyl disulfide resulted in one-electron oxidations to yield **319** and **320**, respectively (Scheme 46).

**Scheme 47:**
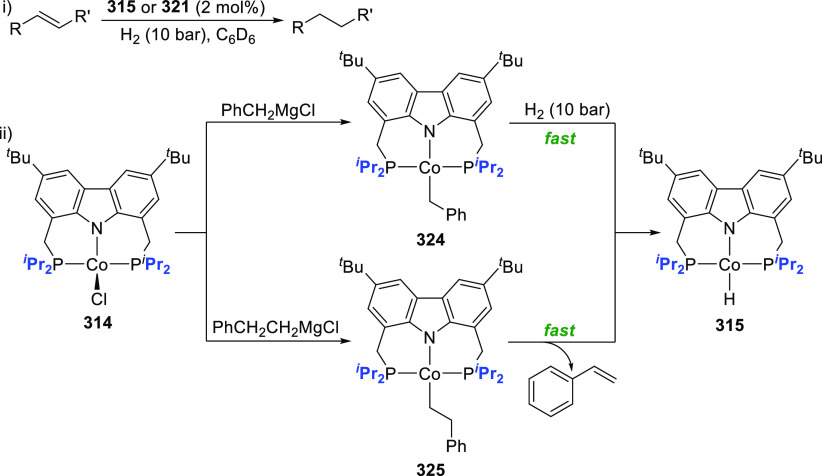
(i) Catalytic Alkene Hydrogenation and (ii) Stoichiometric Transformations to Model the Elementary Reaction Steps of the Alkene Hydrogenation Mechanism

The potential activity of Co^III^ dihydride **321** and the Co^II^ hydride **315** in the catalytic hydrogenation of alkenes was screened with substrate norbornene under dihydrogen pressure (10 bar) at room temperature.^257^**315** mediated the transformation readily at a catalyst loading of 2 mol % in under 5 min, but a slow transformation to norbornane was only observed for **321** at 60 °C (i, Scheme 47). Further investigation into the activity of **315** by substrate variation proved that although aryl-substituted terminal alkenes were readily hydrogenated, disubstituted alkenes were only converted over longer time periods or at elevated temperatures. This result points to a slow insertion step of the alkene into the stable Co–H bond of **315**. This mechanistic assumption was probed with stoichiometric transformations (ii, Scheme 47). **314** was treated with a solution of phenethylmagnesium chloride, directly forming **315** to verify a fast equilibrium between insertion of the alkene and predominant β-H elimination of the styrene to reform **315**. In a second elementary reaction, the σ-bond metathesis of H_2_ with the Co–C bond was demonstrated by the reaction of the benzyl complex **324** with H_2_ at elevated pressure. Rapid cleavage of the Co–C bond yields **315**, to confirm that slow insertion of the alkene is the limiting step. The insertion of the disubstituted alkenes is therefore less likely to proceed as the steric demand of the alkenes increases.

**Scheme 48:**
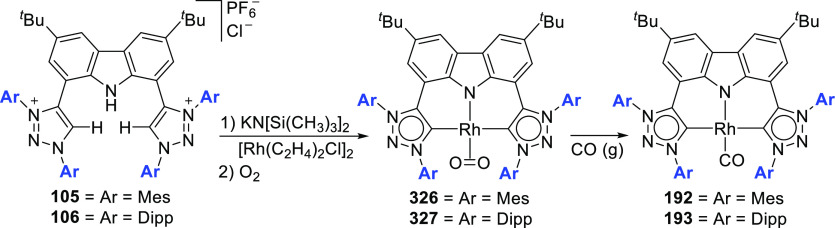
Synthesis of Rhodium(I) Complexes of Bis(triazolylidene)carbazolide

The wingtip steric effects were also pronounced with rhodium complexes of the bis(triazolylidene)carbazolide ligand employed in catalytic dimerization and hydrothiolation of alkynes.^196^ The rhodium complexes **326** and **327** were obtained by treatment of the ligand salts **105** and **106**, respectively, with excess KHMDS in the presence of metal precursor [Rh(C_2_H_4_)_2_Cl]_2_ followed by exposure to oxygen (Scheme 48). The crystal structure of **327** further confirmed the molecular structure of the complex, showing a square planar geometry around the rhodium(I) center with molecular oxygen coordinated in a side-on fashion. Treatment of **326** and **327** with carbon monoxide resulted in the formation of the respective carbonyl complexes **192** and **193**, useful as probes for the electron-donating ability of the CNC-ligands, as discussed in section 3.2.

**Scheme 49:**
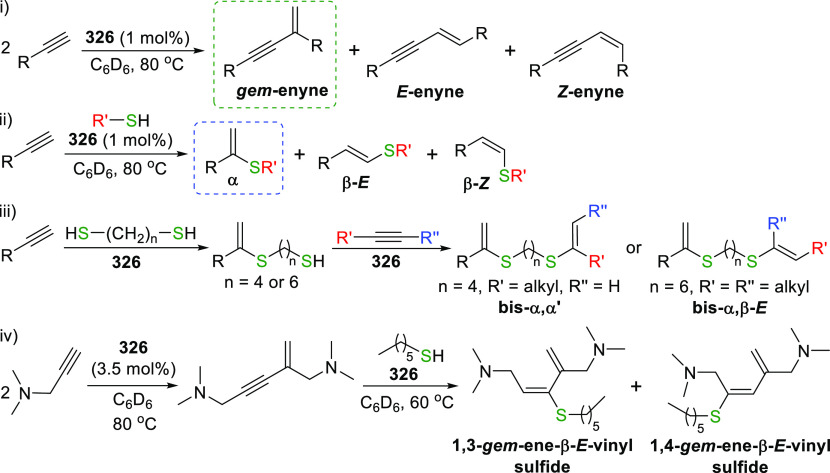
(i) Homodimerization of Terminal Alkynes, (ii) Terminal Alkyne Hydrothiolation, (iii) Bis-Hydrothiolation in a Sequential One-Pot Reaction of Dithiol with Various Alkynes, and (iv) Sequential Alkyne Dimerization and Hydrothiolation

The catalytic activity of complexes **326** and **327** was investigated in the homodimerization and hydrothiolation of alkynes using 1-hexyne as a model substrate.^196^ In dimerization, **327** with bulkier Dipp wingtip groups proved to be inactive. In contrast, the Mes analogue **326** showed high catalytic activity with excellent selectivity as only the *gem*-enyne isomer is formed in the reaction, with complete conversion in less than an hour with a catalyst loading of 1 mol % at 80 °C (i, Scheme 49). Further experiments with a range of substrates showed **326** to display high functional group tolerance while maintaining the high selectivity. In addition, the complex is stable in atmospheric conditions and does not need a cocatalyst to function. In the hydrothiolation of alkynes, for which the reaction conditions were optimized using 1-hexyne that was hydrothiolated with thiophenol, both complexes **326** and **327** showed catalytic activity at 1 mol % catalyst loading at 80 °C, yielding α-vinyl sulfide with over 90% selectivity (ii, Scheme 49). **326** showed excellent selectivity toward α-vinyl sulfide isomers comparable to the best-known catalysts in the hydrothiolation of aliphatic alkynes with aliphatic thiols. On the other hand, in hydrothiolation involving aryl-substituted substrates, **326** displayed a decrease in catalytic performance.

**326** also showed high catalytic activity and selectivity in the sequential bis-hydrothiolation of two different alkynes to nonsymmetrical bis-α-α′-vinyl sulfides (iii, Scheme 49).^196^ In the reaction catalyzed by **326**, the alkyne was first reacted with a dithiol to afford mono-α-vinyl sulfide, followed by the addition of a different alkyne yielding nonsymmetrical bis-α-α’-vinyl sulfides with high selectivity. For terminal alkynes, the formation of bis-α-vinyl sulfides was observed, while for internal alkynes, the reaction yielded β-*E*-vinyl sulfides. Remarkably, **326** showed catalytic activity even in a one-pot sequential dimerization and hydrothiolation tandem reaction, in which the alkyne dimerization of dimethylaminopropyne was followed by the addition of 1-hexanethiol leading to *syn*-addition of the thiol across the internal alkyne affording the formation of 1,3- and 1,4-*gem*-ene-β-*E*-vinyl sulfides as the main products (iv, Scheme 49).

### 4.2 Electronic Consequences of Wingtip Sterics

**Scheme 50:**
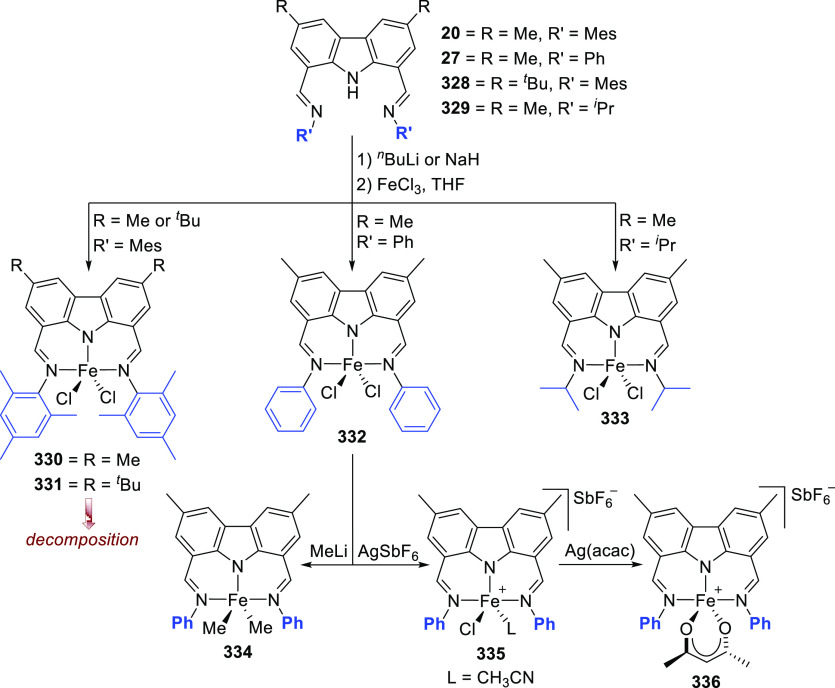
Fe^III^ Complex Stability and Reactivity Effected by Wingtip Steric Bulk

A fine balance is drawn for steric bulk at the wingtip sites, with increasing steric bulk leading to complexes with higher reactivity that can lead to decomposition. Conversely, small wingtip substituents might not provide sufficient steric bulk for stabilizing the complex which could also result to decomposition or unwanted complex formation. This was illustrated by investigating various iron complexes, of which **332** and **333** were reported to be stable even under atmospheric conditions (Scheme 50).^262^ However, Fe^III^ complexes **330** and **331**, featuring increased steric bulk at the imine donor wingtip positions, were found to decompose in air and also exhibited diminished stability in solution while under an inert atmosphere. The tendency of the complexes to decompose was speculated to be a result of the bulky mesitylimino wingtip substituents. The sterically demanding ligand can result to unfavorable interactions between the wingtip substituents and the chloride ligands, possibly resulting to imine decoordination which decreases the stability of the complex. The analogue of **330**, the Fe^II^ complex **21** (Scheme 3), was isolated and reported to be stable. Ligand **27**, with a lower steric demand when compared against the mesityl containing pincer ligands, imparted sufficient stabilization to the iron center allowing for subsequent reactivity studies, further advancing the scope of iron complexes accessible. Reaction of **332** with MeLi lead to the formation and isolation of **334**. Two consecutive chloride abstractions, first with AgSbF_6_ followed by Ag(acac) resulted to **335** and **336**, respectively (Scheme 50).

**Scheme 51:**
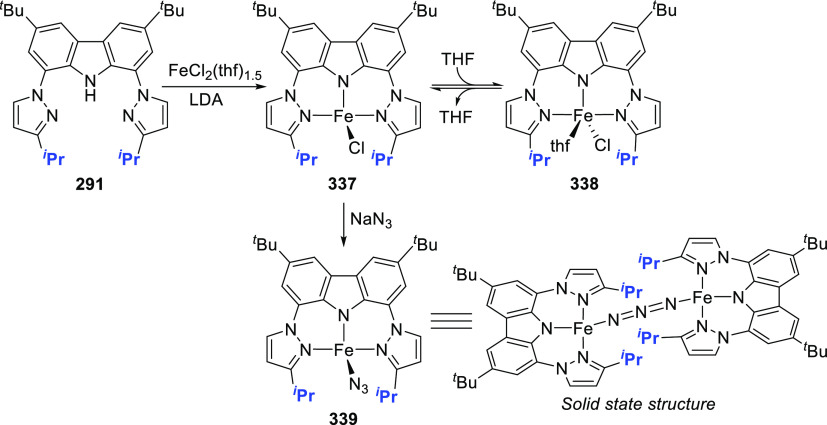
Bis(pyrazole)carbazolide Coordinated Fe^II^ Complexes

Lee et al. furthered their investigation into the bis(pyrazole)carbazolide iron class of compounds (see section 3.3.3), in this case isolating complexes with varying steric bulk at the wingtip positions.^263^ For the Fe^II^ complexes, evidence of the influence that the wingtip sterics exhorts on the complex was noted when instead of the ligand **290** the less sterically hindered ligand **291** was employed, allowing for the formation of iron complexes **337**–**339** in which the metal center is coordinated by all three pincer nitrogen donor atoms. The coordination sphere is completed by chlorido, thf and chloride, or azido coligands (**337**, **338**, and **339**, respectively, Scheme 51), in contrast to **292** (Scheme 43) where the bulky bis(trimethylsilyl)amide coligand accompanies bidentate coordination of the carbazolide ligand.^264^ Slight modulation of steric bulk on the wingtip positions (e.g., changing from *tert*-butyl to isopropyl substituents) can thus similarly control complex reactivity by variation of coordination number, where tridentate coordination increases complex stability at the expense of ligand hemilability.

**Scheme 52:**
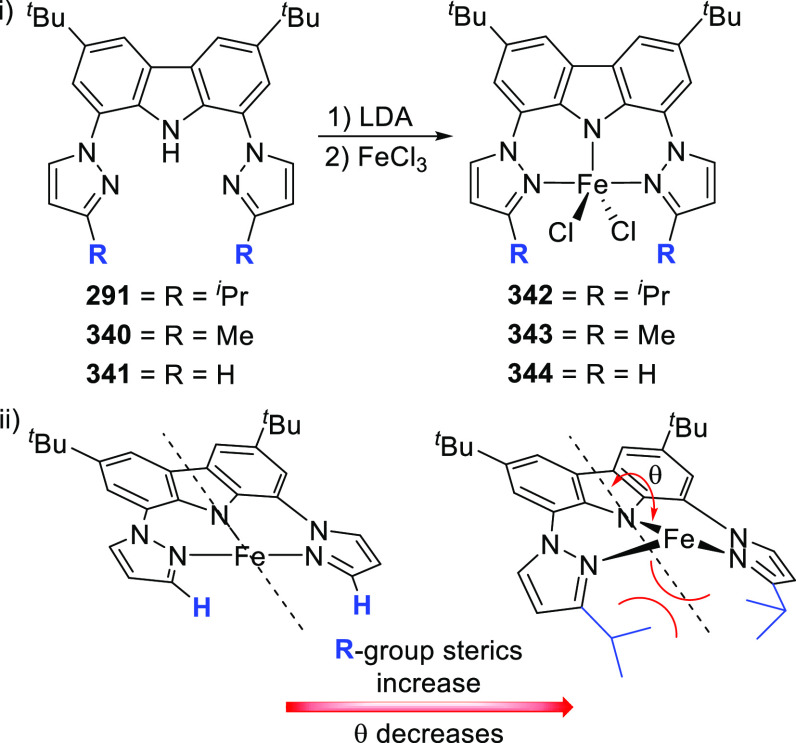
Wingtip Sterics Dictating Iron’s Reactivity

Bis(pyrazole)carbazolide iron(III) complexes **342**–**344** were prepared (i, Scheme 52)^263^ following a similar procedure as for Fe^II^ (Scheme 52)^264^ and Ni^II^ complexes (Scheme 44).^265^ Crystal structure and electrochemical analysis, in addition to theoretical calculations, EPR, Mössbauer, and electronic absorption spectroscopy, again evidenced the influence that the wingtip steric bulk exhorts on the reactivity of the synthesized complexes.^263^ Increasing the steric bulk from H to Me to ^*i*^Pr at the wingtip position leads to an out-of-plane distortion of iron from the plane of the carbazole backbone (ii, Scheme 52). The donor pyrazole groups also have to deviate from planarity in order to accommodate iron’s out-of-plane movement. On the basis of the results obtained, the authors suggested that the out-of-plane deviation decreases the extent of orbital overlap between the carbazole’s nitrogen *p*-orbital and the metal’s *d*-orbital. This could decrease electron donation from the ligand to the metal, reducing the ligand’s overall electronic effect on the complex as manifested in the redox potential of the complexes investigated. The steric bulk at the wingtip position also influences complex formation, as the authors had to prepare **342** at low concentrations to reduce the possible formation of byproducts, one of which was speculated to be an iron complex coordinated by two carbazolide pincers.

**Scheme 53:**
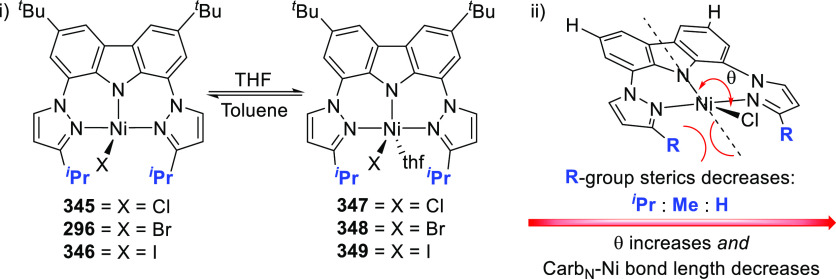
Wingtip Sterics Influencing the Coordination Environment around a Nickel(II) Complex

The series of Ni^II^ complexes coordinated by the bis(pyrazole)carbazolide pincer disclosed by Lee and co-workers was expanded (*vide supra*, section 3.3.3).^265^ The inclusion of bulky isopropyl wingtip substituents at the pyrazole donor groups was rationalized by their effort to access nickel(II) complexes with a higher reactivity compared against the overall kinetically inert square planar nickel analogues. The authors noted the tendency of the wingtip steric bulk of the bis(pyrazole)carbazolide to point toward the fourth coordination site. This ligand design strategy compounds the influence of the wingtip steric bulk on the metal, allowing for increased steric pressure at the metal center and forcing the metal to adopt a geometry higher in energy due to an alteration of the molecular orbital’s energy and electronic population. Thus, complexes **345**, **296**, and **346** isolated after treatment of **291** with LDA followed by the corresponding nickel halide precursor exhibited a seesaw geometry around the four-coordinate metal center and not a square planar coordination environment (i, Scheme 53). Dissolving the complexes in THF resulted in its coordination and formation of the corresponding five-coordinated complexes **347**–**349**. The thf ligand could be removed under reduced pressure followed by dissolving the solid in a noncoordinating solvent. All complexes were determined to be paramagnetic. Theoretical investigation confirmed the paramagnetic nature of the complexes, describing two SOMOs, the *d*_x^2^–y^2^_ and *d*_z^2^_ orbitals, energetically separated from three low-lying filled *d*-orbitals. Calculations further supported the experimentally determined electronic spectra, describing the lowest energy transition to be ligand-to-metal charge transfer (LMCT) with the carbazolide-nitrogen significantly contributing to the ligand π-orbital.^265^ The ligand π-orbital in turn donates into the metal’s *d*_z^2^_-orbital.

Additional calculations on a model complex (the *tert*-butyl groups at the carbazole backbone were omitted during the calculations) evidenced the sought-after influence exhorted by the NNN-pincer ligand’s wingtip sterics on the electronic and coordination properties around the four-coordinate Ni^II^ complexes (ii, Scheme 53).^265^ Increasing the steric bulk of the wingtip substituents resulted in an increase in the steric repulsion between the wingtip substituents and chloride ligand. This in turn allowed for the N(carbazole)–Ni–Cl bond angle to decrease from the ideal square planar angle. The *trans* influence exhorted on the carbazole-nitrogen by the chloride ligand diminishes due to the chloride ligand being forced out of the carbazole plane, resulting in an increase in the N(carbazole)–Ni bond length. Calculations suggested that these changes in both the bond length and bond angle leads to the complex’s singlet state becoming energetically less competitive with the triplet state, favoring the triplet state which would yield a complex higher in reactivity due to the electrons being unpaired. Therefore, manipulating the wingtip steric bulk can have a significant impact on both the electronic and coordination environment around a metal center, affecting its kinetic stability.

**Scheme 54:**
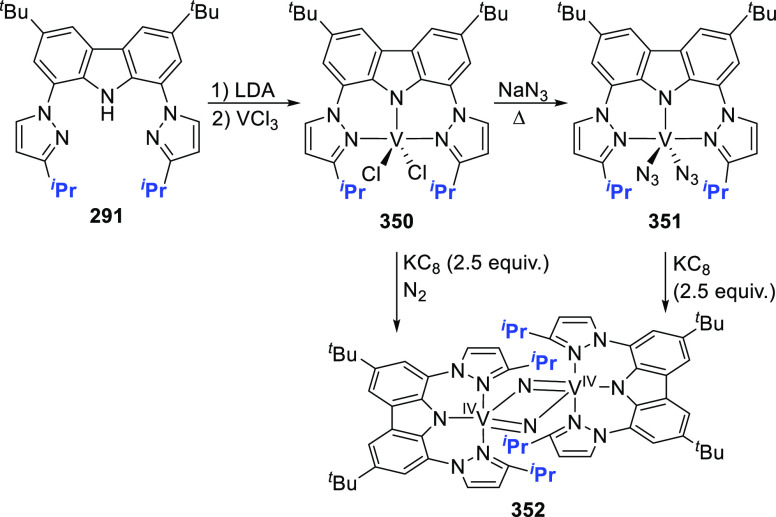
Bis(pyrazole)carbazolide Vanadium Complexes

In an effort to gain a better understanding of the role that vanadium fulfills in vanadium nitrogenase, Lee and co-workers coordinated the bis(pyrazole)carbazolide pincer to vanadium(III).^266^ Similar to the preparation of the corresponding iron^263^ and nickel complexes,^265^**350** could be synthesized by deprotonation of **291** with LDA followed by addition to a slurry of VCl_3_ in THF (Scheme 54).^266^ Reacting the dichloride with excess sodium azide yielded the bis(azido) vanadium complex **351**, which yields the dimeric vanadium(IV)-nitride complex **352** after reduction with potassium graphite. The dimeric nitride **352** could alternatively be prepared through reduction of the dichloro complex **350** with KC_8_ under a nitrogen gas atmosphere. **352** was determined to be highly stable, as evidenced by reactions with various redox, proton, and hydrogen atom transfer reagents. The reactivity studies were corroborated by DFT calculations, attributing the stability of **352** to both steric protection by the ligand’s bulky wingtip substituents and strong metal-nitrogen π-bonds.

### 4.3 Cooperativity of Wingtips for Bond Manipulation

#### 4.3.1 Protic NHC Wingtips

**Scheme 55:**
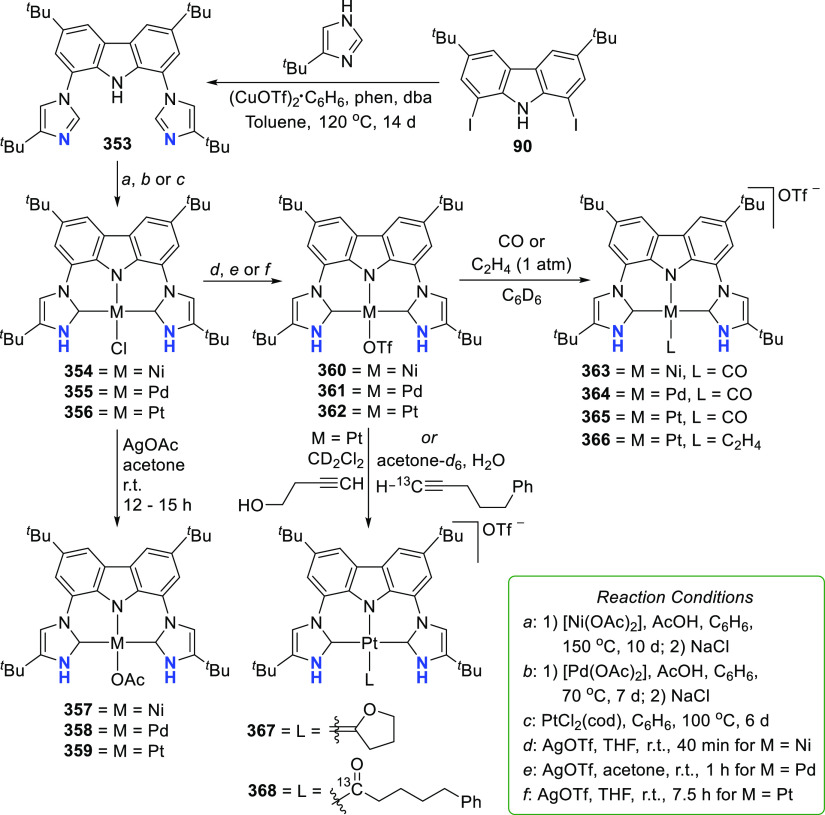
Synthesis of Bis(PNHC)-Carbazole and Its Group 10 Metal Complexes

Implication of protic *N*-heterocyclic carbenes (PNHCs) as ligands for metal-ligand bifunctional cooperative catalysis has increased interest in this class of NHCs, although synthetic access to PNHC complexes are generally considered challenging.^267−269^ Grotjahn et al. introduced PNHCs as flanking groups to bis(imidazol-2-ylidene)carbazole CNC-pincer ligand **353** by a three-step synthesis (Scheme 55) from carbazole,^239^ by modifying the method reported by Kunz et al. for aprotic BIMCA analogues (*vide supra*, section 2.3).^140,190^ Direct metalation of **353** with the group 10 metal (Ni, Pd, and Pt) precursors afforded the formation of two carbon-metal bonds from the unfunctionalized C–H bonds of the PNHC moieties in a single synthetic step (Scheme 55).^239^ The chloride complexes **354**–**356** obtained were further converted into acetate and triflate analogues, **357**–**359** and **360**–**362**, respectively, by using the corresponding silver salts (Scheme 55). NMR and IR spectroscopic investigations indicated intramolecular hydrogen bonding in **357**–**362**. Solid state structures of **358** and **361** confirmed this by revealing interaction between the acetato ligand and one NH wingtip in **358**, while in **361** a simultaneous interaction between the triflato ligand and two NH wingtips were observed.

Triflate complexes **360**–**362** were subjected to reactivity tests with CO and ethylene to yield complexes **363**–**365** and **366**, respectively (Scheme 55).^239^ CO binding was shown to be reversible in the nickel and palladium complexes **363** and **364**, respectively, suggesting a weaker M–CO bond than in the platinum complex **365**. In the case of ethylene, only the formation of Pt complex **366** (Scheme 55) was observed. Spectroscopic and structural studies of **363**–**365** evidenced intermolecular hydrogen bonding between the uncoordinated triflate anion and two NH wingtips. Further reactivity tests were performed on **362**, which showed *anti*-Markovnikov selectivity in O–H addition to alkynes. Stoichiometric reaction of **362** with 3-butyn-1-ol resulted in the formation of **367** featuring a cyclic carbene as a coligand, while the reaction with ^13^C-labeled alkyne yielded the acyl complex **368** (Scheme 55).

**Scheme 56:**
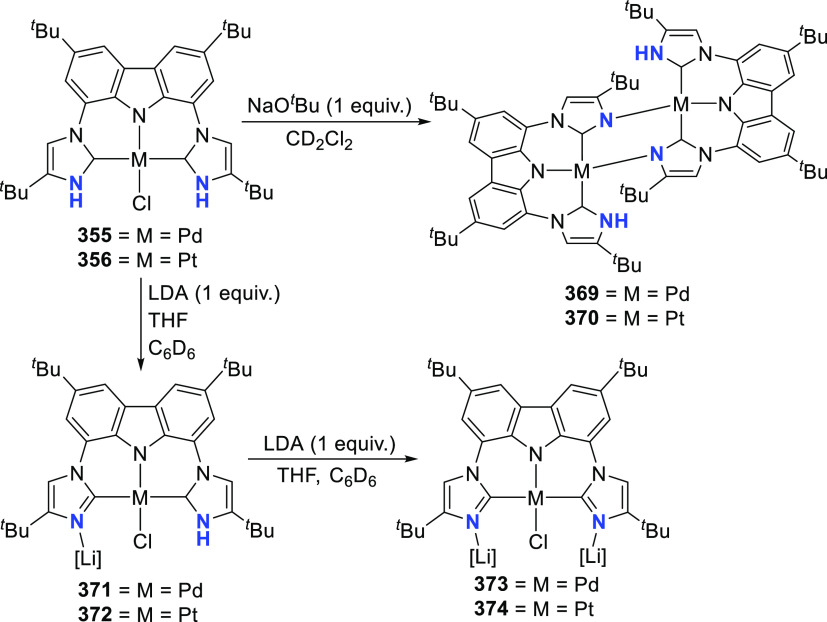
Formation of Dimers and Mono and Di-Lithium Adducts of Bis(PNHC)-Carbazolide Group 10 Metal Complexes

With the chloride complexes **355** and **356**, Grotjahn and his group targeted partial deprotonation of the ligand to obtain complexes that simultaneously contain both the bond-activating imidazol-2-ylidene unit and the PNHC proton donor.^270^ For this, they first treated **355** and **356** with sodium *tert*-butoxide in dichloromethane, which instead of the targeted monodeprotonation led to the formation of the dimers **369** and **370** (Scheme 56). The crystal structure of **369** showed a dimeric structure consisting of two metal complexes coordinated to each other via the nitrogen atoms of the imidazolyl moieties following deprotonation of the NH wingtips. Dimer formation was presumably due to the loss of NaCl. Hence, the reaction conditions were adjusted and **355** and **356** were treated with a stronger base LiN(^*i*^Pr)_2_ in the more polar solvent THF. One base equivalent in the reactions resulted in the formation of **371** and **372** (Scheme 56), in which one of the PNHC moieties was deprotonated. The addition of the second base equivalent led to the deprotonation of the second NH wingtip to yield the bis(imidazolyl) complexes **373** and **374** (Scheme 56). Dimer formation was not observed in these reactions and prompted reactivity tests with H_2_, ethylene, and 1-heptene. However, the substrates did not displace the chlorido ligand without dimer formation, which appears to dominate the reactivity of the bis-PNHC complexes under basic conditions.

#### 4.3.2 Tethered NHC Wingtips

**Scheme 57:**
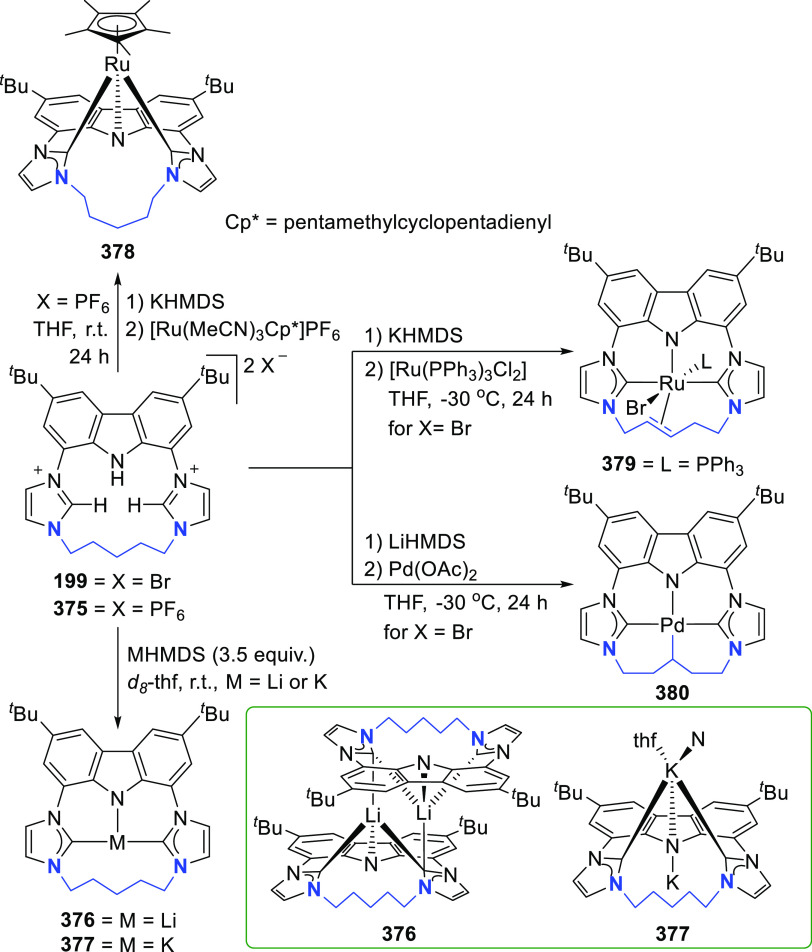
Synthesis of Ruthenium(II) and Palladium(II) Complexes Bearing a Macrocyclic CNC Ligand

Modification of the BIMCA ligand was done to include a pentamethylene tether connecting the two NHC donors in the ligand precursors **199** or **375** with bromide or PF_6_^–^ counterions, respectively (Scheme 57).^134^ If the deprotonated precursor **375** was coordinated to a pentamethylcyclopentadienyl (Cp*) ruthenium(II) precursor, a rare example of the carbazole-based pincer ligand with facial coordination in complex **378** is obtained, similar to the RuCp* complex of the BIMCA ligand with homoallyl wingtip groups (*vide infra*, section 4.3.3).^271^ Besides the presence of the coligand Cp*, the hindered rotation of the carbene moieties in the tethered pincer ligand were concluded to enable the observed *fac* coordination of the BIMCA ligand, contrary to the conventional *mer* geometry as demonstrated for example, by a bis(triazolylidene)carbazolide ruthenium(II) complex.^272^ Not only the *d*^6^-metal but also the alkali metal complexes **376** and **377**, obtained from the reaction of the protioligand **199** or **375** with LiHMDS or KHMDS, respectively, display this *fac*-geometry (Scheme 57).^134^ Crystal structure analysis of the lithium complex **376** revealed a dimeric structure in the solid state in which the coordination of the ligand can be described as both chelating and bridging, as the ligand is coordinated to lithium via the carbazolide-nitrogen atom and two NHC donor moieties, one of which also coordinates the lithium atom of the other lithium pincer complex monomer to form the observed dimer. In contrast, in the case of the potassium complex **377**, the solid-state structure showed that the BIMCA ligand coordinates to potassium only in a facial manner. The NHC moieties of the ligand coordinate to potassium while the nitrogen of the carbazolide spacer coordinates to the potassium of the next monomeric complex, thus forming a polymeric structure.

The properties of transition metal complexes of macrocyclic CNC ligands containing alkyl tethers of 8–16 C atoms have been found to depend on ring size, even though the tether itself shows no reactivity.^273−278^ It was anticipated that the smaller ring size macrocyclic BIMCA ligand derivative with a pentamethyl tether could induce a stronger interaction with the metal due to the proximity and influence its reactivity by steric restrictions when coordinated in traditional meridional geometry.^134^ Indeed, the ruthenium complex **379** obtained from the reaction of *in situ* deprotonated bis(imidazolium) salt **199** with [Ru(PPh_3_)_3_Cl_2_] showed that intramolecular C–H activation occurred resulting in the formation of an olefinic donor site (Scheme 57), with the pentamethylene-tethered BIMCA ligand acting as a monoanionic tetradentate macrocyclic ligand. Another proof for intramolecular C–H activation induced by the proximity of the alkyl tether to the metal center was provided in the form of a palladium complex **380** from the reaction of *in situ* deprotonated **199** with Pd(OAc)_2_ (Scheme 57). As was shown by NMR experiments, intramolecular C–H activation led to the deprotonation of the alkyl chain and the formation of a carbanionic donor site, resulting in the formation of a dianionic tetradentate macrocyclic ligand. The observed C–H activation was rationalized by the proximity of pentamethylene in the first formed CNC pincer complex, which enables its intramolecular deprotonation by the acetate ligand.

#### 4.3.3 Hemilabile Allyl-Functionalized NHC Wingtips

**Scheme 58:**
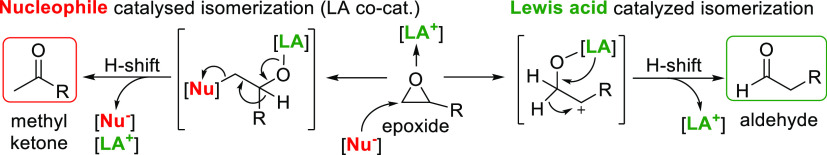
Nucleophilic and Lewis Acid Catalyzed Isomerization of Terminal Epoxides

The influence of not only the **L** groups but also the **R** wingtips on the electronic environment is well illustrated by the modification of the flanking NHC donors’ wingtips from methyl to coordinating allyl groups, in the rhodium complexes of BIMCA.^229,279^ Increased control of the rhodium coordination sphere is hereby obtained and allows not only for increased electron density at the metal (by replacement of the carbonyl ligand in **194** (section 3.2 and Scheme 30) with a coordinating allyl moiety) but also for ligand-assisted metal reactivity in catalytic conversions. The additional nucleophilicity of the rhodium complexes was exploited to improve catalytic performance in the isomerization of terminal epoxides to methyl ketones. The rearrangement of epoxides, the so-called Meinwald reaction,^280^ is most commonly catalyzed by Lewis acids leading to the formation of aldehydes,^281−293^ whereas rarer examples of catalytic isomerization involve the use of a nucleophilic catalyst together with a Lewis acid cocatalyst to yield methyl ketones (Scheme 58).^294−298^

**Scheme 59:**
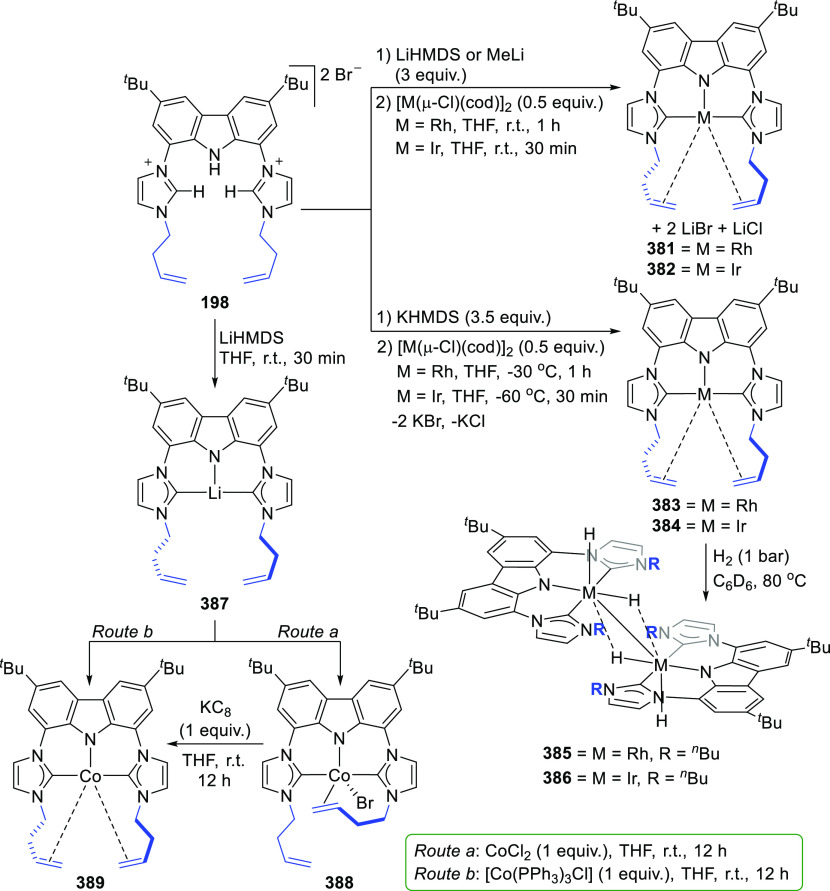
Synthesis of Rhodium, Iridium, And Cobalt Complexes of *N*-Homoallyl-substituted Bis(NHC) Pincer Ligand

Rhodium complex **194** (section 3.2) was shown to catalyze the regioselective rearrangement of terminal alkyl epoxides into methyl ketones under mild conditions, i.e., in a reaction that was carried out in the presence of 5 mol % of **194** along with 20 mol % of the Lewis acid additive LiNTf_2_ at 60 °C in benzene-*d*_6_.^229^ Near to full conversion of monoalkylated epoxides into their respective methyl ketones with no detection of the respective aldehydes was achieved. Further studies with other terminal epoxide derivatives showed a high functional group tolerance for a wide variety of substrates with different functionalities; however, a notable decrease in conversion and/or yield was observed.^229,279^ To improve the efficiency of the catalyst, attention was shifted to the *N*-homoallyl substituted ligand **198** to access a rhodium complex that is CO-free, thereby increasing the electron density at the metal center, while the olefinic units coordinated to the rhodium center stabilizes the complex intramolecularly during catalysis, thus increasing its lifetime.^279^ For this, *in situ* deprotonation of bis(imidazolium) salt **198** with KHMDS or LiHMDS, followed by transmetalation with [Rh(μ-Cl)(cod)]_2_, afforded **383** or **381**, respectively, the latter of which was isolated with lithium salts, LiBr and LiCl (Scheme 59). The syntheses of the analogous cobalt and iridium complexes were also reported (Scheme 59).^299^ Iridium complexes **384** and **382** were obtained by following the synthetic method of Rh complexes **383** and **381**. For the preparation of the cobalt complex **389**, two methods were reported: both direct transmetalation (Scheme 59, route b) and a two-step synthesis (Scheme 59, route a), where the transmetalation was followed by the reduction of the formed Co^II^ complex **388** to the target complex **389**.

The catalytic activity of rhodium complexes **383** and **381** was further tested in the isomerization of terminal epoxides.^279^ For **381** including LiBr and LiCl salts, full conversion into methyl ketones was observed. The lithium salt free **383**, on the other hand, did not catalyze the rearrangement, indicating that a weak Lewis acid cocatalyst in the reaction is necessary for catalytic activity. **381** proved to be catalytically more active than **194**, catalyzing the nucleophilic Meinwald reaction at a lower temperature and in the absence of an additional Lewis acid. With a catalyst loading of 5 mol % at room temperature in benzene-*d*_6_, **381** catalyzed the isomerization of various functionalized terminal epoxides into the respective methyl ketones almost quantitatively and with full regioselectivity, thus showing a high functional group tolerance. Even for aryl oxiranes, almost full conversion into methyl ketones was achieved with excellent chemo- and regioselectivity, thus emphasizing the crucial role of the higher nucleophilicity of the complex **381** in the rearrangement reaction. In contrast, the iridium and cobalt complexes **384** and **389**, respectively, showed lower catalytic activity than their Rh analogue **383** in the presence of Lewis acid additive LiBr.^299^ The lower catalytic activity of the iridium and cobalt complexes was justified by the differences in their oxidation potentials compared to that of **383**. In the case of **389**, the lower oxidation potential is less favorable toward the nucleophilic opening of the epoxide ring, decreasing the reactivity of the compound. On the other hand, the oxidation potential of **384** is higher than that of **383** leading to an increasing metallacyclopropane character, translating to higher stability observed for the coordinated *N*-homoallyl moieties. This hampers the dissociation of the second allyl moiety, which is a prerequisite for the formation of a nucleophilic active species, thus reducing the activity of complex **384**. However, **389** and **384** showed increased catalytic activation under H_2_ gas (1 bar), although it also led to side reactions. To evaluate whether *N*-homoallyl moieties are susceptible to hydrogenation under catalytic conditions, additional hydrogenation experiments were performed for the iridium and cobalt complexes **384** and **389**, respectively, but also for the rhodium complex **383** by exposing them to H_2_ gas.^299^ In the case of rhodium and iridium, complete hydrogenation of *N*-homoallyl moieties into *N*-*n*-butyl groups and the formation of dimeric hydrido complexes **385** and **386** (Scheme 59) were observed, as verified by single crystal X-ray structure analysis.

**Scheme 60:**
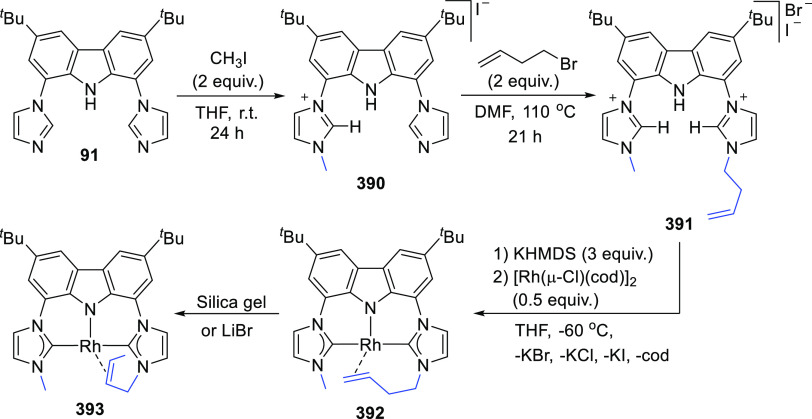
Synthesis of Unsymmetrically *N*-Homoallyl-Substituted BIMCA Ligand and Its Rhodium Complexes

Capitalizing on an even more nucleophilic metal center to yield a more active catalyst, the catalytic system could be further improved by introducing the 16-electron rhodium complex **392** with only one *N*-homoallyl substituent in the ligand scaffold (Scheme 60).^300^ The ligand precursor **391** was synthesized by selective monoalkylation of **91** with methyl iodide followed by *N*-allylation to afford the targeted bis(imidazolium) salt **391** (Scheme 60). *In situ* deprotonation and subsequent transmetalation of **391** produced the rhodium complex **392**. Attempts to isolate **392** by chromatography unexpectedly led to the isomerization of the homoallyl double bond and the formation of compound **393**, which was confirmed by NMR spectroscopy. **392** catalyzed the isomerization of aryl oxiranes to the corresponding methyl ketones in high yields with excellent chemo- and regioselectivity at room temperature with lower catalyst loading (1 mol %) and shorter reaction time, thus surpassing **381** in terms of catalytic activity. Moreover, **392** also showed excellent functional group tolerance for a wide range of epoxides. However, the reaction still needed a Lewis acid additive to preactivate the substrate.

**Scheme 61:**
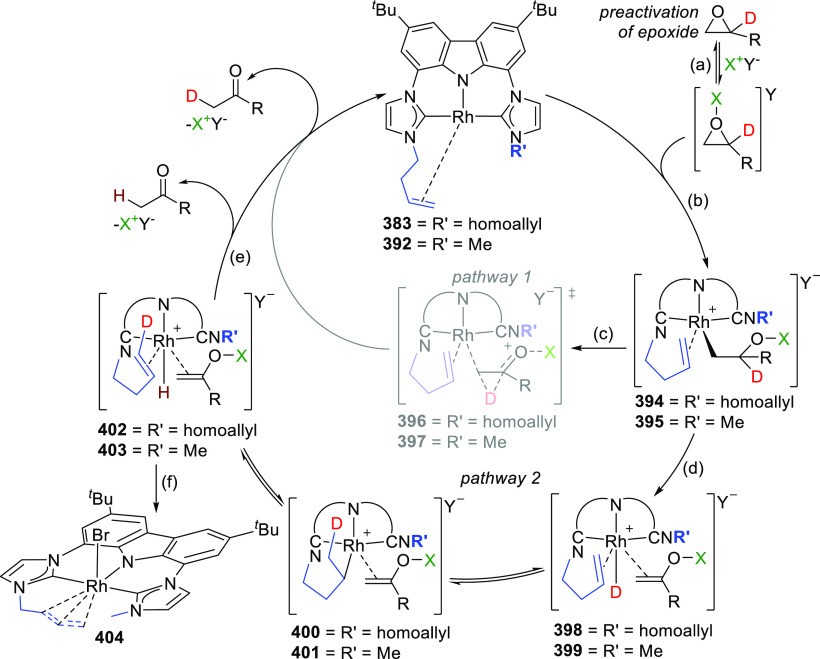
Proposed Catalytic Cycle for **383** and **392**, Demonstrating Catalyst Deactivation Product Formation

A possible catalytic cycle was proposed for the Meinwald isomerization of epoxides catalyzed by nucleophilic rhodium complexes **383** and **392** (Scheme 61).^300^ In the first step (a), the Lewis acid cocatalyst preactivates the epoxide substrate by coordination. In the case of catalyst **383**, the dissociation of the second homoallyl group is a prerequisite for the formation of the catalytically active complex. This is followed by a nucleophilic attack of the Rh^I^ center on the most electrophilic and usually the least substituted site of the epoxide ring (step b), resulting in the opening of the epoxide ring and the formation of the Rh^III^ intermediate **394** or **395**. From this point forward, intermediate **394** or **395** can react in two different reaction pathways. In pathway 1, the intermediate **394** or **395** can release the methyl ketone directly by a concerted 1,2-hydride transfer proceeding through the transition state **396** or **397**, respectively. Alternative reaction pathway 2 involves β-hydride migration (step d) leading to the formation of the Rh^III^ hydrido complex **398** or **399**, followed by reductive elimination (step e) under which the methyl ketone is released and the catalytically active Rh(I) complex **383** or **392** is regenerated. Formation of the Rh^III^ hydrido complex **398** or **399** could not be verified in practice.

Further insight into the mechanistic details of the catalytic cycle was obtained by D/H exchange experiments.^300^ D/H exchange was observed in experiments carried out under catalytic and stoichiometric conditions with the deuterated phenyloxirane substrate and *N*-homoallyl-substituted **381** (containing lithium salts, see Scheme 59) and **392**, as in all cases the deuterium had been replaced by hydrogen in 5–7% of the formed methyl ketones. This confirmed that the reaction pathway 2 is more plausible for the rhodium catalysts and the homoallyl substituent of the ligand plays a role in the catalytic cycle, inserting into intermediate **398** or **399** to form a rhodium alkyl intermediate **400** or **401** which can further undergo D/H exchange if a deuterated substrate is present in the reaction.

In connection with the isomerization of epoxides catalyzed by **392**, the complex **404** was also isolated, which is the probable deactivation product of the catalyst (Scheme 61).^300^ The crystal structure of the compound showed the formation of a bromido-Rh^III^ complex **404** where the *N*-homoallyl substituent is η^3^-allyl coordinated to the metal center. It was speculated that **404** was formed after catalysis and is a result of the C–H activation of the allylic position yielding an allylhydride complex followed by replacement of the hydride with a bromido ligand.

**Scheme 62:**
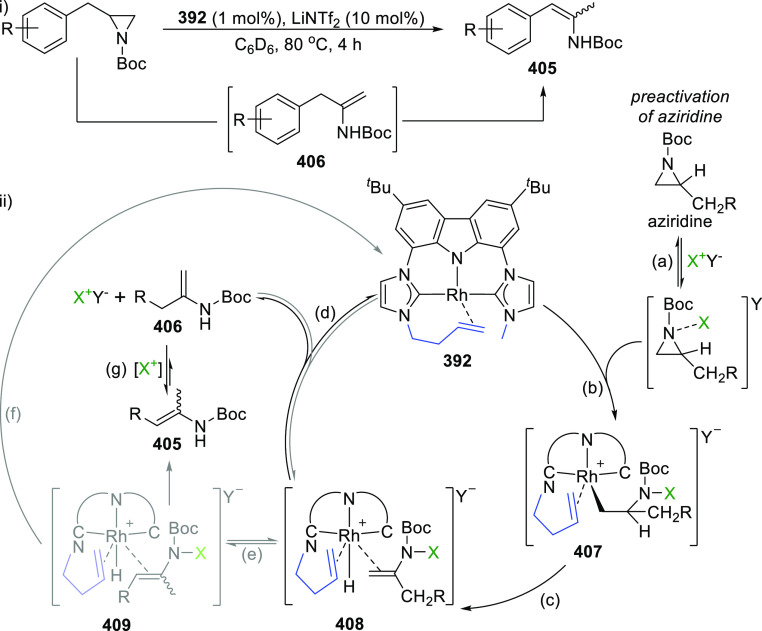
(i) Catalyzed Isomerization of *N*-Boc Terminal Aziridines and (ii) Proposed Mechanism for the Isomerization of Terminal Aziridines Catalyzed by **392**

The catalytic activity of nucleophilic rhodium complexes was applied also in the selective isomerization of terminal aziridines (analogues of epoxides) to enamides.^301^ As before, the most nucleophilic complex **392** exceeded the catalytic performance of **194** and **383**, while a Lewis acid cocatalyst remains essential. **392** catalyzes the transformation of various *N*-Boc terminal aziridines into the corresponding enamides at 1 mol % catalyst loading (i, Scheme 62). Isomerization of aziridines with electron-donating substituents was found to proceed faster than aziridines with electron-withdrawing substituents. In these cases, the formation of an intermediate **406** with a terminal C=C double bond was observed, which eventually rearranged to the target enamide.

A mechanism analogous to the nucleophilic dual-activation pathway proposed for the rearrangement of epoxides was also proposed for the isomerization of aziridines (ii, Scheme 62).^301^ In the first step (a), the Lewis acid coordinates to the aziridine, preactivating it. In the next step (b), the nucleophilic attack of the catalyst **392** on the most electrophilic site of the aziridine results in the opening of the aziridine ring and the formation of an intermediate **407** that is probably Lewis acid stabilized. The following β-hydride elimination (step c) can lead to the formation of the Rh^III^ complex **408**, which in the subsequent reductive elimination (step d) reforms the catalytically active compound **392** and releases the intermediate **406**. This further isomerizes to the target enamide **405** (step e), but it is noteworthy that the rate of this isomerization is substrate-dependent. If aziridine has electron-donating substituents, the intermediate hydrido complex **408** can also isomerize to intermediate **409** (step f), which in a further reductive elimination (step g) releases the target enamide **405** and reconstitutes the active catalyst **392**.

### 4.4 Introducing Chiral Peripheries at the Wingtip Positions

**Scheme 63:**
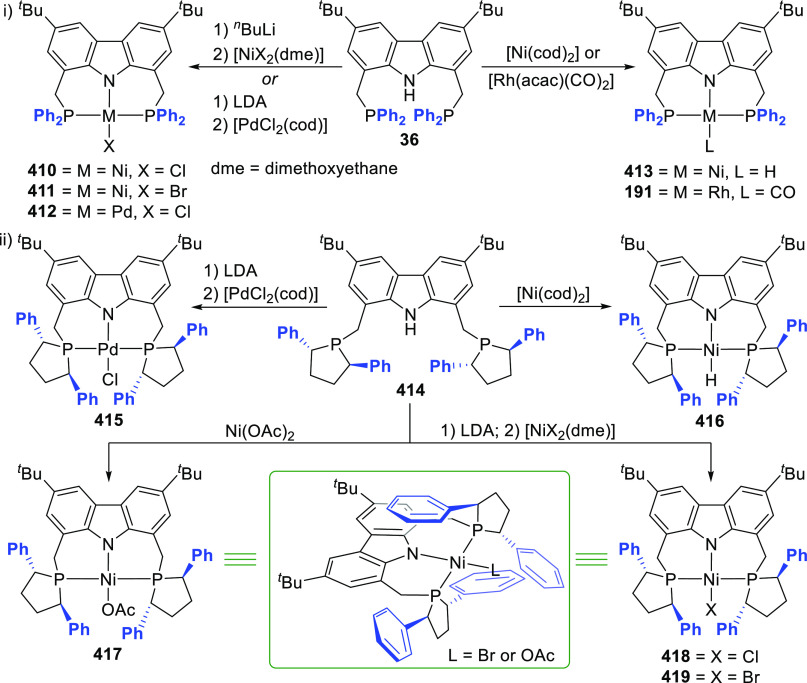
Synthesis and Coordination Chemistry of Achiral and Chiral PNP-Carbazolide Pincer Ligands

Surprisingly, the inclusion of chiral wingtip groups on molecular catalysts for asymmetric transformations has been scarcely explored for monoanionic PNP pincer ligands and is limited to pincer scaffolds with an aliphatic backbone.^302,303^ A report by Gade et al. describes the incorporation of chiral 1,3-diphenylphospholane donor moieties with methylene bridges linking to the carbazole backbone.^154^ The dibromomethylcarbazole **34** is the key intermediate to both the achiral **36** and chiral **414** protioligands, the latter of which is obtained by treatment of **34** with the enantiopure lithium (2*R*,5*R*)-2,5-diphenylphospholanide-borane complex,^304^ followed by deprotection with diethylamine. The position and orientation of the phosphine units render these tridentate ligands as ideal for meridional coordination to *d*-block metals, and the coordination chemistry of a range of transition metals to both achiral **36** (i, Scheme 63) and chiral **414** (ii, Scheme 63) was explored.^154^ Deprotonation of **36** with a lithium base followed by metalation with either [PdCl_2_(cod)] or [NiX_2_(dme)] (dme = dimethoxyethane) yielded the square planar complexes **412** or **410** and **411**, respectively (i, Scheme 63). A similar treatment of **414**, with chiral phospholane wingtips, afforded complexes **415**, **418**, and **419** (ii, Scheme 63). Alternatively, oxidative addition of the carbazole N–H to a Ni^0^ precursor yields the nickel(II) hydrido complexes **413** (i, Scheme 63) and **416** (ii, Scheme 63), with distorted square planar geometry as evidenced by the N(carbazolide)–Ni–H bond angle of 169(1)° and P–Ni–P bond angles 164.12 (3)° from the crystal structure of **413**. The use of acac or acetato metal precursors that can act as an internal base is an alternative route for complex formation, whereby reaction of [Rh(acac)(CO)_2_] with **36** results in formation of **190** (i, Scheme 63) and treatment of **414** with Ni(OAc)_2_ yields **417** (ii, Scheme 63). The effect of the chiral wingtip groups is most pronounced in the molecular structures obtained for the bromido-substituted **419** and acetato-complex **417**. The square planar nickel center is shielded by one phenyl group of each phospholane which are facing in opposite directions, with the coordination plane twisted with respect to the carbazole ring. The two phospholane groups point to opposite faces of the carbazole plane in **419**. This helical twist between the carbazolide ring and the plane spanned by the PNP-ligating atoms is slightly larger in **417**, inferring that the approach of substrates to the metal center can be manipulated by the chiral wingtip groups, as demonstrated for NNN-pincer carbazolide complexes discussed below.

The carbazole backbone forms the basis of the well-known bis(oxazolinyl)carbazole ligand,^305^ which garnered success in the chromium-catalyzed Nozaki-Hiyama reaction^306^ due to the high selectivity imposed by the ligand on the catalyzed reaction.^99,100,305,307,308^ The C–C bond forming reaction is catalyzed by the *in situ* formed bis(oxazolinyl)carbazolide chromium complex and various cocatalyst and/or additives. Indeed, the asymmetric allylation,^309,310^ allenylation,^311^ silylallenylation,^312,313^ and propargylation^314^ of aldehydes can be catalyzed with the *in situ* formed NNN-pincer ligated chromium complex yielding products with high enantiomeric excess and high yields. Furthermore, various other reactions can also be catalyzed with the *in situ* formed bis(oxazolinyl)carbazolide chromium complex, including among others the selective functionalization of halide-substituted olefins with aldehydes or ketones, respectively, yielding the corresponding secondary^315−317^ or tertiary^318^ alcohols, with high enantioselectivities. Access to targeted compounds with excellent selectivity renders this chromium-catalyzed protocol with high relevance, justifying the segments in various reviews regarding the use of the bis(oxazolinyl)carbazole ligand in the Nozaki-Hiyama reaction.^99,100,305,307,308,319,320^ Hence, the topic will only be highlighted in this communication with the inclusion of selected examples illustrating the synthetic potential of the catalytic reaction when catalyzed with the *in situ* formed bis(oxazolinyl)carbazolide chromium complex.

**Scheme 64:**
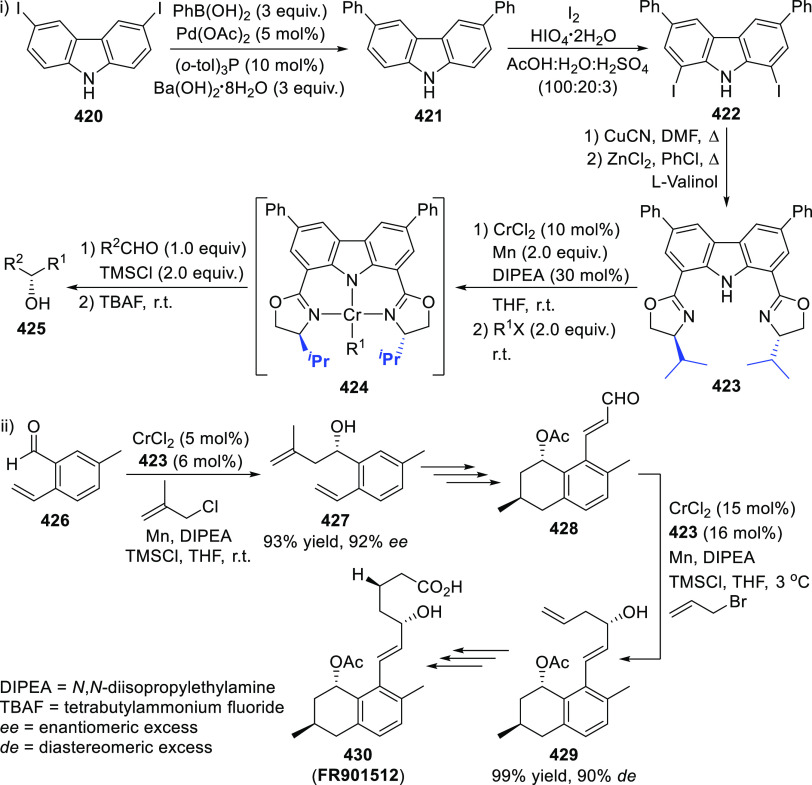
(i) Chiral Carbazole Ligand for Asymmetric Nozaki-Hiyama Allylation and (ii) Preparation of HMG-CoA Reductase Inhibitor FR901512

In a continuation of the work by Fürstner et al.,^321,322^ Nakada and co-workers sought to decrease the stoichiometric use of chromium in the Nozaki-Hiyama C–C bond-forming reaction to catalytic amounts.^305,309,310^ The authors accomplished this by using the bis(oxazolinyl)carbazole ligand **423**, which was synthesized from l-valinol in the presence of ZnCl_2_ and the *in situ* prepared 1,8-dicyano-3,6-diphenylcarbazole, obtained through reacting **422** with CuCN in DMF (i, Scheme 64).^309,310^ Coordination of the NNN-pincer ligand to chromium leads to a stable, yet coordinatively unsaturated complex able to accommodate an incoming substrate. These attributes allowed for catalytically active chromium complex **424** to mediate the asymmetric allylation of aldehydes, with high enantiomeric excess (i, Scheme 64). It was further demonstrated that the ligand could be recycled several times, with retention of selectivity and catalytic activity.^309^ The same group further demonstrated the underlying potential of this synthetic protocol while preparing FR901512 (**430**, ii, Scheme 64), an effective HMG-CoA reductase inhibitor.^323^ The optimized chromium catalyzed Nozaki-Hiyama reaction was exploited en route to **430**, yielding both **427** and **429** from the allylation of aldehydes **426** and **428**, respectively, with high yields and excellent selectivities. Nakada et al. proved the applicability of the catalyzed reaction through the efficient, concise, and protecting-group-free enantioselective synthesis of biologically relevant compounds, while further modification to the ligand’s steric map or the reaction conditions added to the scope of this highly selective transformation protocol (*vide infra*).

**Figure 6:**
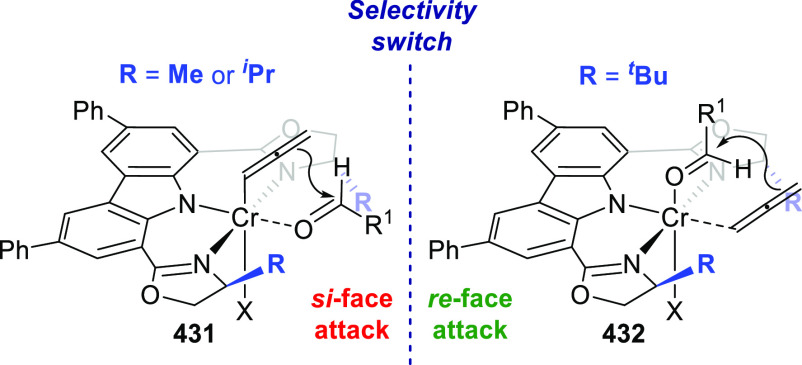
Wingtip sterics dictating selectivity by directing *re*- or *si*-face reactivity.

Turning their attention to the optimization of the selectivity of the asymmetric Nozaki-Hiyama reaction, Nakada et al. leveraged the steric handle provided by the modulation of the ligand’s wingtips. Facile tailoring of the wingtip moieties is paramount to the formation of a stable yet catalytically active complex that can dictate the selectivity for the catalyzed reaction.^305,314^ As such, the decreased steric bulk at **431**, featuring either a methyl or isopropyl wingtip substituent, allowed for coordination of the aldehyde at the equatorial position, leading to *si*-face reactivity (Figure 6). Increasing the steric bulk at the wingtips from methyl and isopropyl to *tert*-butyl groups at **432** directs coordination of the aldehyde at the apical position due to increased steric strain between the wingtip substituents and the incoming aldehyde. With aldehyde coordination at the apical position, *re*-face reactivity ensues and leads to reversal of the observed selectivity. Figure 6 depicts the two different proposed transition states, leading to *si*- or *re*-face reactivity.

**Scheme 65:**
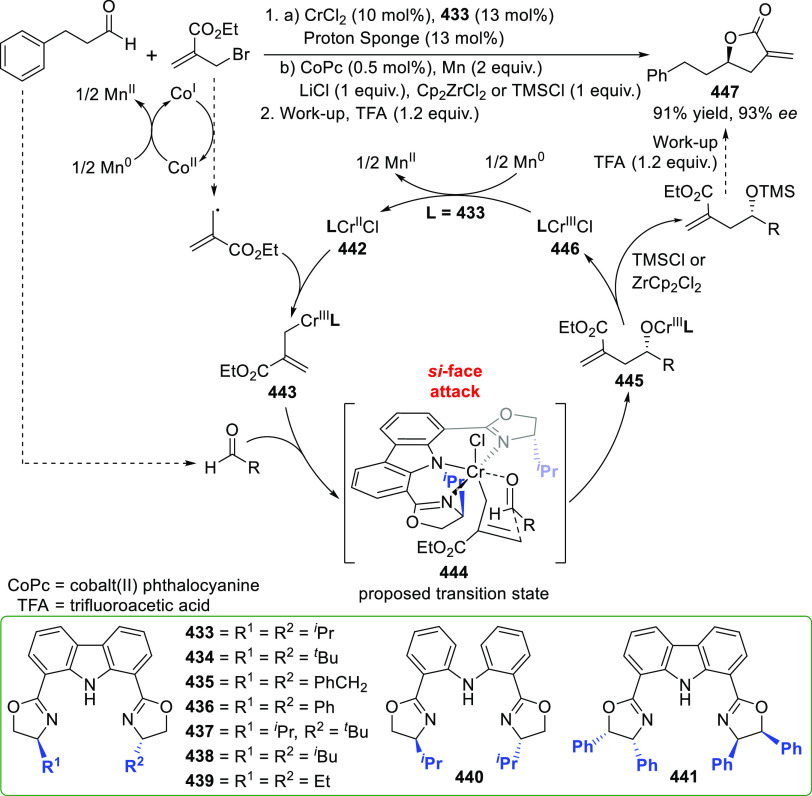
Enantioselective Chromium-Catalyzed Aldehyde Functionalization to Lactones

**Scheme 66:**
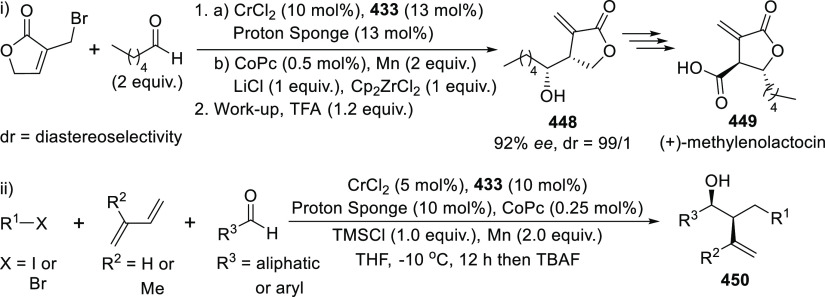
Enantioenriched (i) Lactones and (ii) Homoallylic Alcohols

Enantioenriched α-*exo*-methylene-γ-butyrolactones, structural motifs commonly encountered in natural products, can be accessed through asymmetric chromium-catalyzed allylation of aldehydes followed by lactonization.^324^ The lactones were prepared from the selective chromium-catalyzed allylation of the aldehyde with an acrylate, followed by treatment of the resulting homoallylic alcohol with trifluoroacetic acid leading to the targeted lactone. Ligand fine-tuning through facile steric modification from **423** and **433**–**437** yielded **447** with increased *ee* when employing ligand **433** in the catalytic process, while the requirement for the rigid carbazole backbone was evidenced when compared against the bis(oxazolinylphenyl)amine (BOPA) **440** (Scheme 65). Even though the *ee* of the catalyzed reaction was excellent when using ligand **433**, the yield was unsatisfactory. However, optimization of the reaction conditions improved the catalytic activity, while the *ee* of the reaction remained unchanged. The rate of the reaction could be increased through the addition of cobalt(II) phthalocyanine (CoPc), which facilitates the formation of an allyl radical that coordinates the chromium carbazolide **442**. The rate of transmetalation to the carbazolide chromium complex could also be increased, in this case by the addition of LiCl. Aldehyde coordination at **443** ensues, leading to the alkoxide **445** via the proposed transition state **444** (Scheme 65). Dissociation of the allenic alkoxide from **445** was favored by the addition of ZrCp_2_Cl_2_ or TMSCl. As a proof of concept, the synthesis of (*+*)-methylenolactocin was successfully explored, yielding **449** with a high degree of selectivity (i, Scheme 66).^324^

Enantioselective three-component coupling of 1,3-butadienes with aldehydes and alkyl halides (ii, Scheme 66), either fluorinated or nonfluorinated, was also catalyzed employing similar reaction conditions as used toward **447** (Scheme 65).^325^ Various ligands were used during the catalytic preparation of the homoallylic alcohol, with ligand **433** again demonstrating superior selectivity toward **450** when compared against **435**, **436**, and **438**–**441**. A similar reaction mechanism was suggested, with the notable difference being the incorporation of butadiene, which reacts first with the alkyl radical leading to a π-allyl radical species. The allyl radical is trapped by the Cr^II^ intermediate with subsequent isomerization and carbonyl coordination. The reaction then ensues as above, via a transition state proposed to be similar to **444**. This further demonstrates the selectivity and the broad substrate scope compatibility of the finely tuned bis(oxazolinyl)carbazole ligand in conjunction with the optimized reaction conditions.

**Scheme 67:**
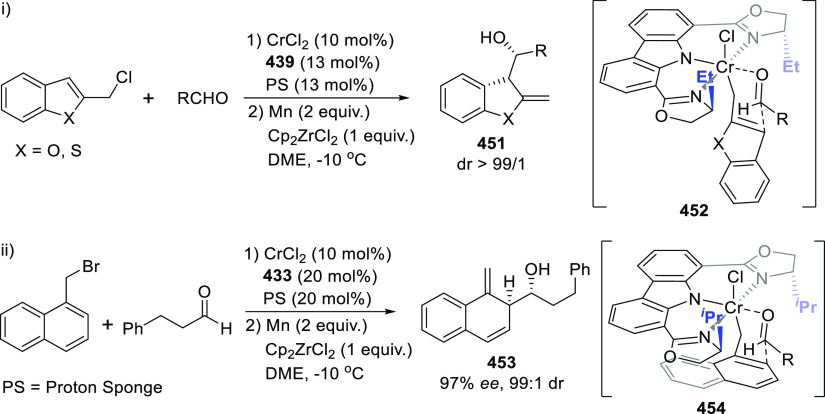
Catalytic Dearomatization of (i and ii) Aromatic Substrates with Aldehydes

The catalyzed dearomatization of aromatic substrates with aldehydes is also feasible with chromium salts, proton sponge (PS), manganese, and ZrCp_2_Cl_2_ (Scheme 67).^326^ Not surprising then is the addition of a bis(oxazolinyl)carbazole ligand that significantly improves the yield and selectivity of this catalyzed transformation. Variation of the steric bulk at the wingtip provides significant control over the catalyzed process. High *ee* was reported when using ethyl-substituted ligand **439**, with a slight decrease in *ee* noted when increasing the wingtip bulk to the ^*i*^Pr moieties of **433**. Further increasing the wingtip steric bulk with ^*t*^Bu groups resulted in reduced yield and selectivities, while the benzyl-substituted pincer ligand **435** did not fare a lot better. The catalytic strategy was extended to include bromomethylnaphthalenes leading to optically pure products with multiple stereogenic centers (ii, Scheme 67).^327^ Similar to the results obtained for heteroarenes,^326^ the catalyzed reaction with ligand **433** furnished the desired products with remarkable enantio- and diastereoselectivities, allowing access to optically pure compounds primed for further manipulation leading to more elaborate molecules.^327^ For both catalyzed processes, a transition state was proposed, as depicted by **452** and **454** in Scheme 67.^326,327^

**Scheme 68:**
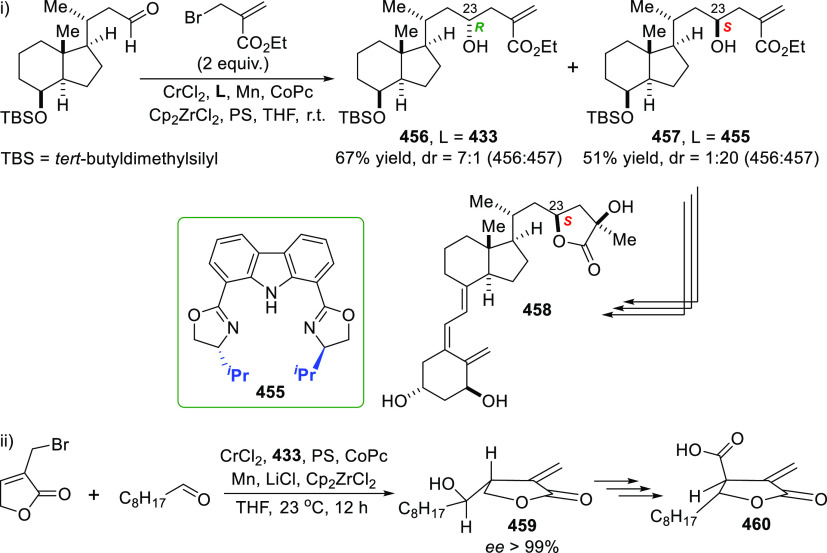
Synthesis of (i) Calcitriol Lactone and (ii) Paraconic Acids

The reaction conditions described by Zhang et al.^324^ were further implemented by the group of Nagasawa during the preparation of calcitriol lactone, a major metabolite of vitamin D_3_.^328^ Under the prescribed reaction conditions with ligand **433**, stereochemistry at C23 was introduced by crotylation of aldehyde with 2-(bromomethyl)acrylate, yielding the corresponding homoallylic alcohol **456** with a 7:1 diastereomeric ratio. The impact of the wingtip substituents was demonstrated when accessing homoallylic alcohol **457**, simply by inverting the stereochemistry at the wingtips of ligand **433** to **455** (i, Scheme 68). Paraconic acids, structural motifs widely encountered in natural products and medicinally relevant compounds, could also be prepared with stereochemistry being introduced through the chromium-catalyzed aldehyde functionalization, as disclosed by Winssinger and co-workers.^329^ Ligand **433** was again utilized toward the preparation of **459**, precursor to the targeted paraconic acid **460**, from the corresponding bromolactone and aldehyde with near perfect selectivity for **459** (ii, Scheme 68).

## 5 Applications of LNL-Carbazolide Pincer Complexes

### 5.1 Facilitation of Redox Activity and Redox Noninnocence

**Scheme 69:**
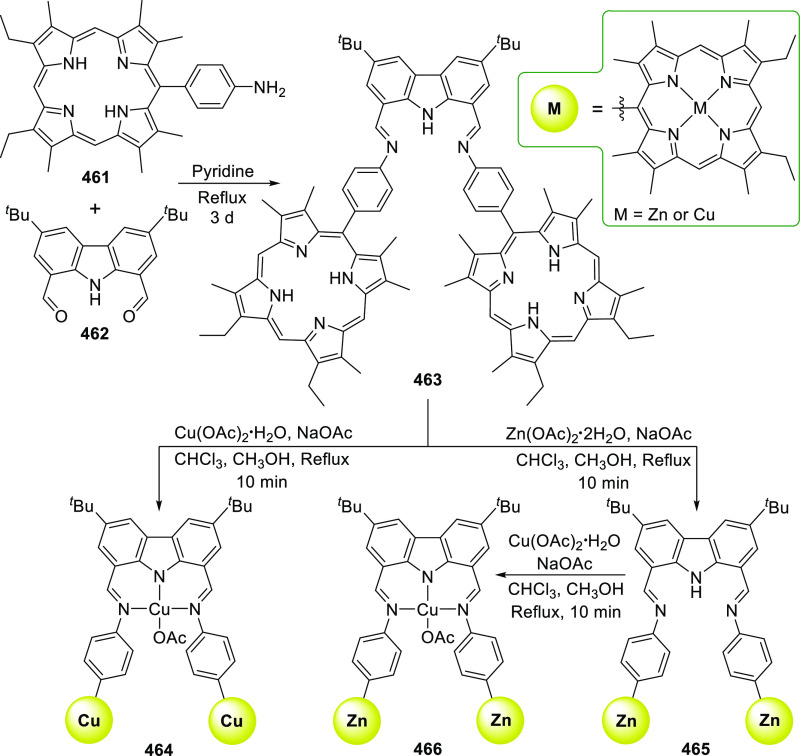
Synthesis of Pacman Diporphyrins Linked by a Rigid Copper(II) NNN-Carbazolide Bridge

For **LNL**-pincer metal complexes (**L** = N, P, C) containing an electrochemically active carbazole spacer,^330,331^ the ligand-based redox chemistry is observed in a different potential range (oxidations with *E*_1/2_ +0.4 to +0.7 V^263,332−335^ compared to other related **LNL**- type pincers, with *E*_1/2_ = −0.5 to +0.3 V)^336−339^ and includes the advantages of being reversible and readily tunable by carbazole-substituent modification.^335,340^ In the case of bi- and trinuclear dyads aimed at light harvesting, photovoltaic, and molecular electronic applications, this means that the redox activity can be tuned in the potential range outside that of commonly employed metalloporphyrins used in these applications.^341^ So-called “Pacman” porphyrins^342−345^ were prepared by Kadish et al., so that two porphyrins are linked in a cofacial arrangement by a rigid carbazole bridge to control the distance and the degree of opening of the two porphyrin macrocycles via changes in the extent of π–π interaction.^333^ The precursor bis(porphyrin)carbazole **463** was prepared by Schiff base condensation of the amino porphyrin **461** and the *tert*-butyl diformylcarbazole **462** (Scheme 69). Metalation with copper(II) acetate yielded the homonuclear trimetallic dyad **464**, or sequential metalation of the porphyrins with Zn(OAc)_2_ (**465**) followed by metalation with Cu(OAc)_2_ yielded the heteronuclear trimetallic copper, dizinc dyad **466** (Scheme 69). Without the presence of the third metal (Cu^II^) in the dyads (as represented by bimetallic dyad **465**), the two redox centers are noninteracting, as confirmed by cyclic voltammetry and ESR data. Consecutive oxidations of the two porphyrin zinc units involve one-electron abstractions at each metal center at the same potential to give a dyad with two linked porphyrin π-cation radicals, which is converted to another dyad with two linked porphyrin dications after the second one-electron oxidation at both metal centra, again at the same potential. If a Cu^II^ ion is coordinated to the central carbazole in the pincer pocket, however, an enhanced π–π interaction between the porphyrins of the tris-metal dyad results in a splitting of both the first two one-electron oxidations, as well as the second two one-electron oxidations, into two well-defined processes, respectively. Trimetallic dyad **464** similarly shows an interaction upon oxidation, which is only possible if a significant structural rearrangement occurs after the abstraction of one electron. A further consequence of the third metal (copper) presence in the central pincer pocket is the reduction of binding ability of the zinc porphyrins with axial ligands, as measured from the binding constants of **466** with chloride and acetate, compared to **465**.

**Scheme 70:**
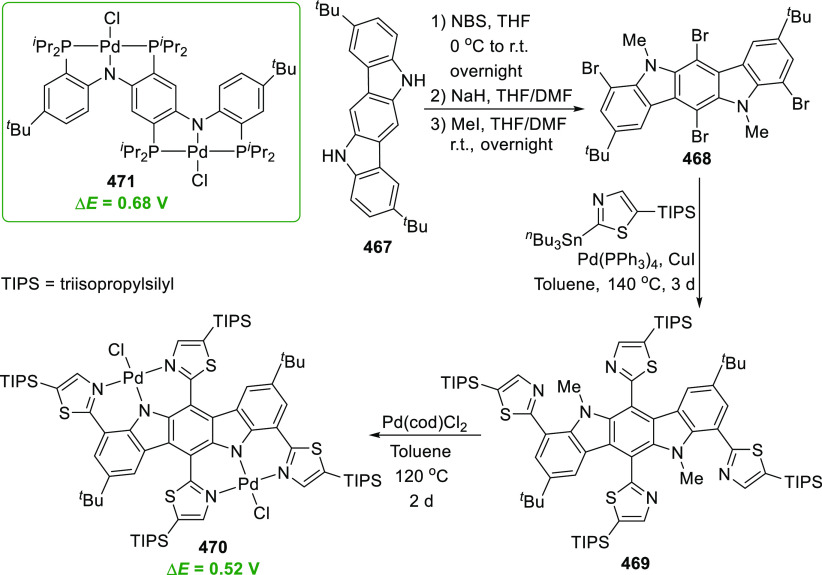
Synthesis of Palladium(II) Janus Pincers Containing Fused Indolo[3,2-*b*]carbazole Scaffold and Its Diphenylamide Bisphosphino Analogue

The persistence of organic aminyl radical cations is essential for their performance in organic electronic devices.^346^ The stabilization of the radical cations obtained from the oxidation of the arylamine derivatives can be achieved by coordination with transition metals, while providing the simultaneous opportunity for tailoring the electronic structure. In the context of this review, a carbazole-based pincer ligand coordinated to a low-spin palladium(II) would provide a planar system to prevent disruption of potential conjugation and resulting radical stabilization, while the coordinational lability of a Pd–Cl ligand would provide for potential further metal functionalization.^347^ With the introduction of a second metal center connected via a more extended conjugated backbone in a Janus-type pincer system, the redox sites would additionally be connected. The p-type organic semiconductor indolo[3,2-*b*]carbazole was used as the central core, provisioning for two NNN-pincers on either end. Following the bromination and protection of the N atoms of the precursor **467**, the fused carbazole precursor **468** can be functionalized with thiazole (Scheme 70). The resultant ligand precursor **469** features the pincer cores facing in opposite directions of the fused carbazole scaffold. Metalation of **469** with Pd(cod)Cl_2_ requires thermolysis of the N–Me bonds to obtain the bimetallic Janus pincer complex **470**. Two reversible oxidation processes are observed in the cyclic voltammetry study. The relation of the Δ*E* values with the comproportionation constant *K*_c_ classifies the complexes as belonging to the Robin-Day class III mixed valence compounds, with intermediate oxidation state exhibited by each redox site bridged by a scaffold very efficient in electron transfer.^348^ Surprisingly, the Δ*E* value (0.52 V) of **470** proved smaller than that of the bimetallic analogue (Δ*E* = 0.68 V) based on a diarylamido bisphosphino Janus pincer complex **471** with significantly smaller degree of coplanarity and conjugation (Scheme 70).^347^ This observation was rationalized as a consequence of significant electron delocalization in **470** compared to redox events that can be viewed as more “concentrated” in the central-diaminobenzene unit of the unfused analogue **471**. Visualization of the calculated SOMOs illustrates this conclusion, while the LUMO corresponds to a π* orbital of the fused ligand backbone in conjugation with the electron-deficient central thiazoles, leading to the lower orbital energies of the π-system.

**Scheme 71:**
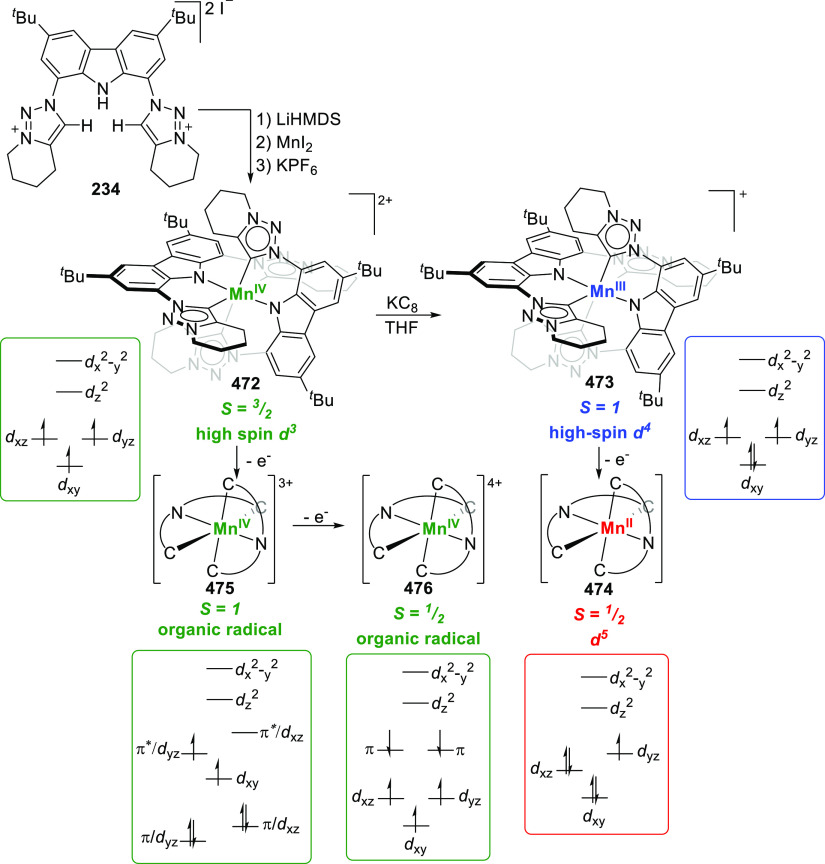
Synthesis of a Homoleptic Bis(triazolylidene)carbazolide Manganese Complex and the Electronic Structure of the Complex in Five Oxidation States

The redox noninnocence of the carbazole ligand, in conjunction with oxazoline donors, can be extrapolated to unusually facilitate both oxidation of monomeric ytterbium(II) pincer complexes, as well as metal reduction of a trivalent ytterbium complex analogue^349^ as a promising indicator. The combination of both central carbazole and donor group contribution to the redox versatility of the carbazole platform is particularly demonstrated in the report of a manganese complex coordinated to a bis(triazolylidene)carbazolide pincer ligand **234** (*vide supra*, Scheme 36).^350^ The ligand contains sterically undemanding *N*-fused triazolylidene wingtips that allow for variable oxidation state stabilization^351^ and homoleptic binding to a manganese precursor, in the synthesis of a rare example of an air-stable Mn^IV^ complex **472** (Scheme 71). Coupled with the redox-activity of the carbazole spacer group, the pincer scaffold provides for the observation of five different oxidation states, as observed by cyclic voltammetry and computational (DFT and CASSCF) calculations. Four reversible redox processes were observed, although only the isolation of the first reduced state could be accomplished by chemical reduction of **472** with KC_8_ to form the monocationic Mn^III^**473** (Scheme 71).^350^ SEC-EPR spectroscopy (SEC = spectroelectrochemistry) of the oxidized species **474** and **475**, as well as magnetometric measurements and magnetic circular dichroism measurements of the isolated **472** and **473**, gave experimental insight into their electronic structures, while the quantum chemical calculations elucidated the electronic structure of the entire series. Metal-based reduction of dication Mn^IV^**472** with *S* = 3/2 to an uncommon low-spin *d*^4^ configuration (*S* = 1) was confirmed with the unpaired electrons occupying the degenerate d_*xz*_ and d_*yz*_ orbitals for **473** (Scheme 71). A second metal-based reduction yields Mn^II^**474** with an overall spin of *S* = 1/2. The stepwise one-electron oxidations of **472** to **475** and **476** proceed via the consecutive removal of two electrons from the axial carbazolide π-donor ligands, leaving the manganese in a formal oxidation state of +IV. For **476**, a high-spin configuration of the Mn^IV^ leads to antiferromagnetic coupling of the two unpaired electrons to the two unpaired electrons located at the carbazolide-nitrogen atoms. The ligand-centered oxidations that give rise to (light-sensitive) Mn^IV^ organic radicals are particularly appealing for potential photoredox catalytic processes.

**Scheme 72:**
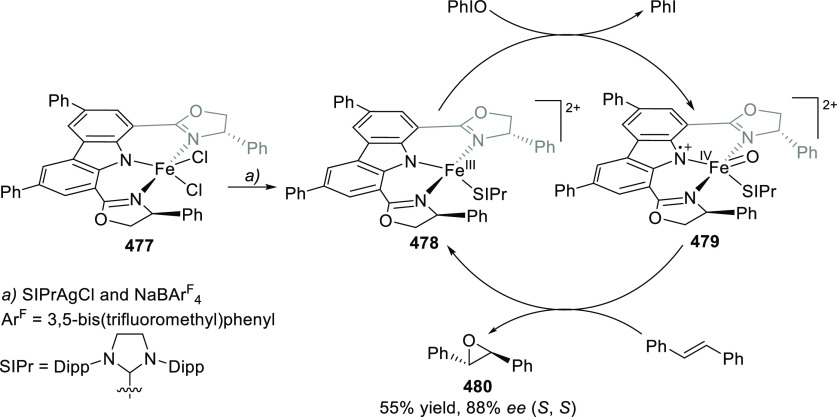
Asymmetric Epoxidation Catalyzed by a Porphyrin-Like Bis(oxazolinyl)carbazolide Iron Complex

In another example of the use of the bis(oxazolinyl)carbazole ligand in asymmetric catalysis by the group of Nakada (section 4.4), the preparation of a redox noninnocent NNN-carbazolide iron complex **477** (Scheme 72) was reported for oxidation reactions that parallel iron porphyrin complex reactivity.^352^ The similarity of **477** (Scheme 72) to the well-known iron porphyrin complex was drawn from the planar aromatic structure featuring an extended π-conjugation, in addition to the anionic amido moiety being a strong σ-donor. The asymmetric epoxidation of alkenes was investigated, taking advantage of the chiral wingtip sterics to control the selectivity of the resulting epoxides. It was found that **477**, in conjunction with catalytic amounts of NaBAr^F^_4_ (Ar^F^ = 3,5-bis(trifluoromethyl)phenyl), could catalyze the oxidation reaction yielding the epoxide with *ee* generally higher than 80% (Scheme 72). The requirement of the carbazole’s planar aromatic scaffold was evidenced when compared to the biphenylamide coordinated iron complex, which yielded only trace amounts of the epoxide. It was further established that addition of a catalytic amount of SIPrAgCl (SIPr = *N*,*N*′-bis(2,6-diisopropylphenyl)-4,5-dihydroimidazol-2-ylidene) improved the catalyst activity, leading to an increased yield of the targeted epoxide. In an effort to elucidate the reaction mechanism, a solution of **477** with NaBAr^F^_4_ and SIPrAgCl was analyzed, leading to formation of the *in situ* characterized dicationic iron complex **478** with an intermediate spin state of *S* = ^3^/_2_. Oxidation of **478** with iodosobenzene yields the proposed iron(IV) oxo complex **479** with a π-cationic radical and low spin state of *S* = 1/2, suggested to be a catalytic intermediate turning over alkenes to asymmetric epoxides. While the coordination number differs, the electronic structure of **479** is similar to that of iron porphyrin. The NNN-carbazolide therefore facilitates a two-electron oxidation which leads to a one electron event on both the ligand and the metal, while the chiral wingtip substituents dictate the selectivity of the catalyzed reaction.

### 5.2 Photophysical Properties and Photoredox Catalysis

**Scheme 73:**
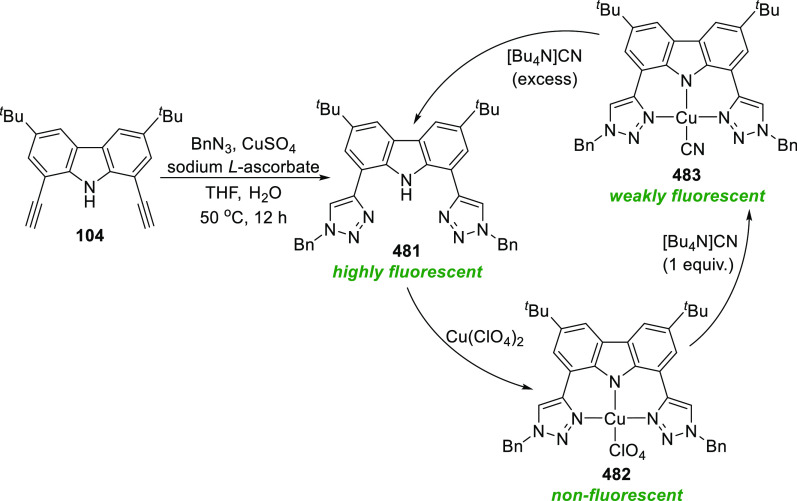
Proposed Cyanide Sensing Mechanism by Bis(triazolyl)carbazolide Copper(II)

The combination of the extended aromatic heterocycle of carbazole, and its easily accessible functionalization at positions 1, 8, 3, 6, and 9 (Figure 1), has ensured its popularity for photophysical and -chemical applications. Both its light harvesting properties (absorber in pigments, biodetectors, and bioimaging)^1,10,353−356^ and its light emission properties (OLED applications,^25,357−359^ fluorescence, and phosphorescence in photonic applications)^41,360,361^ are well explored. More recent, but already widespread, is the use of carbazole derivatives as organic photoinitiators to activate radical precursors in photocatalysis.^32,45−47,361,362^ Introduction of transition metals to carbazole-based photosystems allow for the development of organic electronic materials with a range of properties perhaps not attainable with organic molecules alone.^347^ Certainly, binding of a *d*-metal in the pincer pocket of a functionalized carbazole can significantly influence the photophysical behavior to either enhance photoluminescence as a result of decreased nonradiative decay in a less strained coordination environment of six-membered chelate rings,^363,364^ modulate emission color or mechanism, or the inverse effect: quenching emission.^365^ The fluorescence enhancement by ligand exchange and metal ion removal lends itself for the design of highly sensitive and selective ion sensing, as demonstrated for cyanide detection by a copper(II) bis(triazolyl)carbazolide pincer complex **482** (Scheme 73).^366^ The ligand precursor **481** was prepared from the copper-catalyzed alkyne-azide click-reaction (CuAAC)^367−369^ of the bis(alkynyl)carbazole precursor **104** and acts as fluorophore with fluorescence emission at *ca*. 385 nm.^366^ Coordination to a Cu^2+^ ion yields the stable, nonfluorescent **482**. If **482** is treated incrementally with tetrabutylammonium cyanide (TBACN), a two-step change in the UV–vis spectrum is observed, dependent on the stoichiometry of the reaction. After addition of 1 equiv of TBACN, ligand exchange at the copper is complete to form the weakly fluorescent copper(II) cyano complex **483** (Scheme 73). When excess cyanide is added, a marked increase in the fluorescence emission intensity is indicative of demetalation of **483** to form the stable Cu(CN)_2_ or Cu(CN)_4_ complexes with recovery of fluorophore **481**.

**Figure 7:**
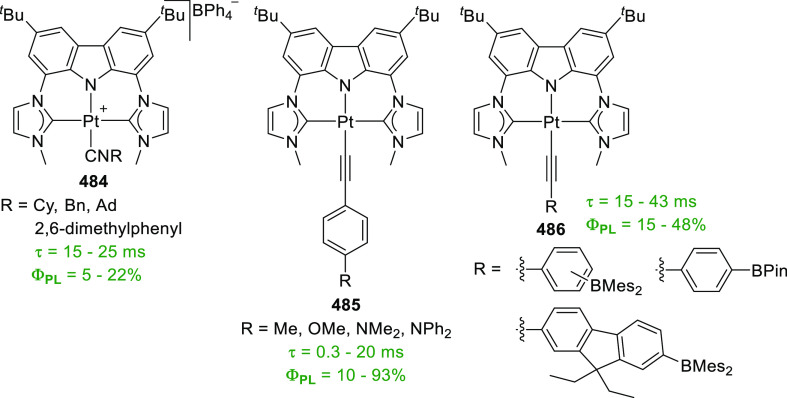
BIMCA-Pt(II) complexes investigated for their photophysical properties.

To achieve the opposite effect, namely preventing the quenching of photoluminescence, the BIMCA pincer ligand **92**(140) was chosen for complexation to platinum(II) with a series of ancillary isonitrile (**484**),^370^ alkynyl (**485**),^371^ and borane-substituted alkynyls (**486**)^372^ (Figure 7). The choice of BIMCA as CNC-pincer ligand was rationalized by its known stability toward hemilability and fluxionality to suppress potential nonradiative decay, while the steric influence of the flanking imidazolylidene wingtips were anticipated to prevent aggregation of the square planar Pt^II^ complexes in the ground state or in excited states as the cause of low emission yields and short lifetimes.^370^ In the first series of Pt^II^ emitters (**484**), π-accepting isonitriles were employed as ancillary ligands for charge transfer luminescence. The molecular structures obtained from single crystal X-ray diffraction confirmed the distorted square planar geometries of the complexes, with the isonitrile moieties protruding from the Pt-CNC plane. Moderate solid state emission was observed (emission maxima 460–475 nm), assigned as a LMCT, with absolute quantum yields up to 22% and millisecond decay lifetimes. Expanding their range of *d*^8^ triplet emitters, phenylacetylide BIMCA-Pt^II^ complexes **485** (Figure 7) were prepared to comprise of ancillary carbon-donor ligands to destabilize σ-antibonding ligand-field states, for increased intersystem crossing (ISC) from singlet excited states to longer-lived emissive triplet state deriving from the spin–orbit coupling of the heavy metal.^371^ The same rationale as for **484** was followed for the use of the BIMCA pincer ligands in **485**; the nonplanar chelation geometry and bulky substituents of the CNC-pincer scaffold, as well as the ligand-field strength of the ligand, allowed for the marked increase in emission quantum yield and lifetimes as solids (up to 93% with an 11 ms lifetime at 298 K). No evidence for π-stacking or metallophilic interactions was observed in the solid state structures, with the alkynyl ligand displaced from the BIMCA ligand plane. From the time-dependent DFT calculations performed, the phosphorescence was found to arise from a state with mixed ^3^(MLCT/LL) character, with the BIMCA ligand dominating the frontier orbitals. Deactivation by ligand-field excited states is inhibited by the combined ligand fields of the BIMCA and alkynyl ligand to banish the σ-antibonding d_*x*^2^–*y*^2^_ orbital 1.5 eV above the HOMO.

To promote intramolecular charge transfer luminescence between the *d*^8^ emitter and the alkynyl ancillary ligand, different borane-substituted alkynyls were employed as ancillary ligands in the series **486** (Figure 7).^372^ In complex series **484**(370) and **485**,^371^ emission is localized on the BIMCA core. Incorporation of the borane substituents were proposed to combine the strongly absorbing BIMCA core with efficient spin–orbit coupling from Pt^II^ and the Lewis acidity of three-coordinate boron as electron-accepting units.^373−375^ Visualization of the calculated Kohn-Sham orbitals of **486** suggests charge transfer between the BIMCA-Pt localized HOMO and the alkynylboron ligand centered LUMO was achieved, to provide for charge separation that enables ligand/metal-to-ligand charge transfer (LMLCT).^372^ The resulting emission of **486** is longer lived (up to 43 ms) than observed for **484** or **485**, but the emission is comparatively weaker. Possible sources of thermal vibrational energy dissipation were proposed to be either the methyl wingtips of the BIMCA ligand or the alkynyl ligands.

Three-coordinate complex geometries can be tailored by variation of the metal-ligand dihedral angles to demonstrate tunable behavior from pure phosphorescence to thermally activated delayed fluorescence (TADF) for the *d*^10^ coinage metals,^376^ although few ligand scaffolds are available for photophysical tuning of the triplet-singlet gap with ground state Jahn-Teller distorted T-shapes.^377^ Beyond the steric directing effect employed in the above-mentioned examples to ensure nonplanarity, the employment of CNC-pincer ligands based on a carbazole scaffold can also be used to dictate the coordination geometry of photoluminescent *d*^10^ coinage metals, while the electronic effect of the carbazole amido allows for postcomplexation modification of the metal center to modulate the photophysical behavior.^197^ The combination of a carbazolide with two flanking 1,2,3-triazol-5-ylidenes yields such T-shape geometries for all three of the coinage metals Cu^I^ (**110**),^193^ Ag^I^ (**111**),^197^ and Au^I^ (**113**)^186^ (*vide supra*, Scheme 17), whereby three of the design principles outlined in this review (electronic effect of metal-amide bond, mesoionic nature of flanking carbene **L**-donors, and steric directing bulky wingtip **R**-groups) are applied. The unique reactivity of **113** with electrophiles provides the opportunity to modify the gold(I) complex by electrophilic attack either at the amide to yield the cationic linear Au^I^ complex **117**(186) or at the nucleophilic metal to furnish the Au^III^–F square planar complex **121** (Scheme 18).^197^ Emissions extending from the blue (copper) to green (gold) to orange (silver) spectrum originate from metal-perturbed π(carbazolide)−π*(carbene) ^3^ILCT excited states. However, low quantum yields were observed, with the highest quantum yield (Φ_PL_ = 14%) for the linear Au^I^ complex **117**. Reverse intersystem crossing (rISC) is prohibited by a larger triplet-singlet gap to suppress TADF, with greater phosphorescence contributions to the photoluminescence leading to longer decay times in THF at room temperature. On the other hand, the lifetime of Cu^I^ complex **110** is too short (subnanosecond) to be determined with certainty, and emission may be fluorescent in nature. For the Ag^I^**111** and Au^I^**113** analogues, decay lifetimes in the microsecond range increase to millisecond lifetimes upon cooling to 77 K. This increase is in line with the assignment of a change in emission origin from ^3^ILCT to ^3^IL excited state and may suggest TADF.

**Figure 8:**
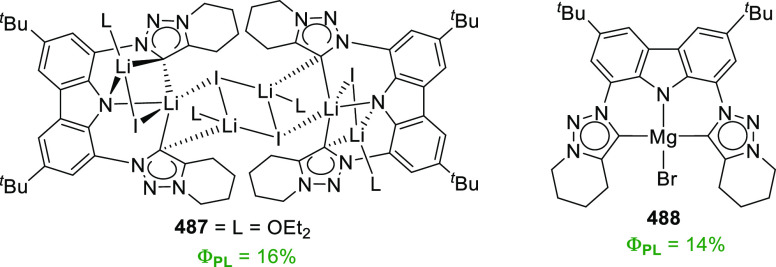
Photoactive bis(triazolylidene)carbazolide complexes of the *s*-block metals.

If the modified bis(triazolylidene)carbazolide pincer ligand precursor **234** (see section 3.2 and 5.1) is employed, where the carbene donor heterocycles are connected to the carbazole via the N1-triazole atom instead of the C4-triazole atom, isolation of a photoactive lithium dimeric structure (**487**), bridged by iodo- and lithium iodide adducts, can be achieved.^241^**487** is the precursor for the mononuclear magnesium bromide complex **488** (Figure 8). The use of these (earth) alkali metals prevents ligand distortion following excitation to avoid nonradiative deactivation, and blue-green and intense lime-green luminescence in solution were observed for **487** and **488** (Φ_PL_ = 16% and 14%, respectively) at room temperature, rationalized by ILCT from the carbazolide to the mesoionic carbenes as derived from the quantum chemical calculations.

**Figure 9:**
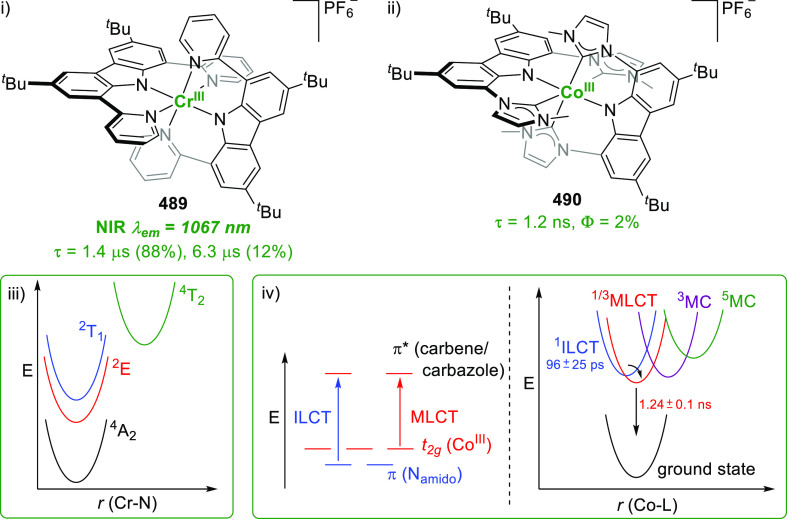
Homoleptic complexes of the high-valent 3*d* metals (i) chromium(III) and (ii) cobalt(III). Configurational coordinate diagram (iii) for *O*_*h*_ symmetry energy states of **489**(378) and (iv) key electronic transitions occurring in **490**, with configurational coordinate diagram of energetically low-lying charge transfer and MC excited states with time constants of two relaxation processes.^379^

Two highly successful examples of ligand design for tuning of the photophysical properties of 3*d* metals were recently reported by Wenger and co-workers.^378,379^ The advantages of carbazole-based pincer ligands were exploited, including the π-donating character of the carbazolide-nitrogen, the use of either π-accepting pyridines^378^ or strong σ-donating carbenes^379^ as flanking donors, with rigid meridional geometry allowing for bite-angle optimized 6-membered chelated metallacycles to avoid nonradiative relaxation, and low steric bulk of the wingtips on the donor moieties to facilitate homoleptic complex formation. For the first-row metal ion chromium(III), design strategies for emissive complexes have focused on increasing the energy gap between the emissive ^2^E and the ^4^T_2_ excited state by enhancing the ligand field strength,^378^ to minimize nonradiative relaxation from the higher-lying state and, consequently, optimizing luminescence quantum yields (iii, Figure 9). Lowering of the ^2^E state energy, however, directly affects the ^2^E → ^4^A_2_ spin-flip transition of Cr^III^ to tune the emission color and is more susceptible to electron-electron repulsion than ligand field strength. For metal complexes with more covalent metal-ligand bonds, *d*-electrons are spatially more distributed and less confined to the metal core, in the so-called nephelauxetic effect.^380,381^ The mutual repulsion between the *d*-electrons is quantified by the Racah *B* parameter, with a decrease in *B*-values indicating that the repulsion diminishes. By complexation of a bis(pyridine)carbazolide pincer ligand to yield the (NNN)_2_Cr^III^ complex **489** (i, Figure 9), Wenger et al. accomplished a shift in the ^2^E emission to 1067 nm at 77 K (compared to the range of 727–778 nm reported for typical Cr^III^ polypyridine complexes in the red to NIR-I spectral region), classifying **489** as the first example of a Cr^III^ NIR-II emitter.^378^ This large red-shift to the NIR-II region was attributed to the unusually strong metal–ligand bond covalence facilitated by π-electron density donated from the ligands to the metal in the axial direction, whereas in the equatorial plane π-electron density flows from the metal toward the pyridine π-acceptors. These push-pull interactions induce the strong nephelauxetic effect observed.

Similarly for the 3*d*^6^ metal Co^III^, the choice of a CNC-pincer ligand based on the carbazole spacer group allows for the isolation of a low-spin configuration identical to that of Ir^III^ emitters.^379^ The BIMCA protioligand **92**(140) was utilized as a precursor. The basic imidazolylidenes as strong σ-donor flanking groups, promote stabilization of the high-valent central metal against ligand dissociation upon photoexcitation of homoleptic **490** (ii, Figure 9), while the increased covalency of the carbazolide-metal bond was once again exploited to effect a decrease in the Racah *B* parameter determining the metal-centered (MC) states.^379^ In this way, **490** was demonstrated to feature a photoactive excited state with substantial MLCT character, in contrast to the typically observed low-lying LMCT states.^382,383^ The photoactive excited state of **490** has an intraligand contribution, resembling the photoactive excited states with mixed MLCT/ILCT character of cyclometalated iridium(III) emitters (iv, Figure 9).^379^ The excited state decays to the electronic ground state without a noticeable population of any MC state, rationalizing the lack of photoluminescence in **490**. UV-vis transient absorption spectroscopy and spectro-electrochemical investigations revealed the unexpected photostability of **490** and its participation in photoinduced electron transfer reactions. Both the photophysical and -chemical behavior of **490** were emphasized to depend on a ligand design rationale comprising of ligand field strength optimization (σ-donor and π-acceptor properties) and increased metal-ligand bond covalence, accessible by using π-donor ligands for enabling a lowest excited state with MLCT character that is not efficiently depopulated by MC (metal centered) states shifted to high energies (iv, Figure 9).

**Scheme 74:**
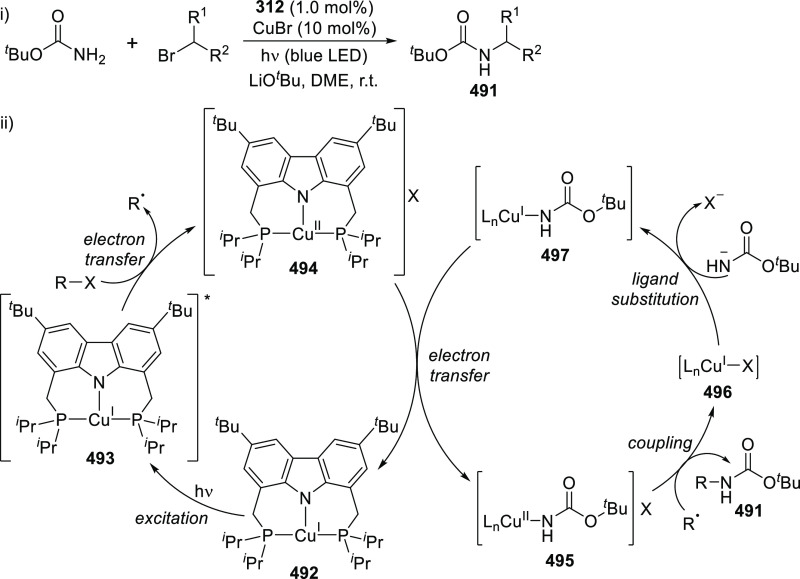
(i) Direct Synthesis of Carbamate-Protected Primary Amines with (ii) Supplementary Proposed Catalytic Mechanism

Facile preparation of carbamate-protected primary amines from the corresponding carbamate and the unactivated secondary alkyl halides was made possible through the use of a copper-coordinated PNP-carbazolide complex under blue LED irradiation.^384^ The group of Peters and Fu, focusing on photoinduced copper-mediated coupling processes,^385−393^ prepared the photocatalyst **492** for the coupling reaction in the presence of CuBr and LiO^*t*^Bu, at room temperature under light irradiation (i, Scheme 74).^384^ On the basis of the results obtained, a mechanistic pathway was proposed whereby irradiation of the copper carbazolide **492** by blue LED lamps generated the excited state intermediate **493** (ii, Scheme 74). Reduction of the electrophile follows, leading to the alkyl radical and a copper(II) intermediate **494**. This SET process was supported by EPR spectroscopy, which confirmed the *in situ* formation of the paramagnetic **494**. The Cu^II^**494** oxidizes a copper(I)-nucleophile complex **497**, leading to the copper(I) carbazolide **492** and the oxidized **495**. An out-of-cage coupling reaction between the alkyl radical and the Cu^II^**495** yields the cross-coupled product and **496**, in turn, reacting with a nucleophile leading to **497** via a ligand substitution reaction.

### 5.3 Beyond 3-Coordinate Pincers: Expansion of the LNL-Carbazolide Pincer Motif to Polydentate Systems

Expansion of the tridentate binding motif of the carbazole-based pincer ligands under review is a topic with surprisingly few examples. In section 4.3.3, the introduction of one or two homoallyl groups tethered to the carbene-donor imidazolylidene-nitrogen of the BIMCA ligand resulted in tetra- or pentacoordinate ligand coordination, whereby the introduction of the olefinic donors not only yielded more nucleophilic metal centers (**383**, Scheme 59 and **392**, Scheme 60) with enhanced catalytic performance but also demonstrated cooperative or chemically noninnocent metal-ligand reactivity in the proposed catalytic pathways.^299−301^ In a related example, the BIMCA ligand was modified to contain a pentamethylene tether connecting the two imidazolylidene donor ligands (**199**), to facilitate dehydrogenation (**379**) and C–H activation (**380**) reactivity in their binding to the central metal atom (see Scheme 57).^134^

**Scheme 75:**
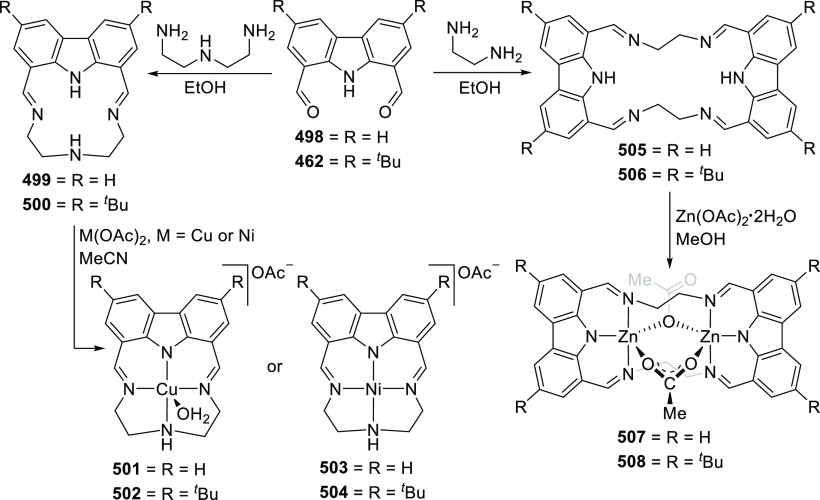
Synthesis of Mono- And Dinuclear Macrocyclic Bis(imine)carbazolide Metal Complexes

Utilization of the diformylcarbazole precursor **462** and **498** paves the way for introduction of ancillary imine groups by way of Schiff base condensation, for the synthesis of macrocyclic ligands with up to six ligating nitrogen atoms.^394,395^ Changing the ligand scaffold to comprise of a carbazole head unit instead of the previously studied pyrrole of diphenylamine based head units in the Schiff base macrocycles leads to (i) modification of the p*K*_a_ (diphenylamine p*K*_a_ = 25, pyrrole p*K*_a_ = 23, and carbazole p*K*_a_ = 20 in solvent dimethylsulfoxide),^396,397^ (ii) structural changes including decreased flexibility and bite angle changes, and (iii) variation of the delocalized electron density on the head unit. The group of Brooker et al. prepared the [1 + 1] macrocycles **499** and **500** directly by refluxing of the respective dialdehyde precursors **498** or **462** and diethylenetriamine in ethanol, followed by addition of the metal(II) acetate salt in acetonitrile solution (Scheme 75).^394^ In the case of Cu(OAc)_2_, five coordinate square pyramidal complexes **501** and **502** with an ancillary aqua ligand in the axial position were isolated, while the diamagnetic nickel(II) complexes **503** and **504** featured square planar geometry as a result of the strong ligand field imposed by the macrocycle.

In a similar metal-free condensation procedure, the [2 + 2] Schiff base macrocycles **505** and **506**, containing two carbazole units tethered by two diamine units (Scheme 75), were prepared using ethylene diamine, followed by metalation with zinc(II) acetate.^395^ The resulting dizinc complexes **507** and **508** retain the stepped conformation of the metal-free macrocycles **505** and **506**, despite deprotonation and binding of two Zn^II^ centers in the two tridentate pockets, with one acetate anion displaying μ_1,1_-bridging and the other μ_1,3_-bridging. **507** and **508** are strongly blue fluorescent (λ_max_ = 460 nm). Subsequent fluorescence studies demonstrated the strong selectivity of **505** and **506** for Zn^II^ ions over metal(II) ions (Ca, Mg, Mn–Cu) in a turn-on fluorescence to highlight potential applications for these Schiff base carbazole-based macrocycles.

**Scheme 76:**
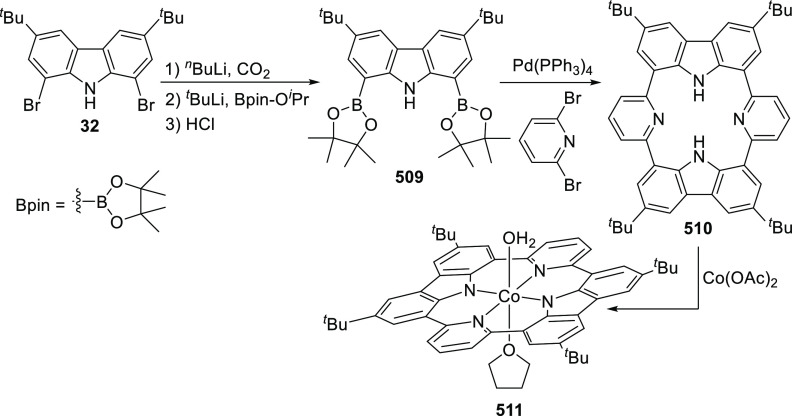
Synthesis and Metalation of Porphyrin-Like Macrocycles Based on Carbazole and Pyridine Units

The resemblance of carbazole-based macrocycles to porphyrinoids portends well for their diverse application. A calix[4]pyrrole[2]carbazole macrocycle was prepared nearly two decades ago and employed in anion sensing applications via fluorescence quenching means; however, no metalation of the macrocycle was reported.^398^ The first example of a porphyrin-related macrocycle coordinated to the transition metal cobalt was only reported in 2011, with the aromatic carbazole and pyridine blocks connected exclusively via aryl-aryl bonds.^399^ Four-fold cross-coupling of the diboronic ester carbazole precursor **509** with 2,6-dibromopyridine was achieved by Suzuki-Miyaura coupling to yield the macrocycle **510** (Scheme 76). The macrocycle displays a saddle-like conformation with the pyridine moieties twisted “up” and the carbazoles twisted “down”. The ligand design permits the formation of a cavity big enough to complex cobalt(II) to form a distorted octahedral complex **511**, with solvent molecule thf and a water molecule axially coordinated. The saddle conformation is maintained upon metal binding.

**Scheme 77:**
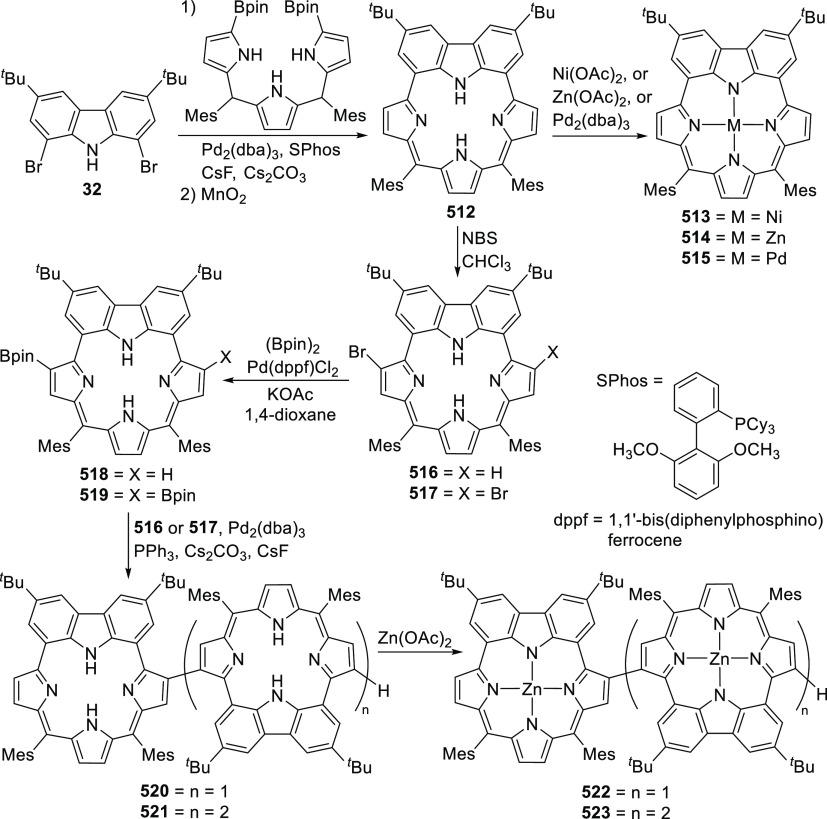
Synthesis and Metalation of Porphyrin-Like Macrocycles Based on Carbazole and Pyrrole Units

The interest in carbazole incorporation into fused porphyrins lies in the consequent electronic delocalization over the carbazole and pyrroles to promote conjugation and intramolecular electronic communication. By employing Suzuki-Miyaura cross-coupling reaction conditions with dibromocarbazole **32** and diboryltripyrrane, macrocycle **512** could be synthesized (Scheme 77).^400^ Selective bromination with NBS yields **516** and **517**, for subsequent boryl functionalization to **518** and **519**. A follow-up coupling reaction of **516** and **517** with **518** and **519** yields the dimeric **520** and trimeric **521**. Metalation of **512** with the divalent metal salts (Pd^2+^, Zn^2+^, or Ni^2+^) yields the complexes **513**–**515**. Similarly, reaction of zinc(II) acetate with **520** or **521** leads to bi- and trimetallic complexes **522** and **523**, respectively (Scheme 77). NMR spectroscopic studies yielded no evidence for a global and macrocyclic aromaticity in the macrocycles **512**, **520**, or **521**. The HOMO and LUMO showed relative delocalization over the pyrrole and carbazole units for the metal compounds compared to the metal-free compounds, with insertion of the metal ion into the cavity proposed to strengthen the conjugation. The photophysical behavior of the Zn^II^ porphyrins **514**, **522**, and **523** was studied and the exciton coupling strength between the carbazole-tripyrrole units determined to be stronger than that of linear Zn-porphyrin arrays. The greater degree of torsional freedom between the constituent units of the dimer **522** and **523** allowed for torsional relaxation in the excited state dynamics, with the mixing of the higher-lying charge transfer state with an exciton coupled state leading to a reduction of the fluorescence properties of the carbazole-tripyrrolic arrays.

**Scheme 78:**
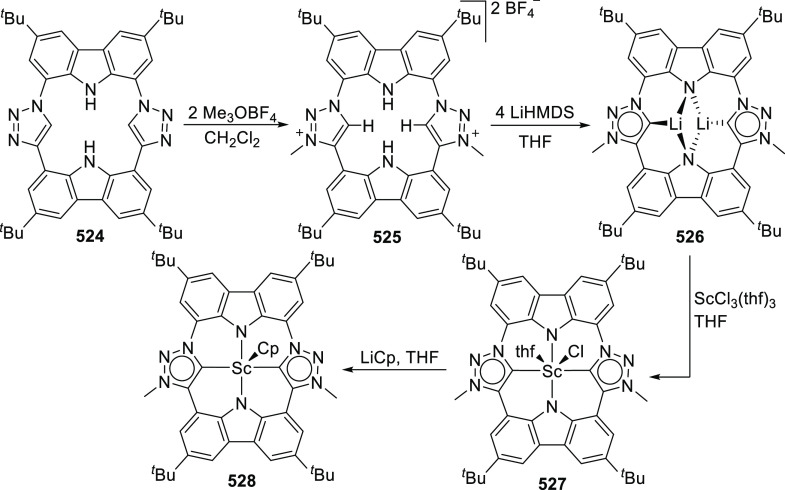
Synthesis, Deprotonation, And Metalation of Carbazole-Triazolylidene Porphyrin

A recent addition to the class of carbazole-based porphyrinoid ligands is the carbenaporphyrin reported by Kunz.^401^ The synthesis of the carbenaphorphyrin is dependent on the substitution of the pyrrole units not with imidazole-based NHCs but with triazole-based carbenes accessible by the facile click reaction (CuAAC)^367−369^ of the bis(alkynyl)- and bis(azido)carbazole precursors. Optimization of the 1,3-dipolar cycloaddition to obtain the carbazole macrocycle **524**, followed by alkylation of the triazole rings with Meerwein’s salt, yielded the dicationic macrocycle **525** (Scheme 78).^401^**525** is colorless, indicating that it lacks antiaromatic (20 e^–^) or a macrocyclic aromatic π-system like porphyrin (18 e^–^). This was supported by visualization of the calculated frontier orbitals, where the individual aromatic character of the contributing ring-units is retained and the HOMO almost fully localized on one carbazole unit. Deprotonation with a lithium base forms the corresponding dilithio-carbenaporphyrin complex **526**, with a N,C,N-η^3^-coordination of each lithium atom supported by DFT calculations, in addition to the coordination of two solvent thf molecules. Transmetalation with scandium trichloride yields the scandium porphyrin complex **527**, with the η^4^-coordination of the carbenaporphyrin ligand in the basal plane confirmed by X-ray structure analysis while the additional chloride and solvent thf molecule are coordinated cis above the plane. To probe for potential ring current in **527**, it was reacted with CpLi (Scheme 78). The ^1^H NMR spectrum of **528** displayed Cp–H signals in the expected region, and the lack of shielding effect observed precludes any macrocyclic aromatic or antiaromatic ring effect. Nevertheless, the ligand scaffold provides the geometric features common to porphyrins upon complexation with the metals. In addition, the mesoionic nature of the triazolylidenes provides for stronger electron-donor properties of the carbenaporphyrin ligand compared to porphyrins, as evaluated by the calculated Mulliken population analyses of the scandium metal center.

## 6 Outlook

### 6.1 Combinatorial Effect

The combination of more than one of the building-up strategies, incorporating multiple control points (with carbazole-**N** as prerequisite, in combination of **E**, **L**, **R**, and **Z**, Figure 1) listed progressively in the preceding sections 2–5, generates exciting possibilities for multifunctional and adaptive molecules in different disciplines. As a summative perspective to the scope of this combinatorial effect for guiding future synthetic methodologies, we wanted to highlight “case studies” as illustrative examples of the potential of the **LNL**-carbazolide pincer, taking advantage of the inherent attributes of carbazole which has accounted for its success in medicinal, redox-, and photoactive applications.

In the area of anticancer therapeutics based on metallodrugs that can overcome the challenges presented by platinum-based compounds, gold-based anticancer agents can induce cytotoxicity either by targeting DNA (or other intracellular proteins) via direct covalent binding or noncovalently via intercalation if stabilized as Au^III^ in an appropriate multidentate ligand scaffold.^402^ The tendency of the gold(III) complexes to be reduced to gold(I) or metallic gold under physiological conditions, as well as the nonselectivity of DNA-binding, limit their usefulness as chemotherapeutics. Appropriate choice of a CNC-pincer ligand that provisions a planar aromatic backbone for cytotoxicity against cancer cells by the inhibition of DNA topoisomerases, with simultaneous stability against intracellular reductants, is found by modifying the bis(triazolylidene)carbazolide scaffold (see section 2.3). The 3,6-positions of carbazole is thus unsubstituted for DNA intercalation, while the combination of the donor (**L**) mesoionic carbenes and remote basicity effected by the carbazolide-gold(I) interaction^186^ is required for both the preparation and proven stabilization of the CNC-Au^III^ complexes as potential anticancer agents with multicellular targets.^403^ In this case study, the triazolylidene wingtip groups also played a significant role in the isolation of well-defined mononuclear pincer complexes.

**Scheme 79:**
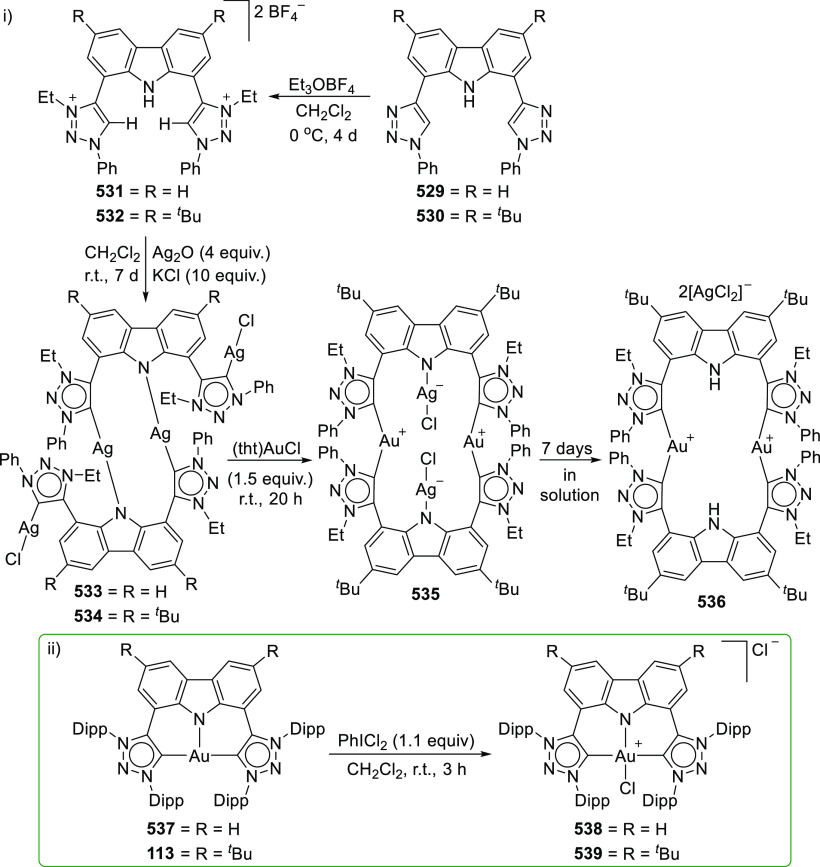
Synthesis of (i) Alkylated Triazolium Salts and Their Polynuclear Silver(I) and Gold(I) Complexes, as well as (ii) Bis(triazolylidene)carbazolide Coordinated Cationic Au^III^ Chloride Complexes

The precursor triazolium salts **531** and **532** were prepared from 1,4-disubstituted 1,2,3-triazoles **529** and **530**, followed by alkylation of both triazoles on their N3-positions (i, Scheme 79).^403^ The kinetic instability of the N3-alkylated triazolylidenes during direct metalation with *in situ* generated free carbenes compelled the use of transmetalation techniques for gold(I) complex formation from the alkylated protioligands **531** and **532**. The ligands were first metalated with Ag_2_O in the presence of excess KCl, yielding tetranuclear monocarbene silver(I) complexes **533** and **534** as intermediates (i, Scheme 79). However, when the *tert*-butyl analogue was further reacted with the gold(I) metal precursor, the reaction unexpectedly afforded the tetranuclear zwitterionic Au^I^ bis(carbene) complex **535**, which was not stable in solution and decomposes to the cationic dinuclear Au^I^ bis(carbene) complex **536** within a week.

To circumvent multinuclear complex formation, 1,2,3-triazolium salts with two bulky aryl (Dipp) substituents on the triazole rings, **106**(193) and the corresponding unsubstituted analogue featuring hydrogens instead of ^*t*^Bu groups on the carbazole scaffold^403^ were prepared using the previously reported methodology^193^ to facilitate mononuclear complexation with the desired pincer-coordination via deprotonation and *in situ* carbene formation.^403^ The reaction of the *in situ* deprotonated ligand with gold(I) precursor yielded the gold(I) complex **537**. As for **113**,^186^ the complex **537** also has a three-coordinated gold center with a T-shaped geometry.^403^ The demonstrated reactivity of the T-shaped gold(I) complexes with electrophiles^186^ was employed in the oxidation with iodobenzene dichloride to their respective cationic gold(III) complexes **538** and **539**.^403^ The resulting monomeric, square planar complexes feature a labile chloride ligand available for covalent DNA-bonding, thus furnishing another avenue to target cancer DNA (ii, Scheme 79).

When investigating the cytotoxicity of the Au^III^ complexes **538** and **539**, as well as the Au^I^ complexes **537** and **113** against the breast cancer cell line MDS-MB-231, notable cytotoxicity of the Au^III^ complex **538** was observed (IC_50_ = 2.3 ± 0.8 μM), although the ligand precursor was even more cytotoxic (IC_50_ = 0.4 ± 0.1 μM).^403^ However, the selectivity of the ligand precursor was found to be lower compared to **538**. The underlying factors of the cytotoxicity shown by **538** were clarified in further studies. The interaction between **538** and DNA was investigated using several techniques, which suggested that the complex behaves as a partial DNA intercalator. In addition, *in silico* screening indicated that **538** can target DNA three-way junctions and several other forms of *β*- and *Z*-DNA with good specificity. The redox stability of **538** under physiological conditions was evaluated in the presence of the intracellular reductant glutathione. The complex was found to be stable and neither reduction of the **538** to **537** nor demetalation was observed in the presence of excess glutathione. The redox stability of **538** combined with its DNA affinity are attributed as the key factors for its cytotoxicity *in vitro*. The synthetic feasibility of **538**, in turn, is reliant on the combination of donor oxidation state tolerance, backbone, and wingtip sterics as well as the carbazolide electronics for the needed remote basicity of gold.

**Scheme 80:**
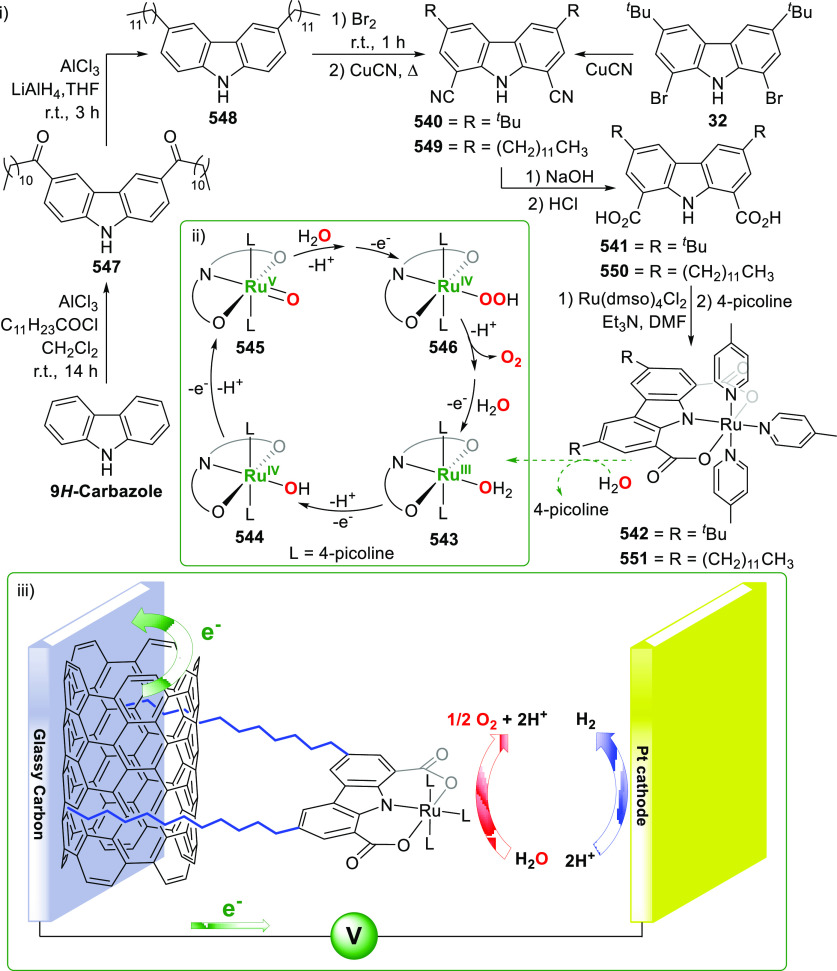
(i) Synthesis of Charge-Neutral ONO-Ru Complex As Water Oxidation Catalyst, (ii) Proposed Mechanism for the Catalytic Cycle of Water Oxidation Mediated by 542 or 551, and (iii) Composite Molecular Anode 551/MWCNTsCOOH/GC Constructed in the Electrochemical Cell for Efficient Water Splitting^409^

The advantage of the redox-stability attainable by the use of the **LNL**-carbazolide, in combination with 3,6-carbazole backbone modification and judicious choice of anionic O-donors as the flanking **L** groups, is similarly well-illustrated in the preparation of a composite molecular anode for water oxidation catalysis (WOC).^404^ Despite the evident utility of a trianionic ONO-pincer ligand based on a carbazole scaffold as demonstrated in section 2.4, very few examples of this type of pincer ligand are known, in contrast to the well-explored NNN, PNP, and CNC-analogues. In this review, only two examples of ONO-carbazolide pincers are noted, including precursor **122**(205) (vide supra) and **541**,^404^ the analogue of **45** with the carbazole backbone containing methyl groups in the 3,6-positions of the carbazole. The dicarboxylic acid-functionalized **541** was prepared stepwise from **32** by cyano-substitution of the bromines followed by hydrolyzation (i, Scheme 80). Synthesis of the ruthenium(III) complex **542** coordinated by the dicarboxylate-carbazole ONO^3–^ ligand was done by reaction of **541** with [Ru(dmso)_4_Cl_2_] (dmso = dimethylsulfoxide) under basic conditions, followed by addition of excess 4-picoline. The choice of ligand was rationalized with the target of preparing a water oxidation catalyst that can access high oxidation states and form Ru-oxo species across a narrow potential gap by a proton-coupled electron transfer (PCET) pathway,^405^ while maintaining a low catalytic overpotential to match the oxidation potential of photosensitizers for effective light-driven water oxidation. A trianionic pincer ligand based on a conjugated carbazole scaffold would permit a sufficiently low redox potential to allow for water oxidation to be driven by visible light because of its strongly electron-donating character derived from the three anionic donor moieties (central carbazole-**N** and flanking carboxylates), as well as the rigid planar structure to stabilize the ruthenium center in high redox states.^404^ From the single crystal X-ray analysis of the molecular structure of **542**, occupation of the two axial positions and one equatorial position *trans* to the carbazolide-nitrogen by the coordinating 4-picoline ligands was confirmed. The longest Ru–N bond length was observed for the picoline group trans to the carbazole-amido, indicating that ligand exchange of 4-picoline with water would most likely occur at that position. The improved donating ability of the ONO-carbazolide ligand was demonstrated by the three oxidation potentials observed for the Ru^II^/Ru^III^, Ru^III^/Ru^IV^, and Ru^IV^/Ru^V^ oxidation processes (namely, *E* = 0.20, 0.63, and 0.99 V, respectively, vs NHE), at oxidation potentials markedly lower than those reported not only for WOCs containing neutral ligands but also for Ru-based WOC with anionic ligands.^406−408^**542** proved an efficient WOC catalyst in the Ce^IV^-driven process with an initial TOF of 0.28 s^–1^.^404^ The active intermediate involved in the catalytic cycle was determined by high-resolution mass spectrometry as being the Ru^IV^-OH species **544** (shown in the proposed catalytic cycle ii, Scheme 80), where ligand exchange of 4-picoline with water already occurred in the Ru^III^-state (**542** + H_2_O-4-picoline → **543**). The PCET process of **542** was monitored by evaluating the dependence of the potentials on pH, indicating typical one-electron, one-proton PCET processes for the three oxidation steps, corresponding to the H-Ru^II^-picoline/Ru^III^-OH_2_ (**543**), Ru^III^-OH_2_/Ru^IV^-OH (**544**), and Ru^IV^/Ru^V^=O (**545**). These experimental results form the basis for the proposed catalytic cycle of water oxidation as catalyzed by **542** (ii, Scheme 80), with the critical O–O bond formation step occurring via the nucleophilic attack of water to form Ru^IV^-O-OH (**546**) which releases O_2_ via a reductive elimination step to regenerate **543**. The low onset potential (1.24 V vs NHE) measured under neutral conditions suggested that **542** could catalyze water oxidation driven by visible light with a photosensitizer. Very good activity with an initial TOF of 5 min^–1^ was observed in this three-component system.

The performance of the mononuclear Ru^III^ catalyst **542** was significantly improved by the same group, with the expedient of first modifying the carbazole-backbone to contain two dodecyl chains instead of *tert*-butyl groups on the 3,6-positions (**548**, i, Scheme 80).^409^ Functionalization of the 1,8-positions to contain two carboxylic groups proceeded in the same way as for ligand **541**,^404^ to yield the ONO-ligand precursor **550** for complexation to ruthenium in preparation of the charge-neutral mononuclear Ru^III^**551** (i, Scheme 80).^409^ The hydrophobic features of the dodecyl chains allow for easy binding to carbon nanotubes and immobilization of **551** on COOH-functionalized multiwalled carbon nanotubes (MWCNTsCOOH) followed by loading onto the surface of a glassy carbon electrode yielded the composite molecular anode **551**/MWCNTsCOOH/GC illustrated in iii, Scheme 80. Following the same mechanistic route as proposed for the previously reported **542** (ii, Scheme 80), the charge-neutral intermediates **543**–**546** were proposed to stabilize the catalytic center and promote the durability of the assembled molecular anode. With this assembly, excellent catalytic activity for the oxygen evolution reaction (OER) is reported, with an onset overpotential of only 380 mV (compared to the 1.24 V for **542**)^404^ and a steady catalytic current density of 1.25 mA/cm^2^ at the overpotential of 580 mV.^409^ A Faradaic efficiency of 96% and a TOF of 10.3 s^–1^ for OER could be achieved from this molecular device as a demonstration of its high efficiency and durability in electrochemical catalytic water oxidation.

**Scheme 81:**
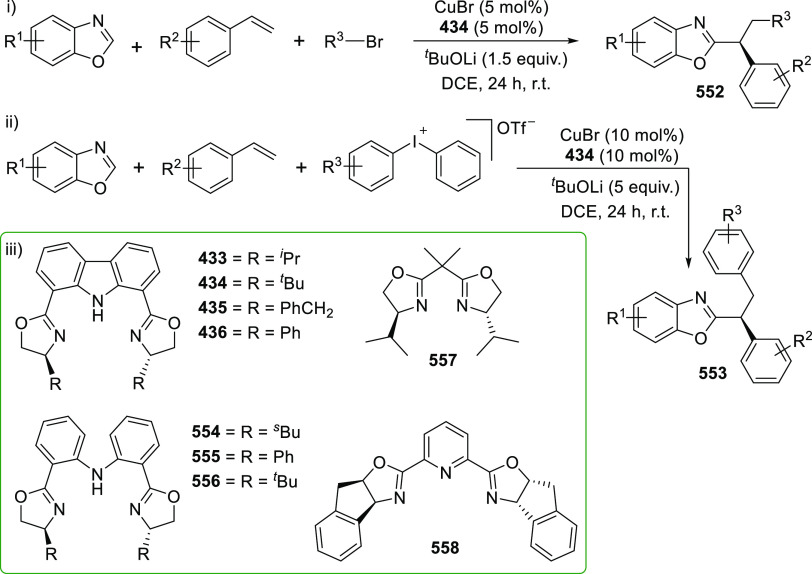
Copper Carbazolide-Mediated Asymmetric Alkyl and Aryl Azolation of Alkenes

For the realization of stereoselective cross-coupling catalyzed reactions, as exemplified by the work of Zhang^410−413^ and Liu et al.,^414−417^ the requirement is an anionic multidentate ligand to enhance the reducing ability of an *in situ* formed copper complex which reduces carboelectrophiles under mild reaction conditions. Furthermore, a demand for stereoselective control on the formed products necessitates the introduction of chiral wingtip **R** groups on the multidentate ligand. These prerequisites, specifically for the asymmetric alkyl and aryl azolation of alkenes, were met by the bis(oxazolinyl)carbazolide scaffold (see sections 4.4 and 5.1).^418^ The authors reported the selective three-component coupling of benzoxazole, styrene, and ethyl 2-bromo-2-methylpropanoate, promoted by the *in situ* formed copper carbazolide complex in the presence of ^*t*^BuOLi (i, Scheme 81). Mediating the reaction with ligands **554**–**556** and **557**–**558** resulted in a significant reduction in both catalytic yield and enantioselectivities (iii, Scheme 81). The results evidenced the requirement for both an anionic coordination site and the rigid carbazole backbone. Moreover, the sterically bulky **434** exhorted the highest degree of control over the catalyzed reaction when compared against the analogues **433**, **435**, and **436**. It was further determined that a range of different heterocyclic systems, alkenes, and alkyl halohydrocarbons could be tolerated while retaining good yields and selectivities (i, Scheme 81). Similarly, the carbazolide-coordinated copper catalyst furnished the three-component coupling between benzoxazole, styrene, and diaryliodonium trifluoromethyl sulfonates with retention of the enantioselectivities and yields (ii, Scheme 81).

**Scheme 82:**
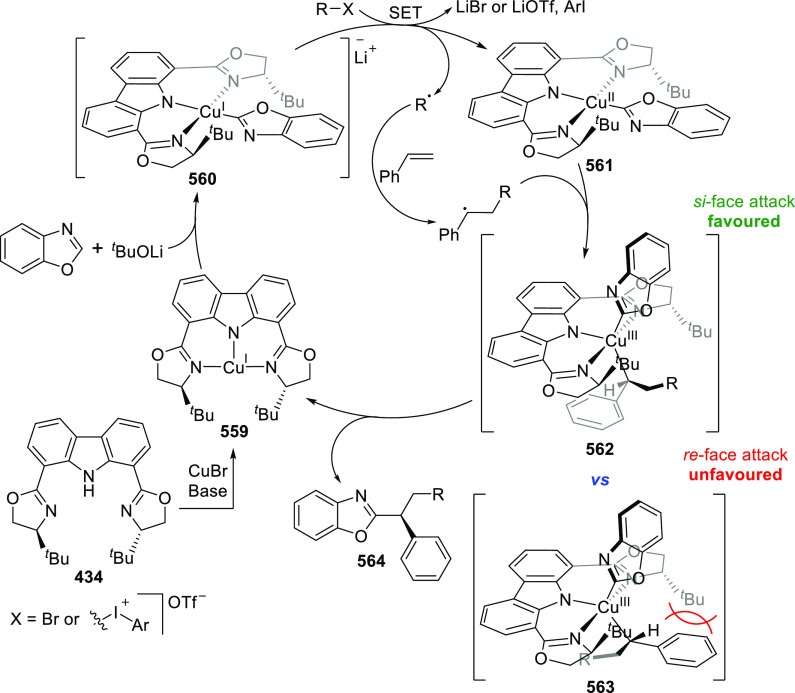
Proposed Catalytic Cycle for the Copper Catalyzed Asymmetric Alkyl and Aryl Azolation of Alkenes

A plausible catalytic reaction mechanism could be elucidated based on various control experiments (Scheme 82).^418^ Base facilitated copper carbazolide formation is followed by coordination of the *in situ* deprotonated azole, leading to intermediate **560**. It was proposed that this intermediate serves as the reductive species, which reacts with either alkyl halide or the diaryliodonium salt leading to a SET event, yielding the oxidized copper(II) intermediate **561** and the alkyl or aryl radical, respectively. Subsequent addition of the alkyl/aryl radical to the alkene furnishes the corresponding radical, which in turn reacts with and results in the oxidized Cu^III^ intermediate **562**. Two proposed Cu^III^ transition states account for the observed selectivity, again dictated by the wingtip steric bulk as has been noted throughout this contribution. Both aryl and alkyl groups experience decreased steric repulsion with *si*-face attack at the metal center, favoring the observed (*S*)-product. The *re*-face transition state **563** is disfavored due to steric repulsion between the substrate and the *tert*-butyl wingtip groups. Finally, reductive elimination ensues, leading to the catalytically active **559** and the targeted product **564**.

**Scheme 83:**
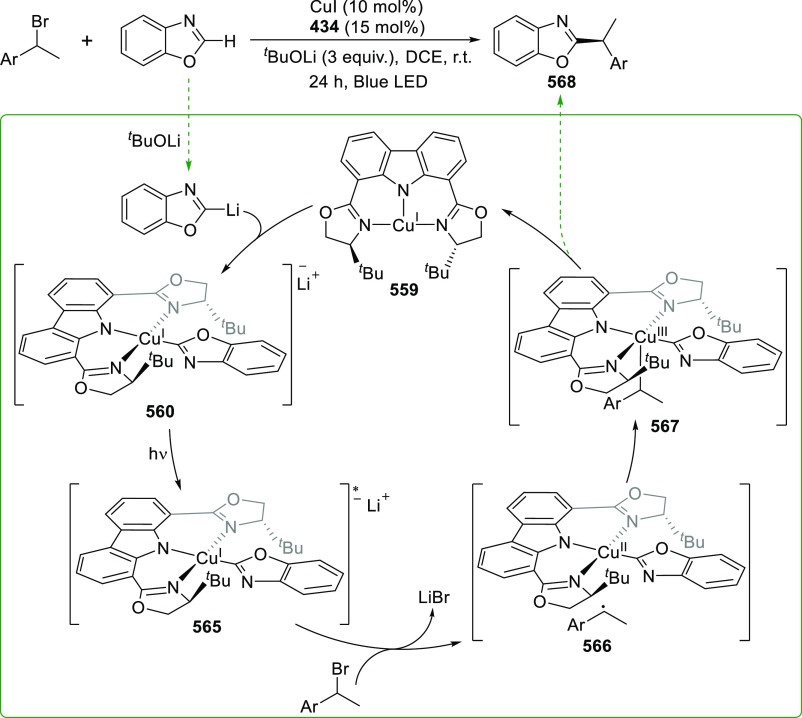
Enantioselective Alkylation of Azoles and Proposed Reaction Mechanism

In the final case study, the group of Zhang also reported on the blue LED-promoted asymmetric-catalyzed alkylation of azoles with aryl-substituted secondary alkyl bromides, yielding the chiral alkyl azoles in high yields and moderate to excellent enantioselectivity (Scheme 83).^419^ The carbazole ligand **434** proved to be the best suited for the catalytic transformation out of the range of ligands tested, which included **433**, **436**, **439**, **440**, **556**, and **558**. It was further reported that the use of green instead of blue LEDs was detrimental to the product yield, but the selectivity remained unchanged. UV–visible light absorption experiments recorded the absence of light absorption of benzoxazole, CuI, and **434** at a wavelength over 400 nm. On the contrary, the copper carbazolide **559** and the intermediate anionic complex **560** both absorbed in the 400–500 nm wavelength range. The excited states of the *in situ* formed **559** and **560** were quenched by benzylic bromide, as determined through Stern–Volmer experiments. The Stern–Volmer constants for **559** and **560** were reported to be 8.8 mM^–1^ and 47 mM^–1^, respectively. The larger Stern–Volmer constants for **560** suggest that it is the photoactive species when irradiated with blue LEDs, while a radical-chain process was ruled out. On the basis of these results, in addition to various control experiments which included deuterium reactions, a catalytic mechanism was proposed (Scheme 83). The *in situ* formation of the copper carbazolide **559** is followed by transmetalation with [Li-azole], obtained from the deprotonation of the azole with ^*t*^BuOLi. This results in the photoactive species **560**, subsequently engaged in photoexcitation which leads to the excited intermediate **565**. Electron transfer to the alkyl bromide ensues, with oxidation of **565** to the Cu^II^ intermediate **566**. Enantioselective radical trapping results in rapid oxidation of **566** to the alkyl coordinated Cu^III^**567**, followed by reductive elimination yielding the optically pure benzylic azole and the copper carbazolide **559** (Scheme 83).^419^ The demonstration of a single 3*d*-transition metal base catalytic system promoting a mild asymmetric coupling reaction via light irradiation is made possible by the use of a redox-stabilizing and photoactive carbazole based pincer ligand for the preparation of the T-shaped catalyst precursor.

### 6.2 Conclusion

In the examples above, the bottom-up approach to assembling a *smart* (switchable, multifunctional, adaptable or tunable)^81^ pincer ligand was shown to provide access to functional molecules, tailored according to the specific requirements for a targeted application. The systematic incorporation of the various design principles available to the **LNL**-carbazolide scaffold, based on the modulation of the steric and electronic effects furnished by the planar, rigid carbazole backbone and the central amido moiety, the flanking donor ligands and their wingtips, provides for a combinatorial effect to address challenges in a wide variety of chemical applications. Particularly the application of this class of pincer ligands in the fast burgeoning area of small molecule activation by the strategies of element-ligand cooperativity^420^ or geometrically constrained elements of the *p*-block^421−423^ is a promising prospect with main group elements coordinated by carbazole-pincer ligands (section 3.3.1) hardly explored.

## Abbreviations

[H-9BBN]_2_: 9-borabicyclo[3.3.1]nonane dimer
[PPN]Cl: bis(triphenylphosphine)iminium chloride
acac: acetyl acetonato
Ad: adamantyl
BIMCA: 3,6-di-*tert*-butyl-1,8-bis(imidazole-2-ylidene-1-yl)carbazolide
bpy: 2,2′-bipyridine
BOPA: bis(oxazolinylphenyl)amine
CASSCF: complete active space self-consistent field
CHC: cyclohexene carbonate
CHO: cyclohexene oxide
CL: caprolactone
COA: cyclooctane
cod: 1,5-cyclooctadiene
COE: cyclooctene
CoPc: cobalt(II) phthalocyanine
Cp: cyclopentadienyl
Cp*: pentamethylcyclopentadienyl
CuAAC: copper-catalyzed azide-alkyne cycloaddition
dba: dibenzylideneacetone
DCE: 1,2-dichloroethane
*de*: diastereomeric excess
DFT: density functional theory
DIPEA: *N*,*N*-diisopropylethylamine
Dipp: 2,6-diisopropylphenyl
DMAP: dimethylaminopyridine
dme: dimethoxyethane
DMF: dimethylformamide
DMPA: 2,2-dimethoxy-2-phenylacetophenone
DMSO: dimethyl sulfoxide
DOSY: diffusion ordered spectroscopy
dppf: 1,1′-bis(diphenylphosphino) ferrocene
dr: diastereoselectivity
*ee*: enantiomeric excess
HOMO: highest occupied molecular orbital
HMDS: hexamethyldisilazane
ILCT: intraligand-charge transfer
IP: isoprene
ISC: intersystem crossing
LDA: lithium diisopropylamide
LMCT: ligand-to-metal charge transfer
LMLCT: ligand/metal-to-ligand charge transfer
LUMO: lowest unoccupied molecular orbital
MBEF: 2-(2-methylidenebut-3-enyl)furan
MC: metal-centered
Mes: 2,4,6-trimethylphenyl
Mes*: 2,4,6-tri-*tert*-butylphenyl
MHD: 3-methylenehepta-1,6-diene
MIC: mesoionic carbene
MLCT: metal-to-ligand charge transfer
MPEP: 2-(3-methylidenepent-4-en-1-yl)pyridine
Nacnac: β-diketiminate
NBS: *N*-bromosuccinimide
NHC: *N*-heterocyclic carbene
NHE: normal hydrogen electrode
NIR: near-infrared spectroscopy
OAc: acetate
OER: oxygen evolution reaction
OLED: organic light emitting diode
OTf: trifluoromethanesulfonate
PCET: proton-coupled electron transfer
PCHC: poly(cyclohexene carbonate)
PCL: polycaprolactone
pet.: petroleum
phen: 1,10-phenenthroline
PIP: polyisoprene
Pipp: *para*-isopropylphenyl
PNHC: protic *N*-heterocyclic carbene
PS: proton sponge
rISC: reverse intersystem crossing
SEC: spectroelectrochemistry
SET: single electron transfer
SIPr: *N*,*N*′-bis(2,6-diisopropylphenyl)-4,5-dihydroimidazol-2-ylidene
SOMO: singly occupied molecular orbital
TADF: thermally activated delayed fluorescence
TBA: *tert*-butylethane
TBACN: tetrabutylammonium cyanide
TBAF: tetrabutylammonium fluoride
TBE: *tert*-butylethylene
^*t*^Boc: *tert*-butoxycarbonyl
TBS: *tert*-butyldimethylsilyl
TBTA: tris(benzyltriazolylmethyl)amine
TEMPO: 2,2,6,6-tetramethylpiperidinyloxyl
TFA: trifluoroacetic acid
THF: tetrahydrofuran
tht: tetrahydrothiophene
TIPS: triisopropylsilyl
TMEDA: tetramethylethylenediamine
TMS: trimethylsilyl
TOF: turnover frequency
Trip: 2,4,6-triisopropylphenyl
WOC: water oxidation catalysis
